# Highly Concentrated Linear Guanidine Amides from the Marine Sipunculid *Phascolosoma granulatum*

**DOI:** 10.1021/acs.jnatprod.3c01186

**Published:** 2024-03-02

**Authors:** Laurence K. Jennings, Navdeep Kaur, Maria C. Ramos, Fernando Reyes, Maggie M. Reddy, Olivier P. Thomas

**Affiliations:** † School of Biological and Chemical Sciences, University of Galway, University Road, Galway H91 TK33, Ireland; ‡ Fundación MEDINA, Centro de Excelencia en Investigación de Medicamentos Innovadores en Andalucía Avda. del Conocimiento 34, Edificio Centro de Desarrollo Farmacéutico y Alimentario, Parque Tecnológico de Ciencias de la Salud, 18016 Granada, Spain; § Department of Biological Sciences, University of Cape Town, Private Bag X3, Rondebosch 7701, South Africa

## Abstract

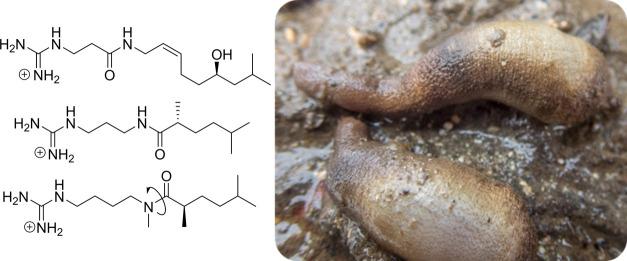

The chemical diversity of annelids, particularly those belonging to the class Sipuncula, remains largely unexplored. However, as part of a Marine Biodiscovery program in Ireland, the peanut worm *Phascolosoma granulatum* emerged as a promising source of unique metabolites. The purification of the MeOH/CH_2_Cl_2_ extract of this species led to the isolation of six new linear guanidine amides, named phascolosomines A–F (**1**–**6**). NMR analysis allowed for the elucidation of their structures, all of which feature a terminal guanidine, central amide linkage, and a terminal isobutyl group. Notably, these guanidine amides were present in unusually high concentrations, comprising ∼3% of the dry mass of the organism. The primary concentration of the phascolosomines in the viscera is similar to that previously identified in linear amides from sipunculid worms and marine fireworms. The compounds from sipunculid worms have been hypothesized to be toxins, while those from fireworms are reported to be defensive irritants. However, screening of the newly isolated compounds for inhibitory bioactivity showed no significant inhibition in any of the assays conducted.

Biodiscovery efforts in Ireland have aimed to explore the understudied chemical diversity within its temperate waters, seeking to find new products with unique commercial and ecological properties. This is achieved through the bioprospection of Irish waters and the creation of an associated library of extracts and fractions for biodiscovery purposes.^1^ The chemical screening of this library employs modern hyphenated methods to assess the unique chemo-diversity present and select specimens for further investigation.^2^ A recent focus of our chemical exploration has been the rich biological diversity of understudied marine organisms, which included annelids in intertidal waters around Ireland. This previously led to the identification of nebulosins, a novel group of thiolane derivatives from an intertidal annelid collected in Galway Bay.^3^ Upon additional MS and NMR-based screening of extracts from a second intertidal annelid, *Phascolosoma granulatum*, once again from Galway Bay—Ireland, remarkably high concentrations of previously unidentified guanidine alkaloids were detected.

Only one previous study has described new compounds isolated from sipunculid worms, reporting two highly concentrated guanidine amides, namely, phascolosomine (**7**) isolated from both *Golfingia vulgaris* and *Golfingia elongata* (reported as *Phascolosoma vulgare* and *Phascolosoma elongatum*), and phascoline (**8**) obtained from *Phascolion strombus* (reported as *Phascolion strombi*).^4,5^ Guanidine-containing metabolites represent a niche group of metabolites within Annelida and are usually associated with phosphogens for energy storage.^6,7^ Interestingly, the guanidine amides **7** and **8** from species of the Sipuncula class were not found to be phosphogens or associated with any primary metabolic function, making them the only guanidine containing secondary metabolites currently known from the Annelida phylum.

Herein, we describe the isolation and structural elucidation of six new linear guanidine amides from the intertidal sipunculid *P. granulatum*. We also compare the distribution and bioactivity of these metabolites to previously identified compounds isolated from Annelida in order to investigate their potential function.

## Results and Discussion

The freeze-dried worm material (14.8 g) was exhaustively extracted with a 1:1 MeOH/CH_2_Cl_2_. The resulting extract (2.41 g) was fractionated using a C_18_*vacuum* liquid chromatography eluting with solvent mixtures of decreasing polarity from H_2_O to MeOH to CH_2_Cl_2_. The polar water–methanol fractions were subjected to semipreparative reversed-phase HPLC purification, yielding six new linear guanidine amides, phascolosomines A–F (**1**–**6**) as their TFA salts.
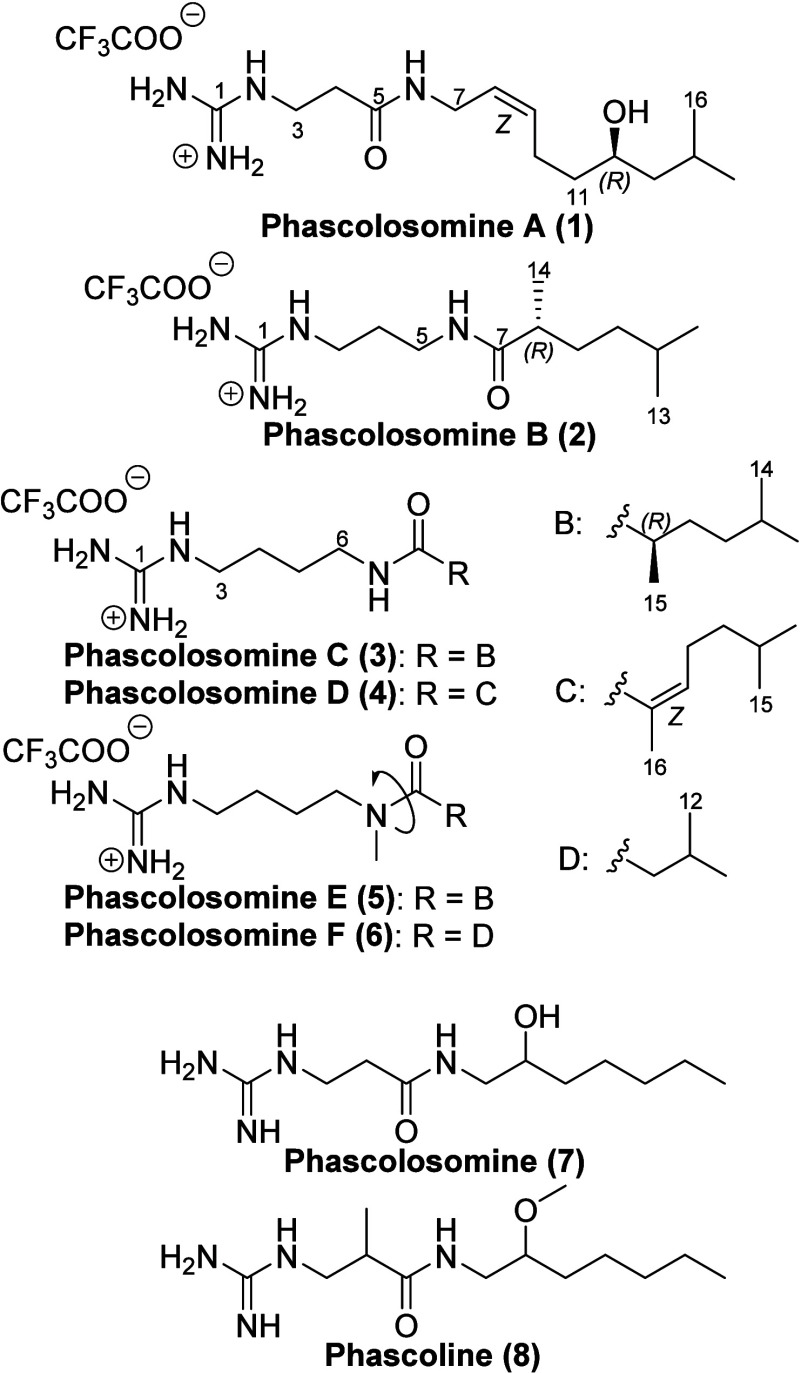


**Figure 1:**
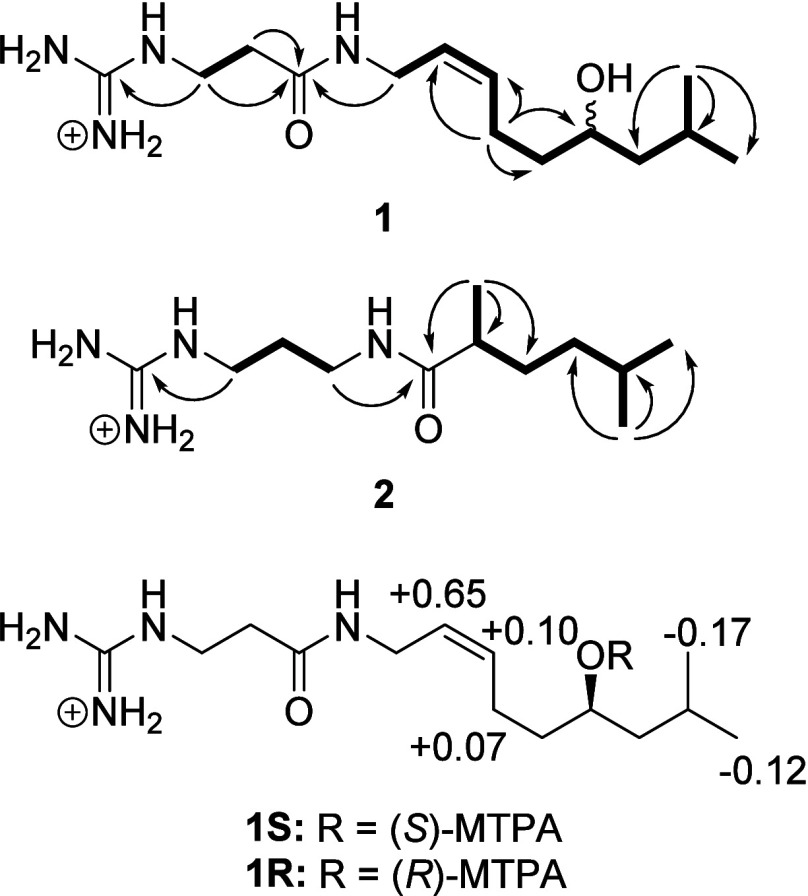
Key HMBC and COSY correlations of **1** and **2**, as well as the absolute configurational analysis of **1** using the Mosher’s ester method Δδ values [Δδ (in ppm) = δ_*S*_– δ_*R*_].

Phascolosomine A (**1**) was isolated as a white amorphous solid and had a protonated adduct at *m*/*z* 285.2292 [M + H]^+^ in the (+)HRESIMS, consistent with the molecular formula C_14_H_29_N_4_O_2_^+^. The ^1^H NMR and HSQC data (Table 1) contained resonances associated with two olefinic protons (δ_H_ 5.54, 5.39), two nitrogen substituted methylenes (δ_H_ 3.87/3.83 and δ_H_ 3.45), one oxygenated methine (δ_H_ 3.59), one methine (δ_H_ 1.78), four methylenes (δ_H_ 2.47, 2.21, 1.48/1.44, and 1.37/1.20), and two methyl doublets (δ_H_ 0.91, 0.90). An initial investigation of the COSY spectrum allowed the assignment of a spin coupled system between two methylene triplets at δ_H_ values of 3.45 (t, *J* = 6.1 Hz, H_2_-3) and 2.48 (t, *J* = 6.1 Hz, H_2_-4). The COSY data then indicated the presence of a second, much larger spin system (Figure 1). Correlations from δ_H_ 5.39 (dtt, *J* = 10.8, 7.6, 1.5 Hz, H-8) to δ_H_ 3.87 (dd, *J* = 15.0, 7.0 Hz, H_2_-7)/3.83 (dd, *J* = 15.0, 7.0 Hz, H_2_-7) and δ_H_ 5.54 (dtt, *J* = 10.8, 6.9, 1.4 Hz, H-9) allowed for the connection of an *N*-methylene to a 1,2-disubstituted olefin. Subsequent correlations from H-9 to δ_H_ 2.21 (q, *J* = 7.7 Hz, H_2_-10) and from the methylene at δ_H_ 1.48/1.44 (m, H_2_-11) to H_2_-10 and δ_H_ 3.59 (m, H-12) established an ethylene link between the olefin and a hydroxy methine. The spin system was completed with a terminal isobutyl group, evidenced by the correlation from H-12 to the ABMX system of a methylene group at δ_H_ 1.37 (ddd, *J* = 14.0, 8.8, and 5.2 Hz, H_2_-13) and δ_H_ 1.20 (ddd, *J* = 13.9, 8.8, and 4.4 Hz, H_2_-13), as well as correlations from the methine at δ_H_ 1.78 (m, H-14) to H_2_-13, δ_H_ 0.91 (d, *J* = 6.5 Hz, H_3_-15) and δ_H_ 0.90 (d, *J* = 6.5 Hz, H_3_-16). The HMBC data then established the amide linkage of the two spin systems with correlations from H_2_-3, H_2_-4 and H_2_-7 to δ_C_ 172.7 (C-5). The HMBC correlation from H_2_-3 to δ_C_ 158.5 (C-1) allowed completion of the planar structure with the assignment of a terminal guanidine unit. The typical 10.8 Hz coupling constant value between the two olefinic resonances, H-8 and H-9, as well as the significantly shielded C-10 methylene substantiated the assignment of the double bond in a *Z* configuration. The absolute configuration of the chiral secondary alcohol at C-12 was then assigned using the Mosher’s ester analysis (Figure 1). The esterification of the C-12 secondary hydroxy with (*R*) and (*S*) MTPA acid chlorides afforded both **1***S* and **1***R*, respectively. The shielding of the signals corresponding to the isopropyl group and deshielding of the olefinic protons in the NMR data of **1***S* when compared to **1***R* allowed the C-12 stereogenic center to be assigned in a 12*R* configuration.

**Table 1:** NMR Spectroscopic Data for Phascolosomines A–D (**1**–**4**) in CD_3_OD

Pos.	Phascolosomine A (**1**)^a^		Phascolosomine B (**2**)^b^		Phascolosomine C (**3**)^b^		Phascolosomine D (**4**)^b^	
	δ_C_, type	δ_H_ (*J* in Hz)	δ_C_, type	δ_H_ (*J* in Hz)	δ_C_, type	δ_H_ (*J* in Hz)	δ_C_, type	δ_H_ (*J* in Hz)
1	158.5,^c^ C		158.7, C		158.6,^c^ C		158.6, C	
3	38.7, CH_2_	3.45, t (6.1)	40.0, CH_2_	3.19, t (6.9)	42.0, CH_2_	3.20, t (7.1)	42.1, CH_2_	3.22, t (6.7)
4	35.8, CH_2_	2.47, t (6.1)	30.1, CH_2_	1.76, quint (6.9)	27.8, CH_2_	1.57, m^d^	27.8, CH_2_	1.62, m^d^
5	172.7, C		37.2, CH_2_	3.24, t (6.9)	27.1, CH_2_	1.60, m^d^	27.2, CH_2_	1.63, m^d^
6					39.3, CH_2_	3.21, t (6.7)	39.5, CH_2_	3.28, t (6.6)
7	37.5, CH_2_	3.83, dd (15.0, 7.0)	180.1, C			8.00, br t		
		3.87, dd (15.0, 7.0)						
8	126.5, CH	5.39, dtt (10.8, 6.9, 1.4)	42.4, CH	2.24, m	179.9, C		173.8, C	
9	133.8, CH	5.54, dtt (10.8, 7.6, 1.5)	33.2, CH_2_	1.60, m	42.5, CH	2.24, ddq (11.1, 9.0, 6.9)	133.5, C	
				1.38, m				
10	24.6, CH_2_	2.21, q (7.7)	37.9, CH_2_	1.19, m^d^	33.2, CH_2_	1.59, m^d^	133.2, CH	5.48, tq (7.5, 1.4)
				1.13, m^d^		1.37, ddt (13.0, 11.2, 5.3)		
11	38.6, CH_2_	1.48, m	29.2, CH	1.53, m^d^	37.9, CH_2_	1.18, m^d^	28.4, CH_2_	2.14, qq (7.5, 1.4)
		1.44, m				1.13, m^d^		
12	69.7, CH	3.59, m	23.0, CH_3_	0.89, d (6.6)	29.2, CH	1.52, m^d^	39.8, CH_2_	1.28, q (7.5)
13	47.9, CH_2_	1.37, ddd (14.0, 8.8, 5.2)	22.9, CH_3_	0.89, d (6.6)	23.0, CH_3_	0.89, d (6.6)	28.8, CH	1.55, m
		1.20, ddd (13.9, 8.8, 4.4)						
14	25.7, CH	1.78, m	18.5, CH_3_	1.10, d (6.9)	22.9, CH_3_	0.88, d (6.6)	22.8, CH_3_	0.89, d (6.7)
15	23.9, CH_3_	0.91, d (6.5)			18.5, CH_3_	1.09, d (6.9)	22.8, CH_3_	0.89, d (6.7)
16	22.4, CH_3_	0.90, d (6.5)					20.9, CH_3_	1.87, q (1.4)

a ^1^H NMR at 600 MHz and ^13^C NMR at 150 MHz.

b ^1^H NMR at 500 MHz and ^13^C NMR at 125 MHz.

c δ_C_ value from HMBC data.

d Signal partially obscured.

**Figure 2:**
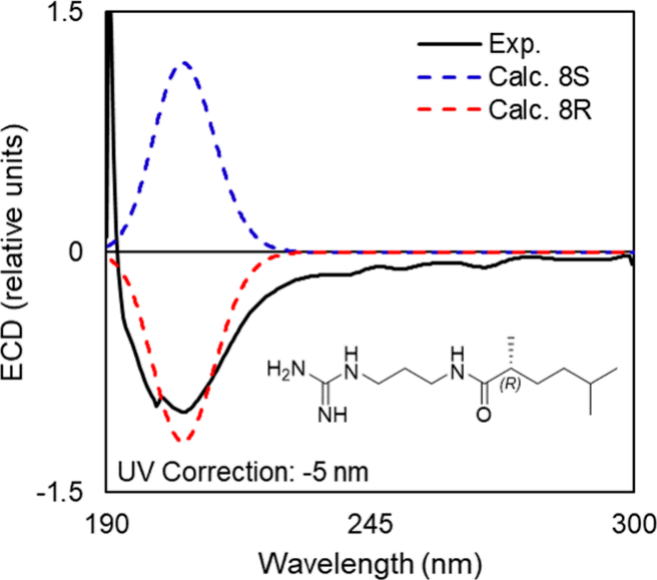
Comparison between the calculated and experimental ECD spectra of **2**.

Phascolosomine B (**2**) was isolated as a white amorphous solid and displayed a protonated adduct at *m*/*z* 243.2188 [M + H]^+^ in the (+)HRESIMS, consistent with the molecular formula C_12_H_27_N_4_O^+^. This compound showed similarities to **1** in the ^13^C NMR data, containing both guanidine (δ_C_ 158.3, C-1) and amide (δ_C_ 179.8, C-7) functional groups. However, significant changes were present in the spin systems linked to these groups (Figure 1). The COSY data indicated a spin system between δ_H_ 3.19 (t, *J* = 6.9 Hz, H_2_-3), δ_H_ 1.75 (quint, *J* = 6.9 Hz, H_2_-4) and δ_H_ 3.23 (t, *J* = 6.9 Hz, H_2_-5). A key HMBC correlation from H_2_-5 to C-7 in addition to the significantly deshielded shift of H_2_-5 allowed for the assignment of the amide in an arrangement opposite to that of **1**. Similar to **1**, the COSY data also indicated the presence of a second larger spin system that contained a terminal isobutyl group. The key COSY correlation between δ_H_ 1.10 (H_3_-15)/δ_H_ 2.24 (H-8)/δ_H_ 1.58, 1.38 (H_2_-9) and δ_H_ 1.18, 1.13 (H_2_-10) then established the new spin system. The HMBC correlations from H_3_-15, H-8 and H_2_-9 to C-7 allowed the small aliphatic chain to be linked to the amide and confirmed its new arrangement. The ECD spectrum of **2** was recorded in water and the absolute configuration of the C-8 stereogenic center could be deduced by comparison with calculated ECD data (Figure 2). The ECD spectra of the enantiomers of **2** were calculated using TDDFT at the M06-2X/6-311+G(2d,p)//B3LYP/6-311G(2d,p) level. The matching negative Cotton effect at ∼200 nm between the experimental and the 8*R* spectra allowed for the assignment of this stereogenic center to finalise the structure of **2**.

Phascolosomine C (**3**) was isolated as a clear oil with a molecular formula of C_13_H_29_N_4_O^+^ consistent with a protonated adduct [M + H]^+^ at *m*/*z* 257.2337 present in the (+)HRESIMS data. This indicated that the only difference between **2** and **3** was the presence of an additional methylene group. The comparison of the ^1^H NMR and COSY NMR data between **2** and **3** allowed for additional methylene to be placed between the guanidine and the amide functional groups, thereby allowing the assignment of an agmatine moiety. The absolute configuration of the C-9 stereogenic center was determined again through the analysis of the experimental ECD spectra. The matching negative Cotton effect at ∼200 nm with the spectra of **2** allowed the assignment of the C-9 stereogenic center in a *R* configuration.

Phascolosomine D (**4**) was isolated as a white amorphous solid and displayed a protonated adduct in the (+)HRESIMS data at *m*/*z* 269.2341 [M + H]^+^ consistent with a molecular formula of C_14_H_29_N_4_O^+^. A comparison of the ^1^H NMR and ^13^C NMR data between **3** and **4** allowed for the assignment of the agmatine unit with an amide linkage. However, this also indicated a different aliphatic chain with a new olefinic methine at δ_H_ 5.48 (tq, *J* = 7.5, 1.4 Hz, H-10) and a deshielded methyl at δ_H_ 1.87 (q, *J* = 1.4 Hz, H_3_-16). The COSY data indicated the presence of an isobutyl moiety similar to that found in **1**–**3** and further correlations between H-10, δ_H_ 2.14 (H_2_-11) and δ_H_ 1.28 (H_2_-12) established a new spin system. The allylic and homoallylic couplings from H_3_-16, as well as HMBC correlations from H_3_-16 to δ_C_ 133.2 (C-10), 133.5 (C-10) and 173.8 (C-8), confirmed its assignment within the structure. Finally, the double bond was assigned in a *Z* configuration due to the presence of a strong ROESY correlation between H-10 and H_3_-16.

Phascolosomine E (**5**) was isolated as a white amorphous solid with a molecular formula of C_14_H_31_N_4_O^+^ inferred by the protonated adduct at *m*/*z* 271.2495 [M + H]^+^ present in the (+)HRESIMS data. The ^1^H NMR and HMBC data indicated that the only difference between **3** and **5** was the *N*-methylation (δ_H_ 3.09/2.92, *N*CH_3_-7) of the amide (Table 2). The ^1^H NMR and ^13^C NMR data also showed the presence of two conformers of **5** in a 3:1 interconverting mixture. This was confirmed by the chemical exchange correlations present in the ROESY between the signals of the different conformers. The ROESY correlations from the two *N*CH_3_ signals identified these as rotamers around the amide bond. The absolute configuration of the C-9 stereogenic center was assigned as *R* due to the negative Cotton effect at ∼200 nm in the ECD spectrum of **5**. The assignment of the same configuration as **2** and **3** is also supported by the identification of the same branched chain that is likely derived from the same biosynthetic pathway.

**Table 2:** NMR Spectroscopic Data for Phascolosomine E and F (**5** and **6**) in CD_3_OD

	Phascolosomine E (**5**)^a^				Phascolosomine F (**6**)^b^			
Pos.	trans		cis		trans		cis	
	δ_C_, type	δ_H_ (*J* in Hz)	δ_C_, type	δ_H_ (*J* in Hz)	δ_C_, type	δ_H_ (*J* in Hz)	δ_C_, type	δ_H_ (*J* in Hz)
1	158.6, C		158.7, C		156.6,^c^ C		156.6,^c^ C	
3	42.1, CH_2_	3.22, t (7.1)	42.1, CH_2_	3.22, t (6.5)	42.0, CH_2_	3.22, t (7.2)	41.8, CH_2_	3.22, t (7.2)
4	26.9, CH_2_	1.54, m^d^	27.1, CH_2_	1.60, m^d^	26.9, CH_2_	1.55, m^d^	27.0, CH_2_	1.60, m^d^
5	25.2, CH_2_	1.62, m^d^	27.1, CH_2_	1.65, m^d^	25.2, CH_2_	1.60, m^d^	26.6, CH_2_	1.64, m^d^
6	48.0, CH_2_	3.42, t (6.7)	50.6, CH_2_	3.43, t (6.6)	47.9, CH_2_	3.42, t (6.6)	50.7, CH_2_	3.42, t (6.8)
7-NMe	35.8, CH_3_	3.09, s	34.2, CH_3_	2.92, s	36.2, CH_3_	3.05, s	33.7, CH_3_	2.92, s
8	179.4, C		179.2, C		175.4, C		175.4, C	
9	37.2, CH	2.80, sext (6.7)	37.0, CH	2.77, sext (6.7)	43.1, CH_2_	2.27, d (6.8)	42.6, CH_2_	2.26, d (6.8)
10	33.2, CH_2_	1.64, m^d^	33.5, CH_2_	1.64, m^d^	26.9, CH	2.10, m	27.1, CH	2.09, m
		1.37, m		1.37, m^d^				
11	37.8, CH_2_	1.18, m^d^	37.9, CH_2_	1.18, m^d^	22.9, CH_3_	0.98, d (6.6)	22.9, CH_3_	0.97, d (6.6)
		1.13, m^d^		1.13, m^d^				
12	29.4, CH	1.54, m^d^	29.5, CH	1.54, m^d^	22.9, CH_3_	0.98, d (6.6)	22.9, CH_3_	0.97, d (6.6)
13	23.0, CH_3_	0.89, d (6.6)	23.0, CH_3_	0.89, d (6.6)				
14	22.9, CH_3_	0.89, d (6.6)	22.9, CH_3_	0.89, d (6.6)				
15	17.9, CH_3_	1.07, d (6.7)	18.6, CH_3_	1.08, d (6.7)				

a ^1^H NMR at 500 MHz and ^13^C NMR at 125 MHz.

b ^1^H NMR at 600 MHz and ^13^C NMR at 150 MHz.

c δ_C_ value from HMBC data.

d Signal partially obscured.

Phascolosomine F (**6**) was isolated as a clear oil. The (+)HRESIMS data displayed a protonated molecule at *m*/*z* 229.2022, [M + H]^+^ consistent with a molecular formula of C_11_H_25_N_4_O^+^. Similar to compound **5** the ^1^H NMR data indicated an interconverting mixture (3:1) consistent with the presence of the *N*-methyl amide signal (δ_H_ 3.06/2.92, *N*CH_3_-7) (Table 2). The only difference between compounds **5** and **6** was the aliphatic chain connected to the amide. The ^1^H NMR and COSY data indicated that an isobutyl moiety was present in **6** with signals of a methylene doublet at δ_H_ 2.27 (H_2_-9), methine at δ_H_ 2.09 (H-11) and a methyl doublet integrating to six protons at δ_H_ 0.98 (H_3_-15 and H_3_-16). The HMBC correlation from H_2_-9 to δ_C_ 175.4 (C-8) then confirmed the incorporation of an isovaleric acid unit within the structure.

While *P. granulatum* contains a diverse array of linear alkaloids, the consistent features among all metabolites are the presence of terminal guanidine and terminal isobutyl moieties connected by an amide-containing chain. The only previously reported metabolites from sipunculid worms were phascolosomine (**7**) and phascoline (**8**) in 1973.^8^ Phascolosomine A (**1**) has a close resemblance with these compounds, featuring the same *N*-amidino-β-alanine unit, but differs due to a more intricate amide-linked alkyl chain. While phascolosomines B–F (**2**–**6**) exhibit similarities to **1**, **7**, and **8**, they differ due to the opposite arrangement of the amide functionality. Compounds **3**–**6** incorporate an agmatine moiety instead of β-alanine, a common substructure found in various similar linear guanidine amides previously isolated from marine invertebrates.^9−13^ Compound **2** is unique due to the inclusion of a rare linear (3-aminopropyl)guanidine moiety that has only been identified in a few marine natural products, such as stellettazoles A and B,^14^ ptilomycalins E and H, and unguiculin A.^15^ Notably, in these previously reported compounds, the (3-aminopropyl)guanidine unit is part of a larger guanidinospermidine unit. A comparison of the phascolosomines with other compounds from Annelida revealed structural similarities with the complanines and the carunculines produced by two marine fireworms, *Eurythoe complanata* and *Hermodice carunculata*, respectively.^16−18^ These fireworm metabolites feature a positively charged trimethylammonium moiety with a similar amide-linked lipid. Moreover, neocomplanine A and carunculines were reported with a terminal isobutyl moiety, a feature also identified in the newly isolated compounds **3** and **4**. The structural similarity may suggest a potential common function, and if so, it would indicate that a positively charged moiety connected to a short lipidic chain through an amide is important for this function.

The origin and biosynthesis of the new phascolosomines are intriguing, considering the presence of the amide identified in both directions. The inverse amide in **2**–**6** suggests the need for different biosynthetic precursors to those required for **1**, **7**, and **8**. A biosynthetic pathway has already been reported for compounds **7** and **8** using isotope-labeled feeding studies.^6^ This pathway concluded that the *N*-amidino-β-alanine moiety was biosynthesized from the degradation of uracil within the sipunculid. Conversely, these studies also determined that the acyclic lipid amine was not produced by the sipunculid and therefore, when sipunculid worms were removed from the environment they ceased production of compounds **7** and **8**.^6,19^ In our study, the same amidino-β-alanine precursor was identified in **1**, however, in **2**–**5** two distinct guanidine biosynthetic precursors are discernible: (3-aminopropyl)guanidine and agmatine. Interestingly, the presence of (3-aminopropyl)guanidine suggests the biosynthesis of **1** and **2** may differ to **7** with the degradation of a polyamine biosynthetic precursor.^20^ We suggest additional feeding experiments be conducted with stable isotopes to identify the new pathways involved.

The similarities of the new phascolosomines to annelid metabolites have sparked intriguing questions about the biological role of these compounds. Various hypotheses for the role of phascolosomine (**7**) and phascoline (**8**) have been formed, with the most recent hypothesis suggesting that they may function as toxins due to their *in vivo* lethality and negative chronotropic activity.^8 ,21 −23^ However, the true functions of these metabolites remain unknown. The complanines and carunculines have excellent data supporting their role as defensive inflammatory irritants.^16,18^ The distribution of phascolosomine (**7**), phascoline (**8**) and the caruculines within their respective organisms has been investigated, and all compounds have been identified at significantly higher concentrations in the viscera tissue.^8,18^ We therefore investigated the distribution of the new phascolosomines A–F (**1**–**6**) within the sipunculid worm to determine their relationship with similar annelid metabolites. Six fresh specimens of *P. granulatum* were dissected to separate the muscular and viscera tissue. All samples were freeze-dried and subject to three extractions with 100% methanol, resulting in an extract enriched with the new guanidine amides. The quantification of these metabolites in the extract was then performed using LC-MS with the purified compounds as standards. These results closely matched the distribution of phascolosomine (**7**) and phascoline (**8**), exhibiting significantly higher concentrations (8.5 times) in the viscera compared to the muscle tissue (Table 3). The most abundant molecule, phascolosomine E (**5**), was identified in the viscera at a high concentration of 3800 mg/100 g of dry mass. These concentrations are similar to those of **7** and **8**, which were reported in the viscera at 615 and 1100 mg/100 g of wet weight, respectively. This congruent distribution pattern and concentration with previously reported metabolites strongly suggests that the new phascolosomines likely share a similar biological role. However, despite this further similarity to **7** and **8**, the function of these phascolosomines remains elusive. However, the fact that these metabolites constitute an average of almost 3% of the dry mass of the entire organism suggests that they must serve a substantial and vital biological function. Additionally, given the similarities between the inflammatory irritants from fireworms and the guanidine amides from sipunculid worms, we suggest the need for a much broader study to investigate this large family of annelid amides.

**Table 3:** Concentrations of **1**–**6** in the Dissected Muscle and Viscera of *Phascolosoma granulatum* Specimens Quantified by LC-MS^a^

	concentration (mg/100 g dry mass) ± SE			
compound	muscle	viscera	whole body	ratio V/M
1	1.1 ± 0.3	8.3 ± 1	4.5 ± 0.9	7.7
2	38 ± 6	290 ± 40	160 ± 30	7.8
3	41 ± 8	320 ± 30	180 ± 30	7.8
4	30. ± 4	280 ± 40	160 ± 3	9.3
5	440 ± 60	3800 ± 250	2000. ± 200	8.6
6	0.8 ± 0.2	20. ± 4	10. ± 3	25

a Note: standard errors are from the specimen variability.

Considering the known antimicrobial and toxic properties of the coelomic fluid of sipunculid worms,^5,24^ as well as other marine-derived linear guanidine amides, including phascolosomine (**7**) and phascoline (**8**),^6^ the newly discovered phascolosomines **1**–**6** underwent a comprehensive assessment for a broad range of inhibitory bioactivities. In this study, the six new guanidine amides were screened for toxicity against *Artemia salina*, cytotoxicity against five human tumor cell lines, and antimicrobial activity against six fungal and three bacterial pathogens. Surprisingly, none of the reported compounds exhibited inhibitory activity in any of the assays conducted at the highest concentrations tested (20 μg/mL for tumor assays, 64 μg/mL for antimicrobial assays and 500 μM for *A. salina* assays). This lack of inhibitory activity against a diverse range of organisms led us to believe that the phascolosomines and other linear guanidine amides from the class Sipuncula are not likely to be lethal toxins as previously suggested. Whether they have bioactivity related to a deterrent role within the sipunculid, more in line with compounds from fireworms, remains to be identified. Complanine was suggested to bind to the phospholipid binding site of protein kinase C, thereby having an inflammatory effect.^16^ Given the similar hydrophilic charged terminal and hydrophobic lipid units in the newly identified phascolosomines, a similar binding would not be surprising.

Phascolosomine (**7**) and phascoline (**8**) are reported to be unimportant for the health or survival of the organism,^6^ which suggests that they must fulfill some secondary metabolic function. The identification of the new phascolosomines **1**–**6** in high concentrations and lack of bioactivity observed in this study further suggests these compounds must have a specific secondary metabolic function within the sipunculid worm. Further investigation is warranted to unravel the specific role of these compounds within sipunculid worms.

## Experimental Section

### General Experimental Procedures

Optical rotations were recorded at the sodium D-line (589.3 nm) on a Unipol L1000 polarimeter with a 10 cm cell at 20 °C (Schmidt+Haensch, Berlin, Germany). UV and ECD data were recorded in water on a Chirascan V100 with a 1.0 cm quartz cuvette (Applied Photophysics, Leatherhead, U.K.). IR data was recorded on a PerkinElmer spectrum 100 FT-IR spectrometer (Massachusetts, U.S.A.). NMR experiments were performed on a 500 MHz Varian Inova spectrometer with a 5 mm OneNMR probe and a 600 MHz Agilent Premium Compact spectrometer with a 5 mm CryoProbe (Agilent, Santa Clara, U.S.A.). The chemical shifts (δ in ppm) are referenced to the carbon (δ_C_ 49.00) and proton (δ_H_ 3.31) signals of residual MeOD-*d*_4_ within the NMR solvent. High-resolution mass spectra (HRESIMS) were obtained using an Agilent 6540 Q-Tof mass spectrometer equipped with an Agilent 1290 UPLC and autosampler (Agilent). Large scale RP-SPE fractionation was performed using polygoprep C_18_-bonded silica 35–60 μm, 120 Å (Labquip, Ireland). Semipreparative HPLC was carried out on an Agilent 1260 HPLC system equipped with a DAD detector. All solvents used for extraction and separations were HPLC grade, and H_2_O was milli-Q filtered. Trifluoroacetic acid (TFA) was used for HPLC separation and was spectroscopy grade from Alfa Aesar.

### Biological Material

The intertidal sipunculid *Phascolosoma granulatum* was collected by hand on June 17th, 2019 at Newquay (Co. Clare, Ireland) during low tide. Specimens were identified to be burrowed in rocks along the shore with the anterior mouth exposed. The specimen was identified through the DNA barcoding of the COI gene. The sequence was deposited in GenBank for future reference (ON322725). A voucher specimen of an individual specimen, “BDV10161”, is stored at the Marine Biodiscovery Laboratory (University of Galway).

### Extraction and Isolation

The freeze-dried biomass (14.80 g) was extracted with a solvent mixture of 1:1 (*v*/*v*) MeOH/CH_2_Cl_2_ under sonication to yield an extract (2.41 g). The extract was then fractionated using reverse-phase vacuum liquid chromatography on C_18_-bonded silica, eluting with varying solvent mixtures to yield five fractions, 100% H_2_O, 50% H_2_O/50% MeOH (320 mg), 25% H_2_O/75% MeOH (36.5 mg), 100% MeOH (154 mg), 50% MeOH/50% CH_2_Cl_2_ (380 mg). The 100% MeOH fraction was then separated using reversed phase semipreparative HPLC purification on a Waters symmetry C_18_ prep, 7 μm, 7.8 × 250 mm column. The column was first eluted with 70% H_2_O (0.1% TFA)/30% CH_3_CN (0.1% TFA) for 5 min, followed by a linear gradient to 63% H_2_O (0.1% TFA)/36% CH_3_CN (0.1% TFA) over 18 min. Finally, a linear gradient to 100% CH_3_CN (0.1% TFA) was observed over 2 min, with the column then further eluted with these conditions for 10 min all at a flow rate of 2.2 mL/min. This yielded the new phascolosomine A (**1**, 1.1 mg), B (**2**, 2.1 mg), C (**3**, 3.9 mg), D (**4**, 2.4 mg), and E (**5**, 17.8 mg).

The 50% MeOH fraction was separated using reverse phase semipreparative HPLC purification on a Waters symmetry C_18_ prep, 7 μm, 7.8 × 250 mm column. The column was first eluted with 90% H_2_O (0.1% TFA)/10% CH_3_CN (0.1% TFA) for 5 min, followed by a linear gradient to 40% H_2_O (0.1% TFA)/60% CH_3_CN (0.1% TFA) over 20 min. Finally, a linear gradient to 100% CH_3_CN (0.1% TFA) over 5 min was performed, and the column was further eluted at these conditions for 7 min, all at a flow rate of 3.0 mL/min. This again yielded phascolosomines A–E with the addition of the new phascolosomine F (**6**, 1.9 mg).

#### Phascolosomine A (**1**)

White crystalline solid; [α]_20_^D^ −6.5 (*c* 0.02, MeOH); UV/vis (H_2_O) λ_max_ (log ε) 190 (3.63) nm; ^1^H and ^13^C NMR data see Table 1; ^1^H NMR (C_5_D_5_N, 500 MHz) δ_H_ 9.64 (br t, *J* = 6.5 Hz, NH-2), 9.09 (t, *J* = 5.0 Hz, NH-6), 8.89 (br, 1-NH_2_, 5.67 (m, H-8/H-9), 4.22 (dt, *J* = 14.6, 5.0 Hz, H-7), 4.18 (dt, *J* = 14.6, 5.0 Hz, H-7), 3.88 (tt, *J* = 8.5, 3.9 Hz, H-12), 3.82 (q, *J* = 6.3 Hz, H_2_-3), 2.80 (t, *J* = 6.3 Hz, H_2_-4), 2.47 (q, *J* = 7.2 Hz, H_2_-10), 1.77 (m, H-14), 1.68 (m, H-11), 1.64 (m, H-11), 1.59 (m, H-13), 1.30 (m, H-13), 0.96 (d, *J* = 6.5 Hz, H_3_-15), 0.94 (d, *J* = 6.5 Hz, H_3_-16); HSQC NMR (C_5_D_5_N, 500 MHz, partial ^13^C data) 132.9 (C-9), 126.2 (C-8), 68.0 (C-12), 47.4 (C-13), 38.2 (C-11), 38.1 (C-3), 36.8 (C-7), 35.5 (C-4), 27.5 (C-14), 24.0 (C-10), 20.9 (C-15), 20.1 (C-16); (+)-HRESIMS *m*/*z* 285.2292 [M + H]^+^ (calcd for C_14_H_29_N_4_O_2_^+^, 285.2285, Δ +2.5 ppm).

#### Phascolosomine B (**2**)

Clear, oil; [α]_20_^D^ −11.3 (*c* 0.08, MeOH); UV/vis (H_2_O) λ_max_ (log ε) 192 (4.24) nm; ECD (*c* 1.4 × 10^–4^ M, H_2_O) λ_max_ (*Δε*) 206 (−0.22) nm; ^1^H and ^13^C NMR data see Table 1; (+)HRESIMS *m*/*z* 243.2188 [M + H]^+^ (calcd for C_14_H_29_N_4_O_2_^+^, 243.2179, Δ +3.7 ppm).

#### Phascolosomine C (**3**)

Clear, oil; [α]_20_^D^ −2.5 (*c* 0.08, MeOH); UV/vis (H_2_O) λ_max_ (log ε) 190 (4.28) nm; ECD (*c* 1.3 × 10^–4^ M, H_2_O) λ_max_ (Δε) 209 (+0.04), 193 (−0.27) nm; ^1^H and ^13^C NMR data see Table 1; (+)HRESIMS *m*/*z* 257.2337 [M + H]^+^ (calcd for C_14_H_29_N_4_O_2_^+^, 257.2336, Δ +0.4 ppm).

#### Phascolosomine D (**4**)

Clear, oil; UV/vis (H_2_O) λ_max_ (log ε) 190 (4.14) nm; IR (film) *v*_max_ 3331, 2952, 1690, 1154, 1111, 980 cm^–1^; ^1^H NMR and ^13^C NMR data see Table 1; (+)HRESIMS *m*/*z* 269.2341 [M + H]^+^ (calcd for C_14_H_29_N_4_O_2_^+^, 269.2336, Δ +1.9 ppm).

#### Phascolosomine E (**5**)

Clear, oil; [α]_20_^D^ −2.3 (*c* 1.08, MeOH); UV/vis (H_2_O) λ_max_ (log ε) 192 (4.31) nm; ECD (*c* 1.3 × 10^–4^ M, H_2_O) λ_max_ (Δε) 220 (+0.13), 200 (−0.36) nm; IR (film) *v*_max_ 3345, 2954, 1654, 1429, 1189, 1121 cm^–1^; ^1^H NMR and ^13^C NMR data see Table 1; (+)HRESIMS *m*/*z* 271.2495 [M + H]^+^ (calcd for C_14_H_29_N_4_O_2_^+^, 271.2492, Δ +1.1 ppm).

#### Phascolosomine F (**6**)

Clear, oil; UV/vis (H_2_O) λ_max_ (log ε) 190 (3.83) nm; ^1^H and ^13^C NMR data see Table 1; (+)HRESIMS *m*/*z* 229.2022 [M + H]^+^ (calcd for C_14_H_29_N_4_O_2_^+^, 229.2023, Δ −0.4 ppm).

### Computational Methods

Conformational analyses of each compound were performed with Schrodinger MacroModel. This conformer generation was performed using the OPLS3 force field with water as the selected solvent. Due to the flexibility of the molecule, the energy window was decreased to 3.0 kcal/mol, and the maximum atom deviation was increased to 1 Å for conformer generation to decrease the numbers of redundant conformers. The 13 conformers generated were further optimized using DFT, at the M026X/6-311+G(2d,p) level in *Gaussian 16*, and at the same time, the free energy of each conformers was calculated.^25^ The rotational strength for each conformer was then calculated by using TDDFT in *Gaussian 16* at the B3LYP/6-311G(2d,p) level for 50 excited states. A polarizable continuum solvation model was used with water as a solvent for all DFT calculations.^26^ The ECD spectra of all conformers were Boltzmann weighted based on the free-energy, combined, and corrected with the experimental UV spectra in the freely available software SpecDis 1.7.^27^

### Preparation of the MTPA Esters of Phascolosomine A (**1**)

The method used was based on the methods previously described by Hoye et al.^28^ Due to the solubility of **1**, dry deuterated pyridine was used instead of a chlorinated solvent, and the reaction was carried out in a dry nitrogen atmosphere. Phascolosomine A (**1**, 0.3 mg, 1.05 μmol) was dissolved in dry deuterated pyridine (300 μL, 3.5 mM) in a 2 mL vial. The *S*-(+)-α-methoxy-α-trifluoromethylphenylacetic acid chloride (*S*-(+)-MTPA-Cl) (1.98 μL, 10.50 μmol, 10 equiv) was then added at room temperature. After 2 h, the reaction mixture was transferred directly to a dry 5 mm NMR tube and monitored by ^1^H NMR. After completion of the reaction, the ^1^H NMR and COSY spectra of *R*-MTPA-**1** ester (**1***R*) were acquired. **1***R*: ^1^H NMR (C_5_D_5_N) δ_H_ 5.41 (dt, *J* = 10.7, 7.2 Hz, H-9), 5.09 (m, H-8), 2.20 (m, H-10), 1.60 (m, H-11), 4.38 (m, H-12), 1.76 (m, H-14), 0.93 (d, *J* = 6.6 Hz, H_3_-15), 0.92 (d, *J* = 6.6 Hz, H_3_-16). Using analogous methodology, the *S*-MTPA-**1** ester (**1***S*) was prepared by using *R*-(−)-MTPA-Cl. ^1^H NMR and COSY spectra of the mixture containing **1***S* were acquired after the completion of the reaction. **1***S*: ^1^H NMR (C_5_D_5_N) δ_H_ 5.74 (dt, *J* = 10.5, 6.7 Hz, H-8), 5.51 (dt, J = 10.5, 7.2 Hz, H-9), 2.27 (m, H-10), 1.81 (m, H-11a), 1.72 (m, H-11b), 4.35 (m, H-12), 1.45 (m, H-14), 0.81 (d, *J* = 6.6 Hz, H_3_-15), 0.75 (d, *J* = 6.6 Hz, H_3_-16).

### Metabolite Distribution Analysis

Six fresh specimens of *P. granulatum* were collected from the same location along the Newquay shore, Co. Clare, Ireland. Directly after collection the viscera and the muscular tissue of each specimen were separated by dissection, and the resulting biomass was freeze-dried, resulting in 12 tissue samples for analysis. The tissue sample was weighed before being sonicated with 2 mL of MeOH. This was centrifuged to separate the extract from solids, and the supernatant was passed through a 0.2 μm filter. This extraction was performed three times, and the resulting extract was dried, weighed, and prepared for LC-MS in 1 mL of MeOH. The pure phascolosomines (**1**–**6**) were prepared in MeOH at differing concentrations to ensure a similar sample concentration range and calibration range (**1**: 0.03–1 μg/mL, **2**–**4**, **6**: 0.19–6 μg/mL, **5**: 1–30 μg/mL). LC-MS analysis was performed on a Waters equity UHPLC BEH C_18_, 1.7 μm, 2.1 mm × 75 mm column. The column was eluted with 0.4 mL/min of 90% H_2_O (0.1% FA)/10% MeCN (0.1% FA) for 2 min, followed by a linear gradient to 100% MeCN (0.1% FA) over 6 min, and further eluted at this condition for 4 min. The MS detection was acquired in +ESI mode; overlaid extracted ion chromatograms of the specimen extracts are present in the Supporting Information (Figures S7-7 to S7-12). Compounds **1**–**6** were then quantified using the Agilent MassHunter Quantification software package (Agilent, Santa Clara, U.S.A.). This provides a standardized integration of peaks with matching retention time and exact mass from an extracted ion chromatogram. This also formed calibration curves (Figures S7-1 to S7-6) from the integration of standards, thereby providing compound concentrations (Table S1).

### Biological Screening

Antimicrobial assays against *C. albicans*, *C. glabrata*, *C. krusei*, *C. parapsilosis*, *C. tropicalis*, *A. fumigatus*, *S. aureus*, *E. coli*, and *A. baumannii* were conducted following previously published procedures.^29,30^ Compounds were assayed at 2-fold dose response curves starting at 64 μg/mL (Tables S4 and S5).

Cell viability assays against five different human cancer cell lines A549 (lung carcinoma, CRM-CCL-185TM, ATCC), A2058 (metastatic melanoma, CRL-3601TM, ATCC), MCF-7 (breast adenocarcinoma, HTB-22, ATCC), MIA PaCa-2 (pancreatic carcinoma, CRL-1420, ATCC), and HepG2 (hepatocyte carcinoma, HB-8065, ATCC) were performed using an MTT (3-(4,5-dimethylthiazol-2-yl)-2,5-diphenyltetrazolium bromide) assay.^31^ Compounds were assayed at 2-fold dose response curves starting at 20 μg/mL (Table S3).

The brine shrimp toxicity assay was performed following previously publish methods.^32^ The *Artemia salina* culture was prepared from commercially available freeze-dried cysts. Assays were performed in 96-well plates with a working volume of 200 μL of artificial seawater containing ∼15–20 shrimp in each well. Compounds were added to each well in DMSO to final concentrations between 500 and 0.4 μM. Plates were then incubated at 25 °C in constant light for 48 h. The number of dead nauplii were counted with a microscopy every 24 h. Potassium dichromate was used as a positive control and DMSO as a negative control. At the end of the experiment, this was converted into a percentage of dead nauplii, corrected with DMSO control, and the LD_50_ of compounds was calculated. Each assay was conducted with six replicates.

## Supporting Information Available

1D and 2D NMR spectra of phascolosomines A–F (**1**–**6**), MS quantification results, bioactivity results, and DFT optimized coordinates. All raw NMR data are deposited in NP-MRD (PDF)
