# Supplementary material for: Highly Concentrated Linear Guanidine Amides from the Marine Sipunculid Phascolosoma granulatum
Source: J Nat Prod. 2024 Mar 2;87(4):906–13. doi: 10.1021/acs.jnatprod.3c01186 (PMC11061827; doi:10.1021/acs.jnatprod.3c01186)
Supplement: Supplementary file 1 — np3c01186_si_001.pdf [file np3c01186_si_001.pdf]

Supplementary Data

Highly Concentrated Linear Guanidine Amides from the Marine Sipuncula *Phascolosoma granulatum*.

Laurence K. Jennings,[†] *Navdeep Kaur,*[†] *Maria C. Ramos,*[‡] *Fernando Reyes,*[‡] *Maggie M. Reddy,*[†,§] *Olivier P. Thomas*[†*]

[†] School of Biological and Chemical Sciences, University of Galway, University Road, Galway H91 TK33. Ireland.

[‡] Fundación MEDINA, Centro de Excelencia en Investigación de Medicamentos Innovadores en Andalucía Avda. del Conocimiento 34, Edificio Centro de Desarrollo Farmacéutico y Alimentario, Parque Tecnológico de Ciencias de la Salud, 18016 Granada, Spain.

[§] Department of Biological Sciences, University of Cape Town, Private Bag X3, Rondebosch 7701, South Africa.

* olivier.thomas@universityofgalway.ie

Contents:

Figure S1-1. ^{1}H NMR spectrum (600 MHz) of **1** in MeOH-d_4. Page 4
Figure S1-2. ^{13}C NMR spectrum (150 MHz) of **1** in MeOH-d_4. Page 4
Figure S1-3. COSY spectrum (600 MHz) of **1** in MeOH-d_4. Page 5
Figure S1-4. Edited HSQC spectrum (600 MHz) of **1** in MeOH-d_4. Page 5
Figure S1-5. HMBC spectrum (600 MHz) of **1** in MeOH-d_4. Page 6
Figure S1-6. ECD and UV spectrum (c 0.25 mM) of **1** in H_2O. Page 6

Figure S2-1. ^{1}H NMR spectrum (500 MHz) of **2** in MeOH-d_4. Page 7
Figure S2-2. ^{13}C NMR spectrum (125 MHz) of **2** in MeOH-d_4. Page 7
Figure S2-3. COSY spectrum (500 MHz) of **2** in MeOH-d_4. Page 8
Figure S2-4. Edited HSQC spectrum (500 MHz) of **2** in MeOH-d_4. Page 8
Figure S2-5. HMBC spectrum (500 MHz) of **2** in MeOH-d_4. Page 9
Figure S2-6. ECD and UV spectrum (c 0.14 mM) of **2** in H_2O. Page 9

Figure S3-1. ^{1}H NMR spectrum (500 MHz) of **3** in MeOH-d_4. Page 10
Figure S3-2. ^{13}C NMR spectrum (125 MHz) of **3** in MeOH-d_4. Page 10
Figure S3-3. COSY spectrum (500 MHz) of **3** in MeOH-d_4. Page 11
Figure S3-4. Edited HSQC spectrum (500 MHz) of **3** in MeOH-d_4. Page 11
Figure S3-5. HMBC spectrum (500 MHz) of **3** in MeOH-d_4. Page 12
Figure S3-6. ECD and UV spectrum (c 0.13 mM) of **3** in H_2O. Page 12

Figure S4-1. ^{1}H NMR spectrum (500 MHz) of **4** in MeOH-d_4. Page 13
Figure S4-2. ^{13}C NMR spectrum (125 MHz) of **4** in MeOH-d_4. Page 13
Figure S4-3. COSY spectrum (500 MHz) of **4** in MeOH-d_4. Page 14
Figure S4-4. Edited HSQC spectrum (500 MHz) of **4** in MeOH-d_4. Page 14
Figure S4-5. HMBC spectrum (500 MHz) of **4** in MeOH-d_4. Page 15
Figure S4-6. ROESY spectrum (500 MHz) of **4** in MeOH-d_4. Page 15
Figure S4-7. UV spectrum (c 1.90 mM) of **4** in H_2O. Page 16

Figure S5-1. ^{1}H NMR spectrum (500 MHz) of **5** in MeOH-d_4. Page 17
Figure S5-2. ^{13}C NMR spectrum (125 MHz) of **5** in MeOH-d_4. Page 17
Figure S5-3. COSY spectrum (500 MHz) of **5** in MeOH-d_4. Page 18
Figure S5-4. Edited HSQC spectrum (500 MHz) of **5** in MeOH-d_4. Page 18
Figure S5-5. HMBC spectrum (500 MHz) of **5** in MeOH-d_4. Page 19
Figure S5-6. ROESY spectrum (500 MHz) of **5** in MeOH-d_4. Page 19
Figure S5-7. ECD and UV spectrum (c 1.08 mM) of **5** in H_2O. Page 20

Figure S6-1. ^{1}H NMR spectrum (600 MHz) of **6** in MeOH-d_4. Page 21
Figure S6-2. ^{13}C NMR spectrum (150 MHz) of **6** in MeOH-d_4. Page 21
Figure S6-3. COSY spectrum (600 MHz) of **6** in MeOH-d_4. Page 22
Figure S6-4. Edited HSQC spectrum (600 MHz) of **6** in MeOH-d_4. Page 22
Figure S6-5. HMBC spectrum (600 MHz) of **6** in MeOH-d_4. Page 23
Figure S6-6. UV spectrum (c 0.15 mM) of **6** in H_2O. Page 23

Figure S7-1. Overlaid LC-(+)HRESIMS base peak chromatograms of dilutions of a mixture of known concentrations of phascolosomines A-F (**1-6**). Page 24

Figure S7-1. Overlaid extracted ion chromatogram of standards, MS spectrum and calibration curve for phascolosomine A (**1**). Page 25
Table S1. Quantification data for phascolosomine A (**1**). Page 25
Figure S7-2. Overlaid extracted ion chromatogram of standards, MS spectrum and calibration curve for phascolosomine B (**2**). Page 26
Table S2. Quantification data for phascolosomine B (**2**). Page 26
Figure S7-3. Overlaid extracted ion chromatogram of standards, MS spectrum and calibration curve for phascolosomine C (**3**). Page 27
Table S3. Quantification data for phascolosomine C (**3**). Page 27
Figure S7-4. Overlaid extracted ion chromatogram of standards, MS spectrum and calibration curve for phascolosomine D (**4**). Page 28
Table S4. Quantification data for phascolosomine D (**4**). Page 28
Figure S7-5. Overlaid extracted ion chromatogram of standards, MS spectrum and calibration curve for phascolosomine E (**5**). Page 29
Table S5. Quantification data for phascolosomine E (**5**). Page 29
Figure S7-6. Overlaid extracted ion chromatogram of standards, MS spectrum and calibration curve for phascolosomine F (**6**). Page 30
Table S6. Quantification data for phascolosomine F (**6**). Page 30
Figure S7-7. Overlaid extracted ion chromatograms for phascolosomines A-F (**1-6**) of the viscera and muscle extract of *P. granulatum* specimen 1. Page 31
Figure S7-8. Overlaid extracted ion chromatograms for phascolosomines A-F (**1-6**) of the viscera and muscle extract of *P. granulatum* specimen 2. Page 31
Figure S7-9. Overlaid extracted ion chromatograms for phascolosomines A-F (**1-6**) of the viscera and muscle extract of *P. granulatum* specimen 3. Page 31
Figure S7-10. Overlaid extracted ion chromatograms for phascolosomines A-F (**1-6**) of the viscera and muscle extract of *P. granulatum* specimen 4. Page 32
Figure S7-11. Overlaid extracted ion chromatograms for phascolosomines A-F (**1-6**) of the viscera and muscle extract of *P. granulatum* specimen 5. Page 32
Figure S7-12. Overlaid extracted ion chromatograms for phascolosomines A-F (**1-6**) of the viscera and muscle extract of *P. granulatum* specimen 6. Page 32
Table S7. Quantification of compounds **1-6** for six dissected *Phascolosoma granulatum* specimens (A-F) using (+)HRESIMS. Page 33

Table S2. LD_{50} activity for toxicity in brine shrimp lethality assay. Page 34
Table S3. AC_{50} activity for cytotoxicity against multiple human cancer cell lines. Page 34
Table S4. MIC antifungal activity against multiple fungal strains. Page 34
Table S5. MIC antibacterial activity against bacterial strains. Page 34
Figure S8. A plausible biosynthetic pathway of the three guanidine moieties (blue) required for the biosynthesis of phascolosomines A-F, (**1**– **6**) from polyamine precursors. Page 35
Data 1. Gaussian script used for the prediction of ECD in Gaussian 16. Page 36
Data 2. Geometry optimised Cartesian coordinates and energy calculations for each conformer predicted for phascolosomine B (**2**). Page 37

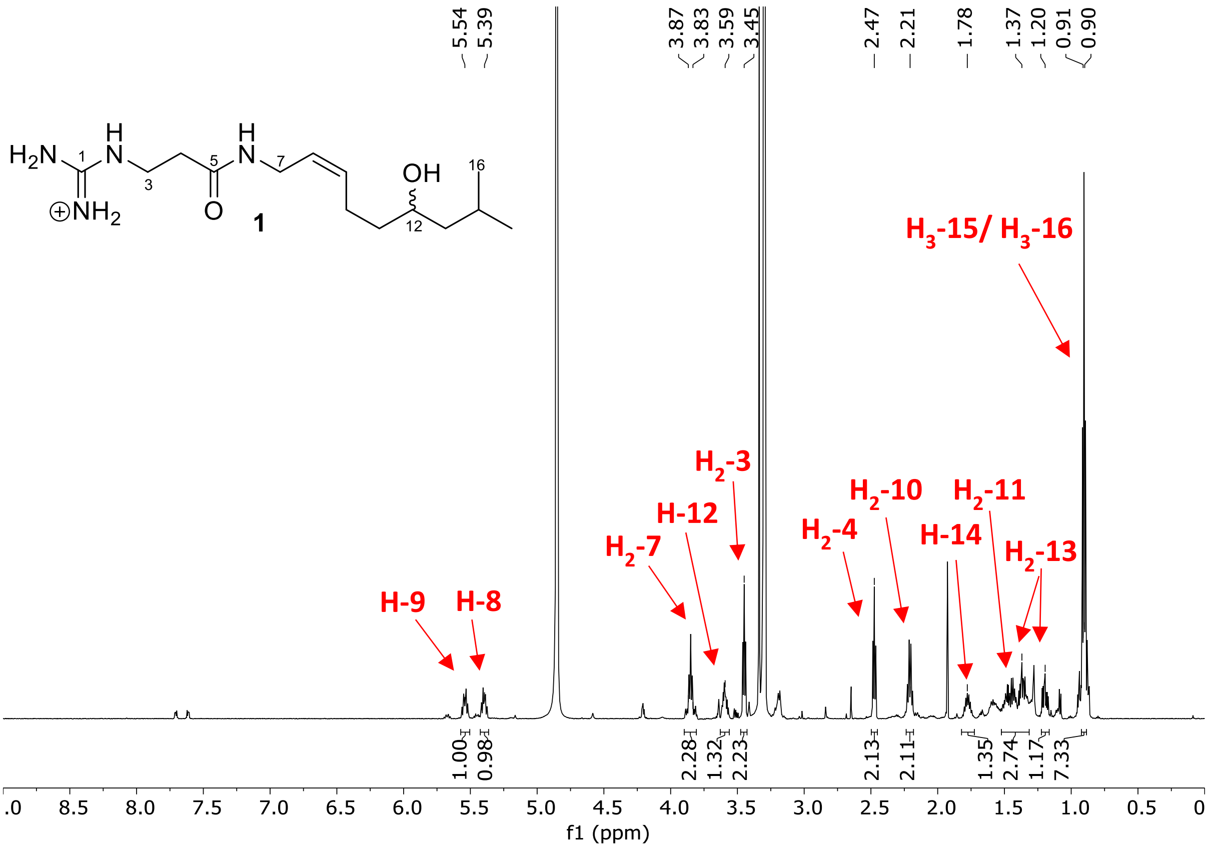

Figure S1-1. ^{1}H NMR spectrum (600 MHz) of **1** in MeOH-d_4.

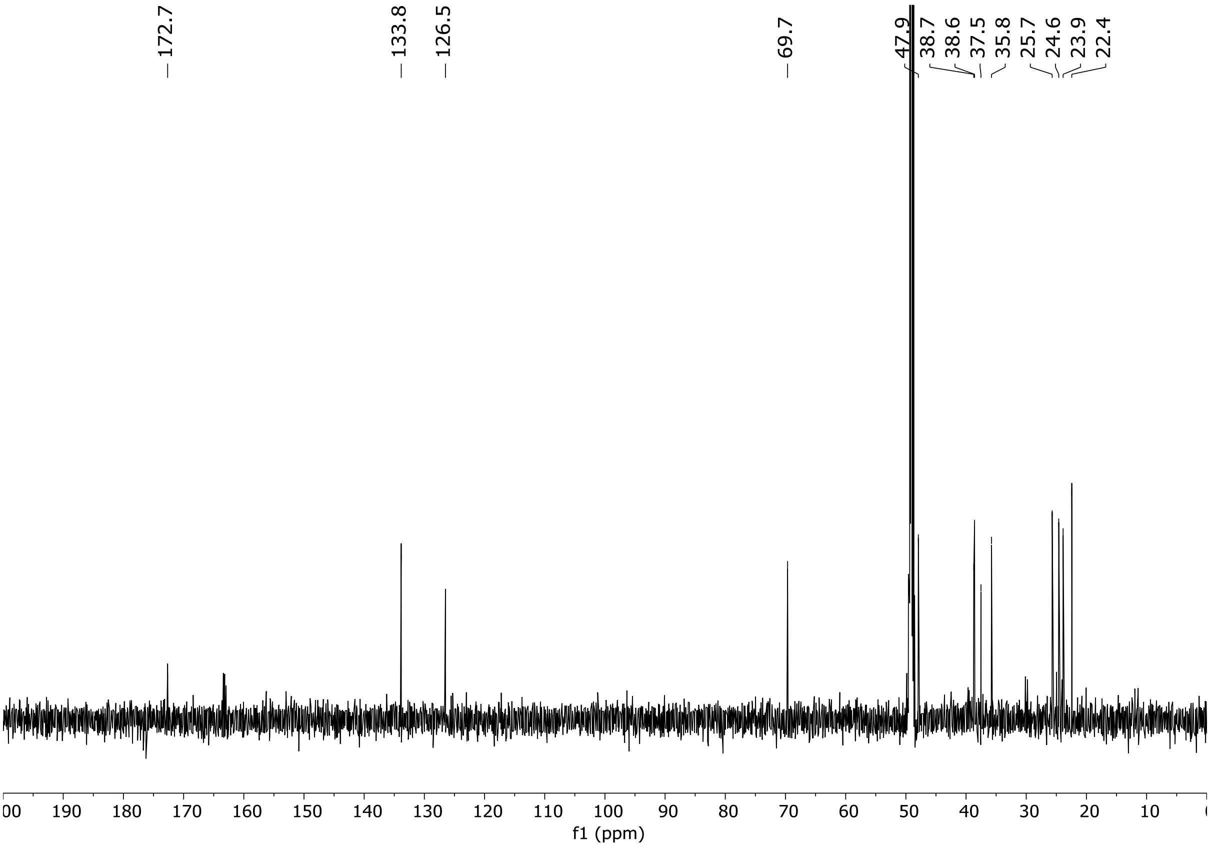

Figure S1-2. ^{13}C NMR spectrum (150 MHz) of **1** in MeOH-d_4.

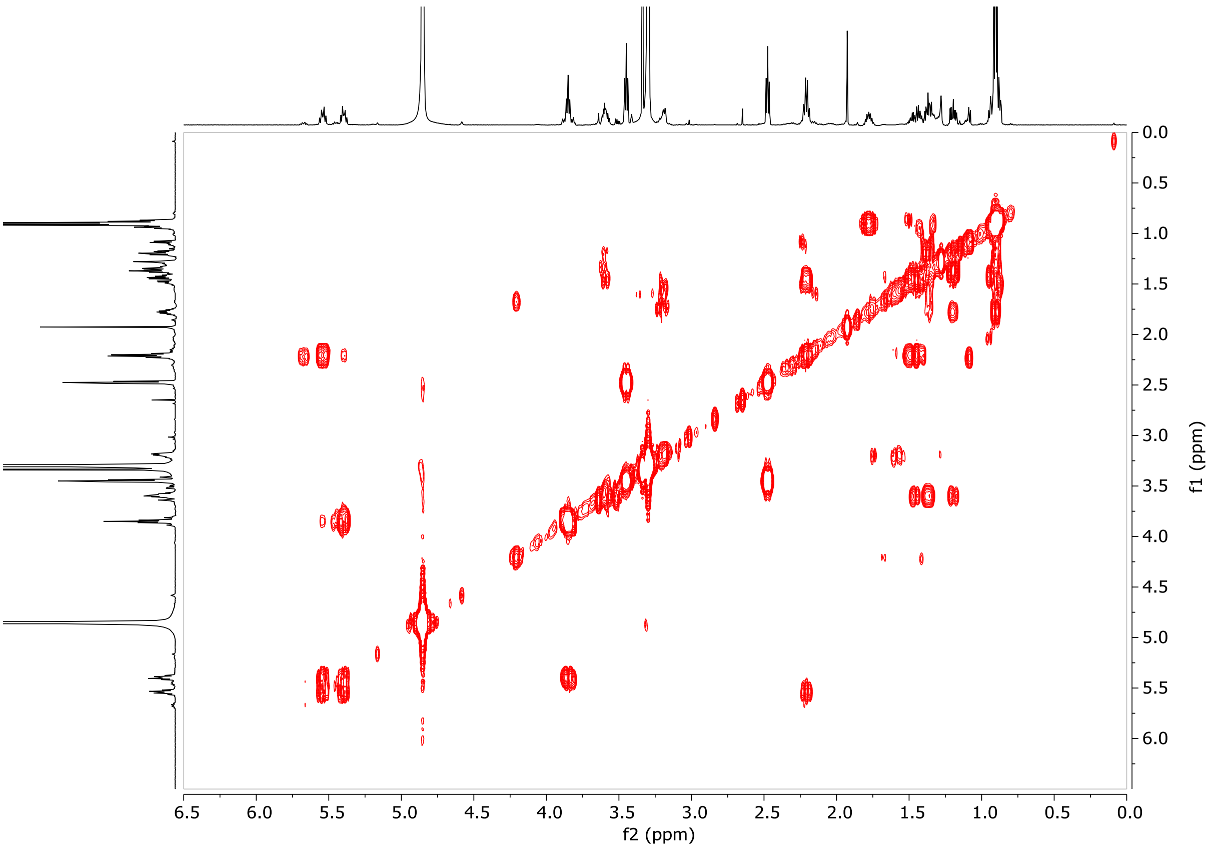

Figure S1-3. COSY spectrum (600 MHz) of **1** in MeOH-d_4.

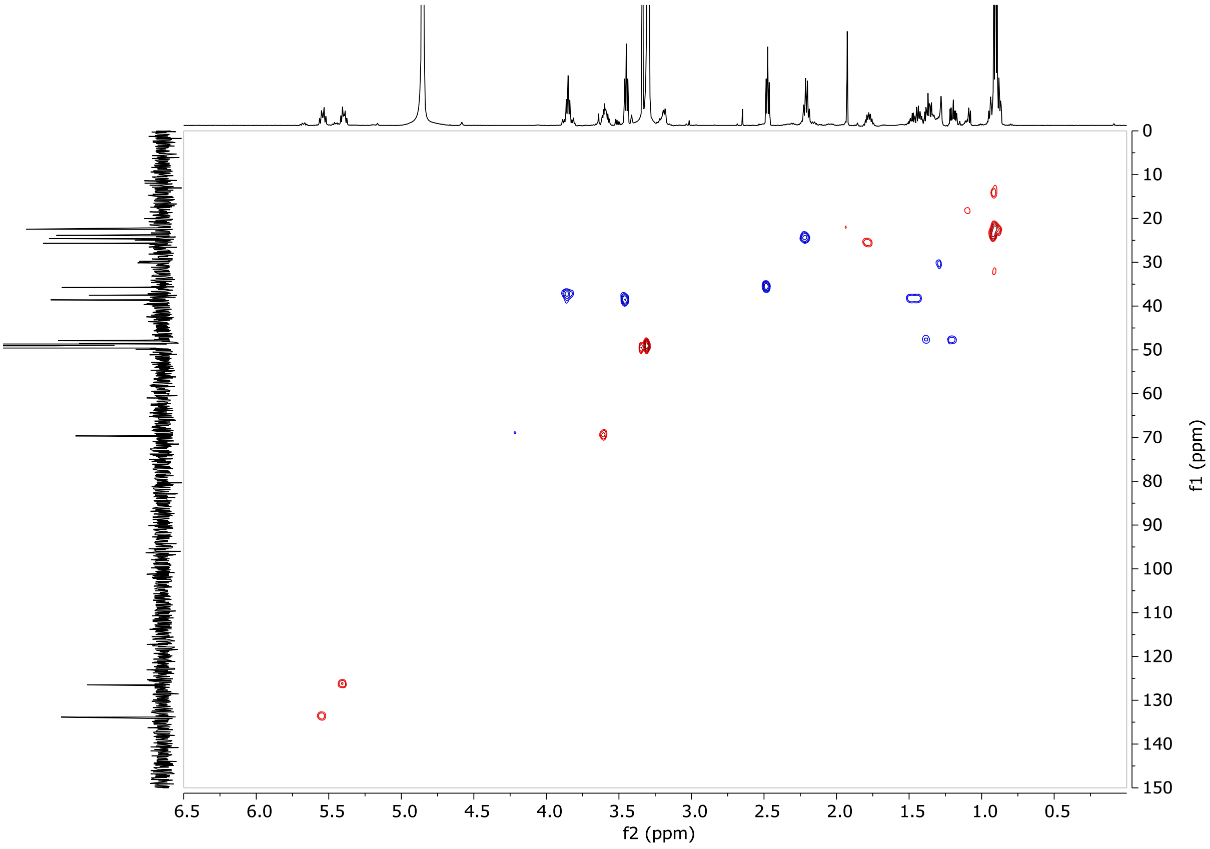

Figure S1-4. Edited HSQC spectrum (600 MHz) of **1** in MeOH-d_4.

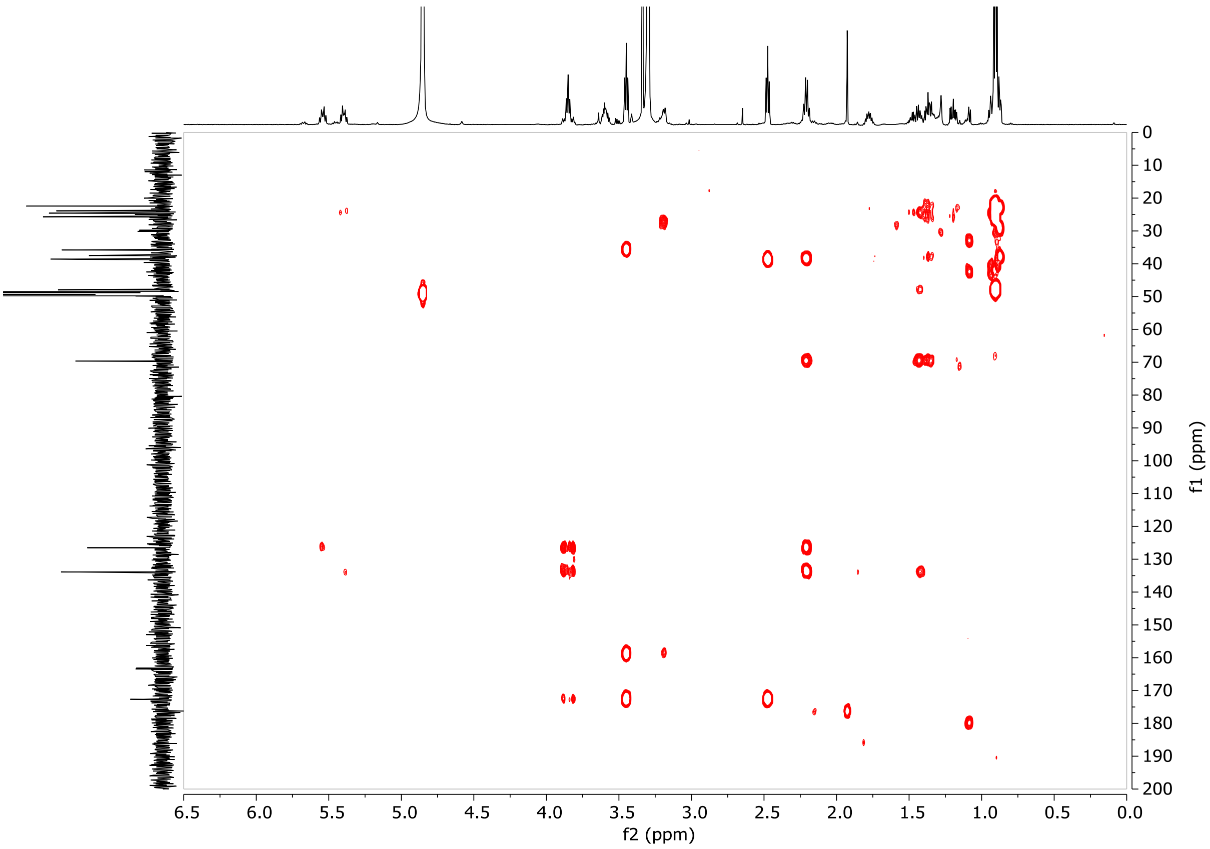

Figure S1-5. HMBC spectrum (600 MHz) of **1** in MeOH-d_4.

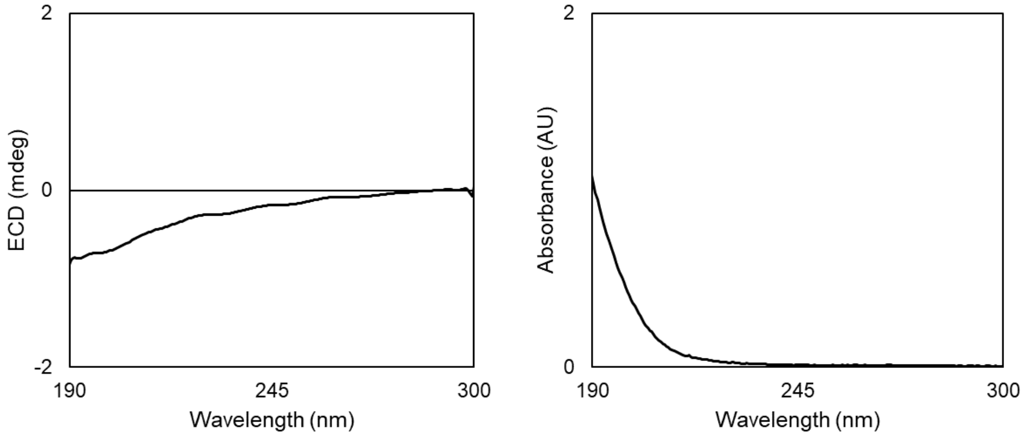

Figure S1-6. ECD and UV spectrum (c 0.25 mM) of **1** in H_2O.

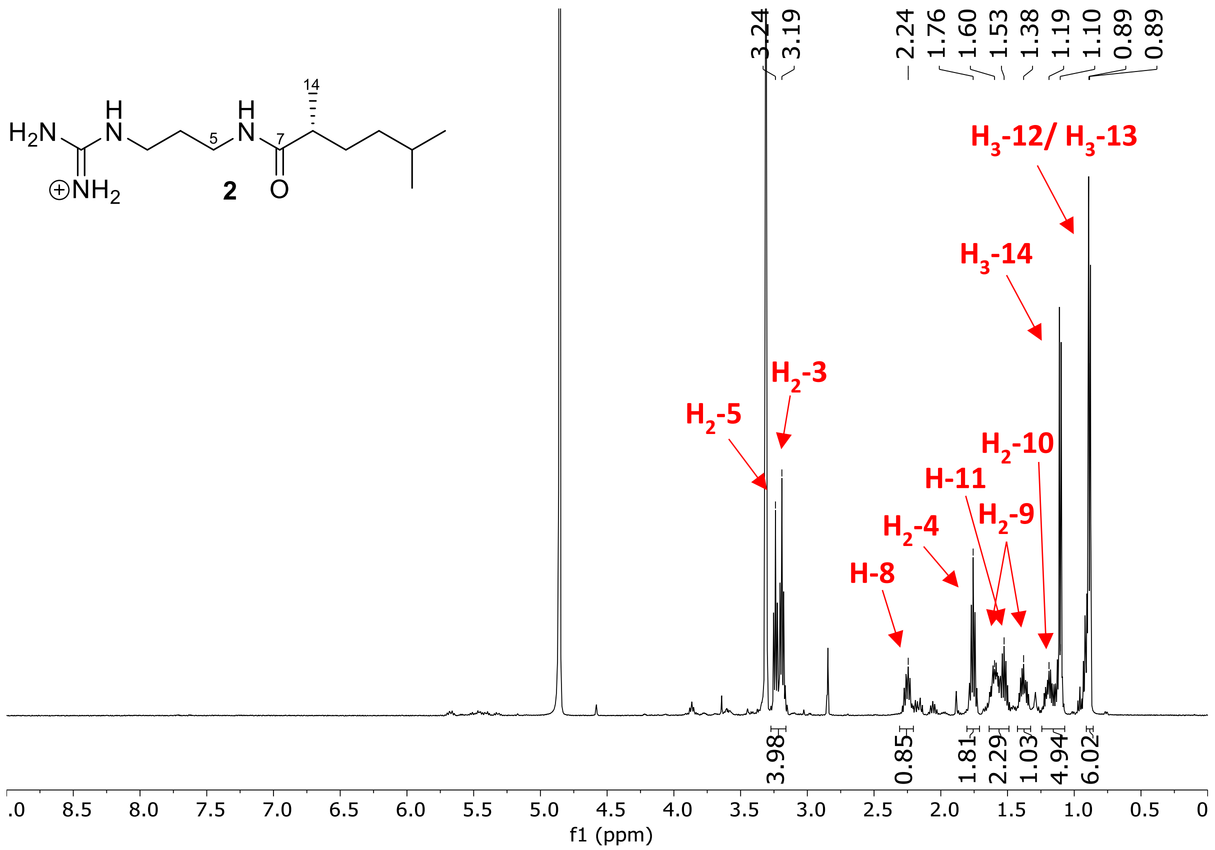

Figure S2-1. ^{1}H NMR spectrum (500 MHz) of **2** in MeOH-d_4.

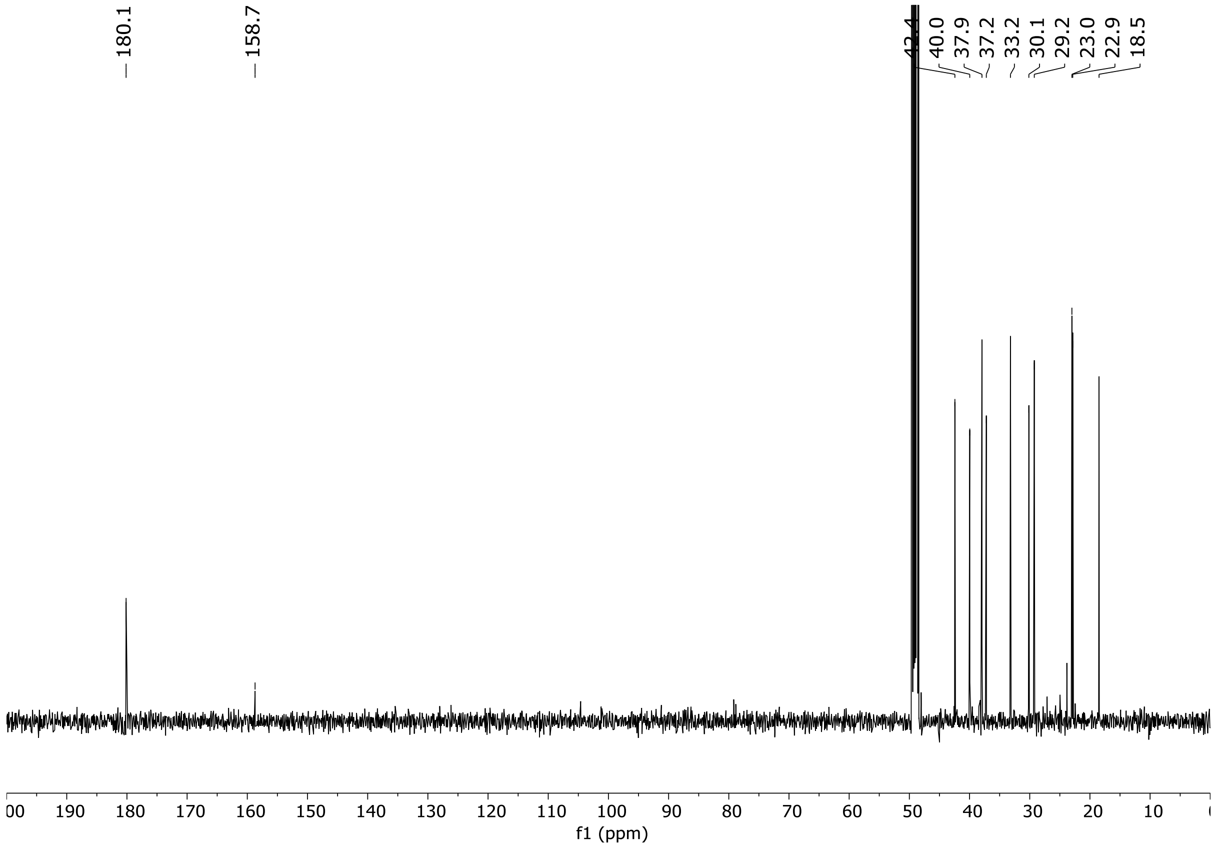

Figure S2-2. ^{13}C NMR spectrum (125 MHz) of **2** in MeOH-d_4.

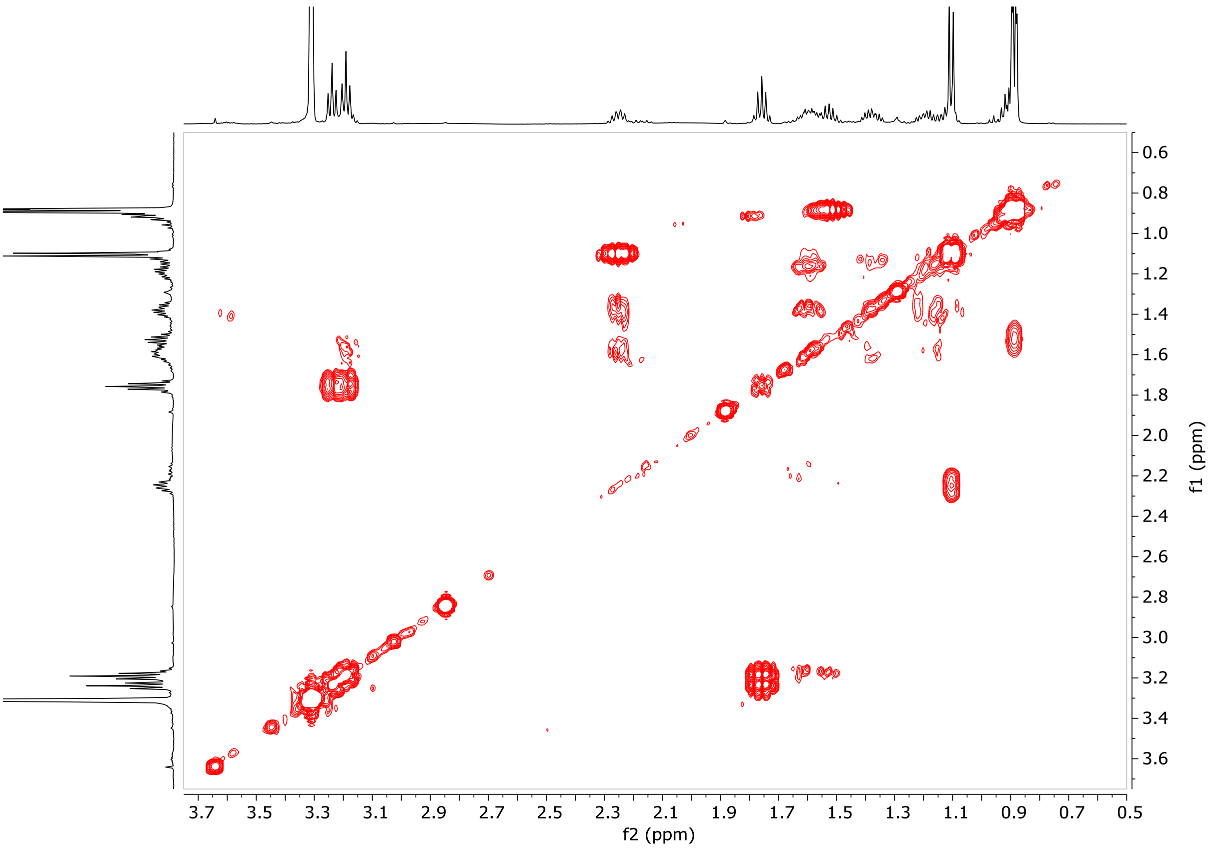

Figure S2-3. COSY spectrum (500 MHz) of **2** in MeOH-d_4.

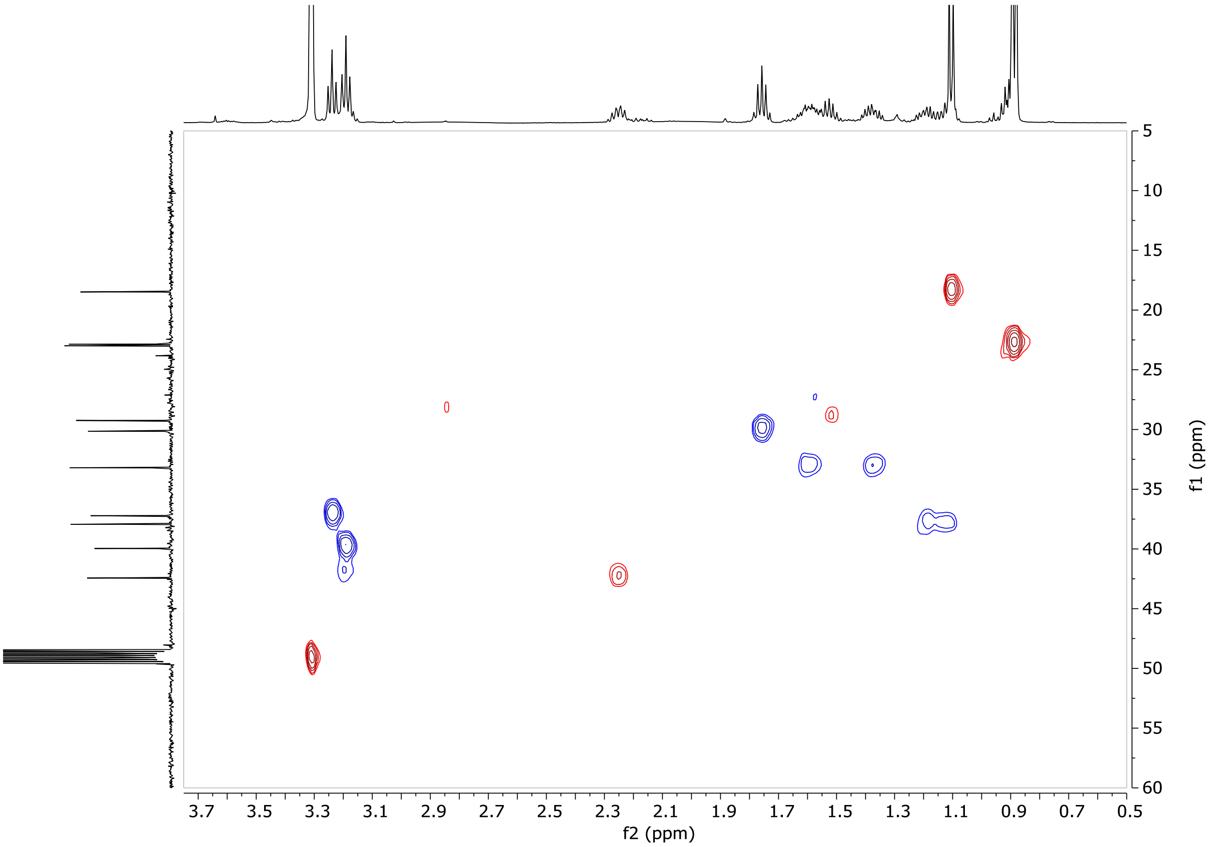

Figure S2-4. Edited HSQC spectrum (500 MHz) of **2** in MeOH-d_4.

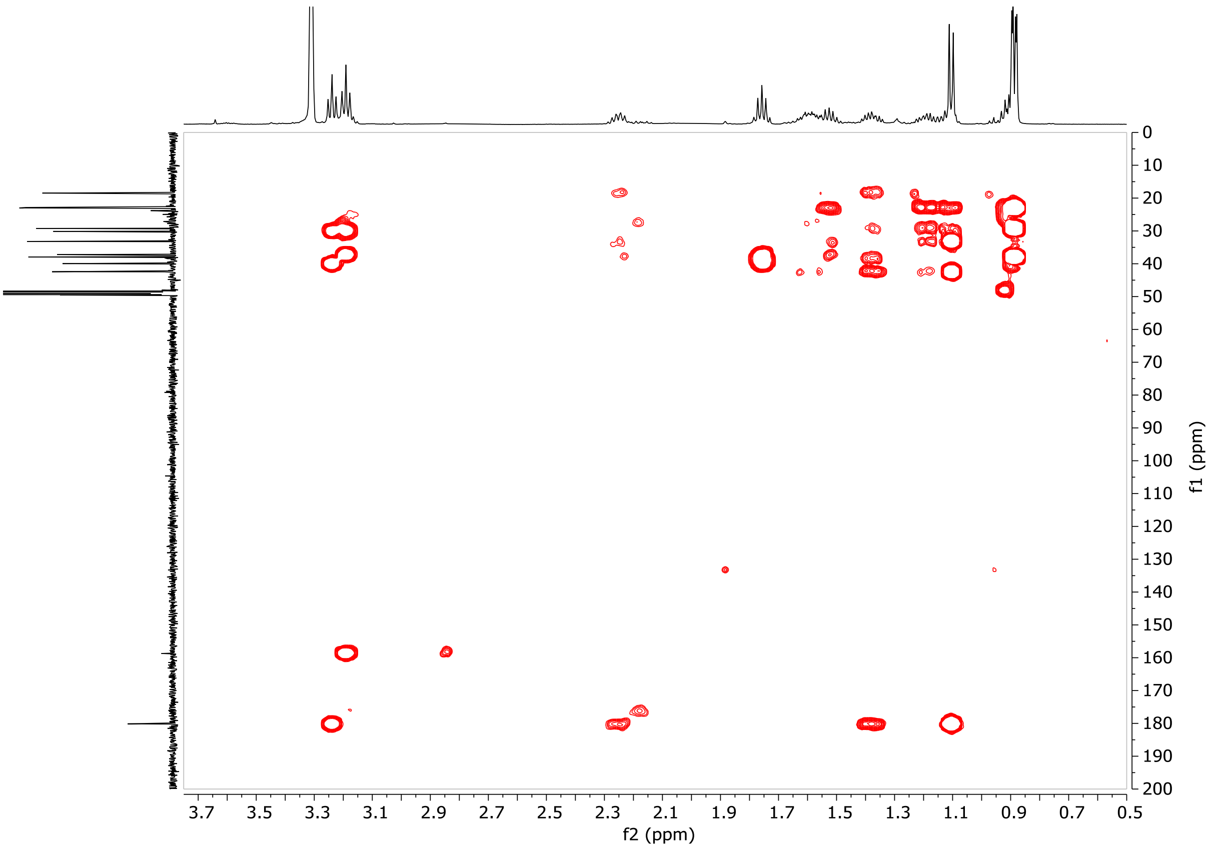

Figure S2-5. HMBC spectrum (500 MHz) of **2** in MeOH-d_4.

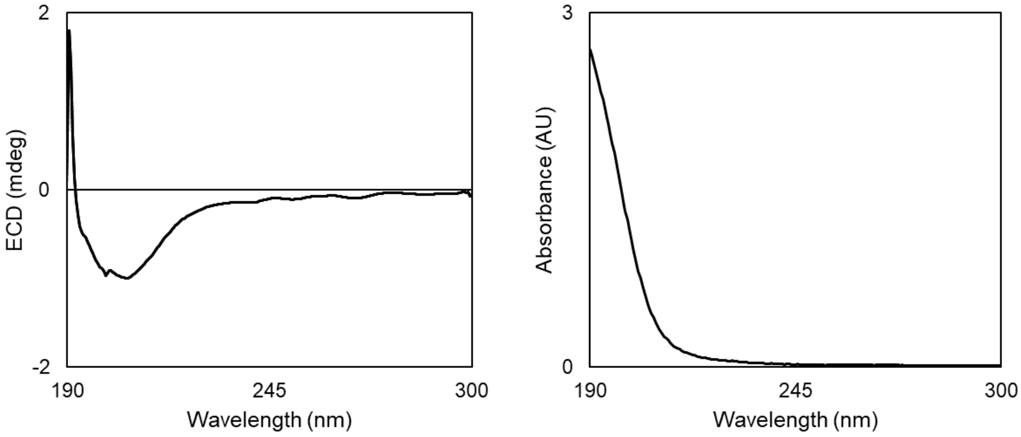

Figure S2-6. ECD and UV spectrum (c 0.14 mM) of **2** in H_2O.

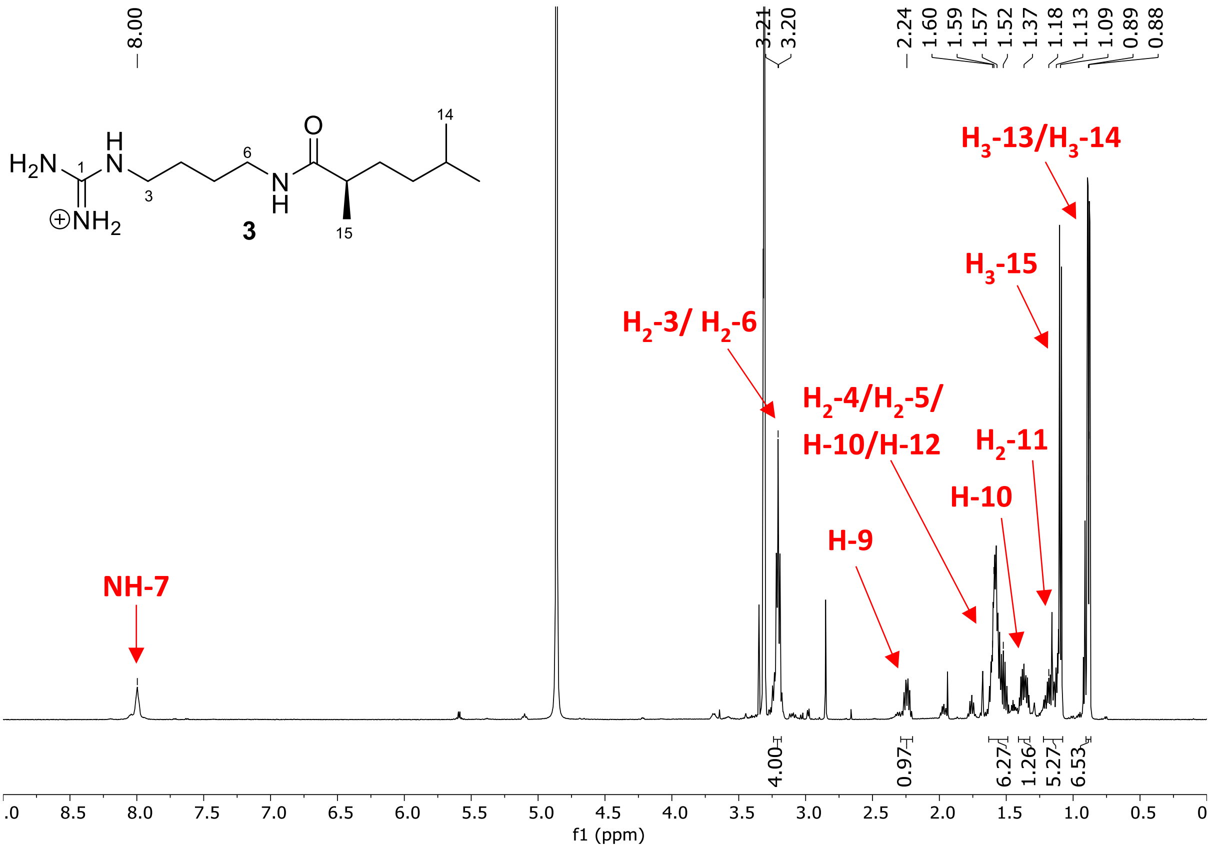

Figure S3-1. ^{1}H NMR spectrum (500 MHz) of **3** in MeOH-d_4.

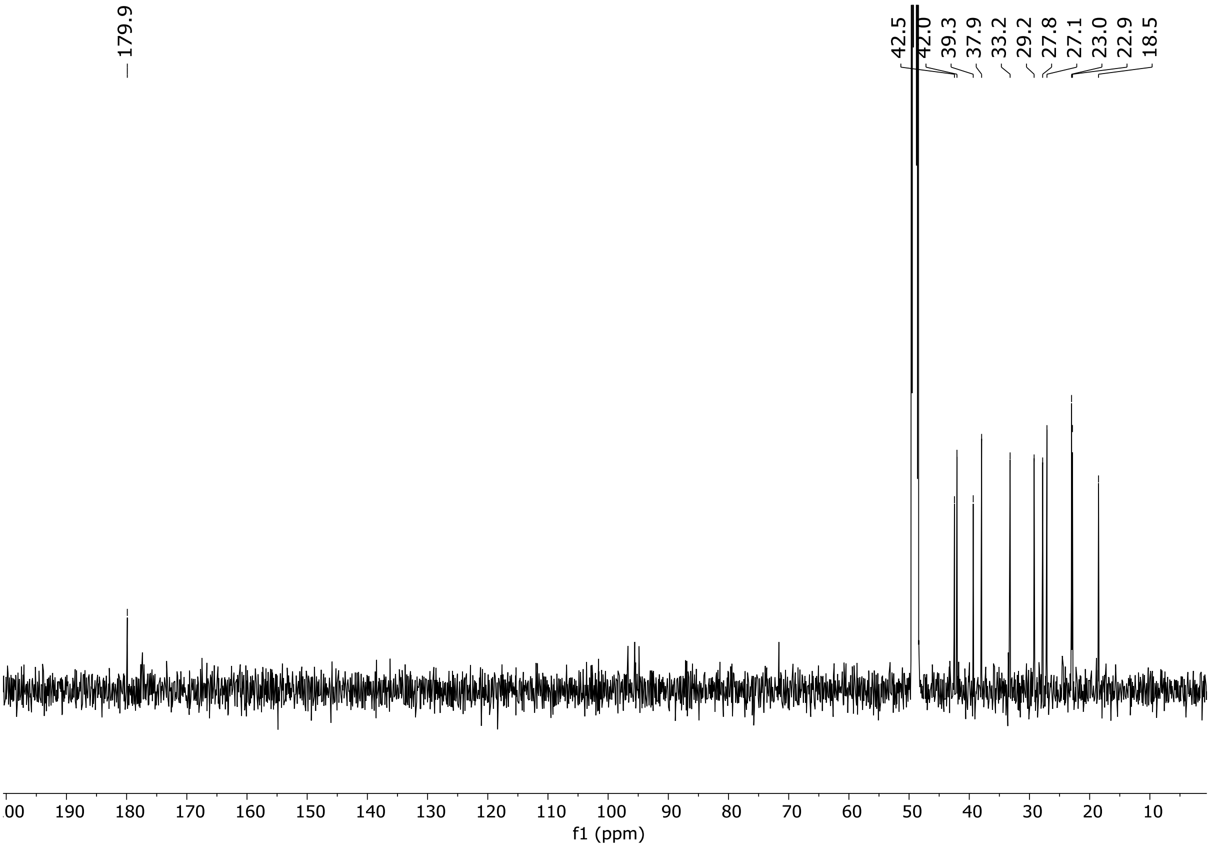

Figure S3-2. ^{13}C NMR spectrum (125 MHz) of **3** in MeOH-d_4.

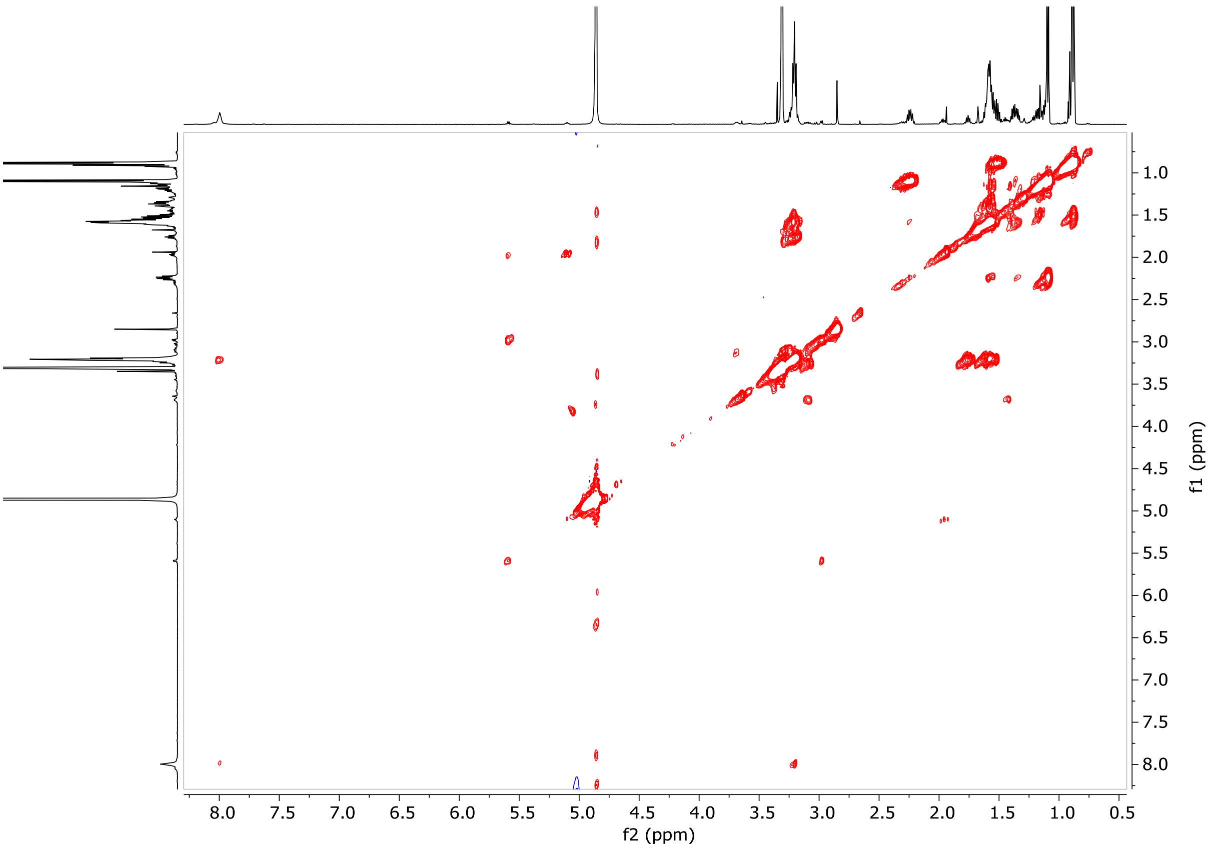

Figure S3-3. COSY spectrum (500 MHz) of **3** in MeOH-d_4.

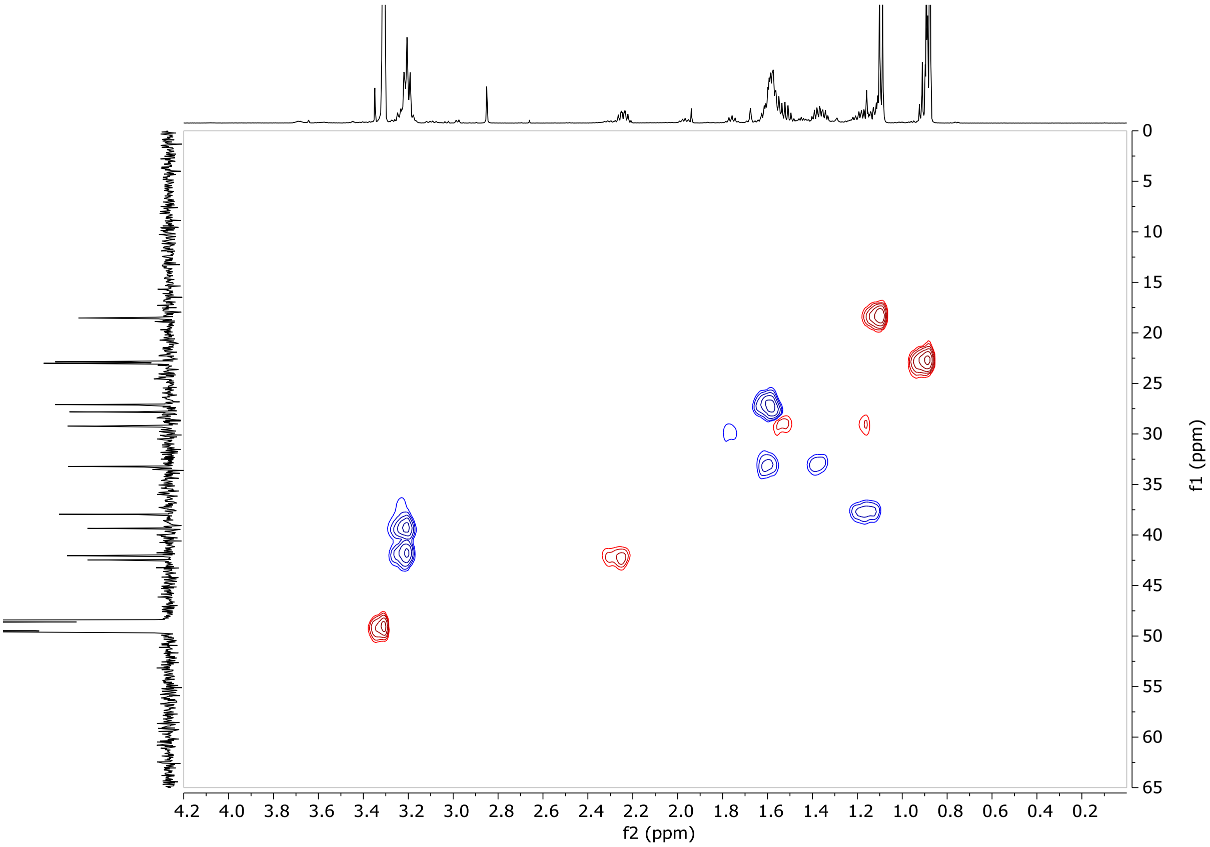

Figure S3-4. Edited HSQC spectrum (500 MHz) of **3** in MeOH-d_4.

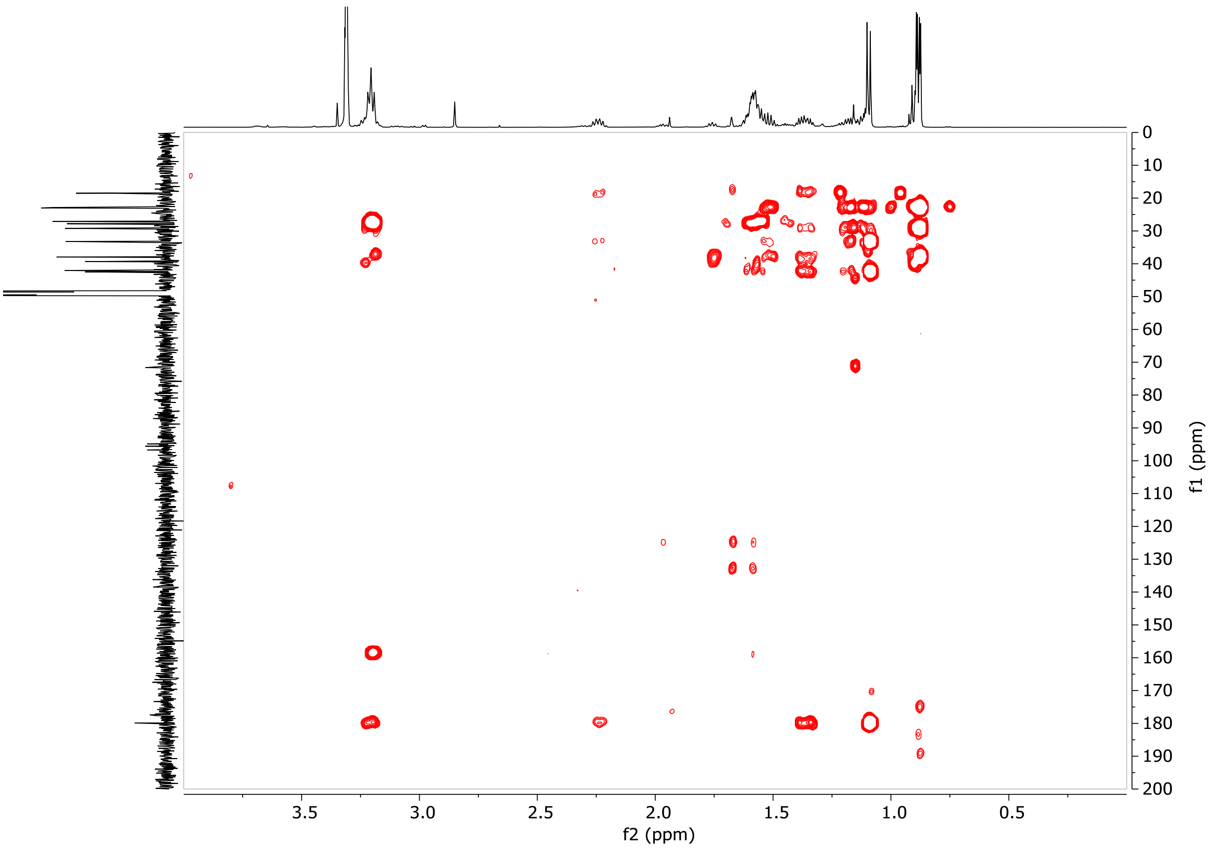

Figure S3-5. HMBC spectrum (500 MHz) of **3** in MeOH-d_4.

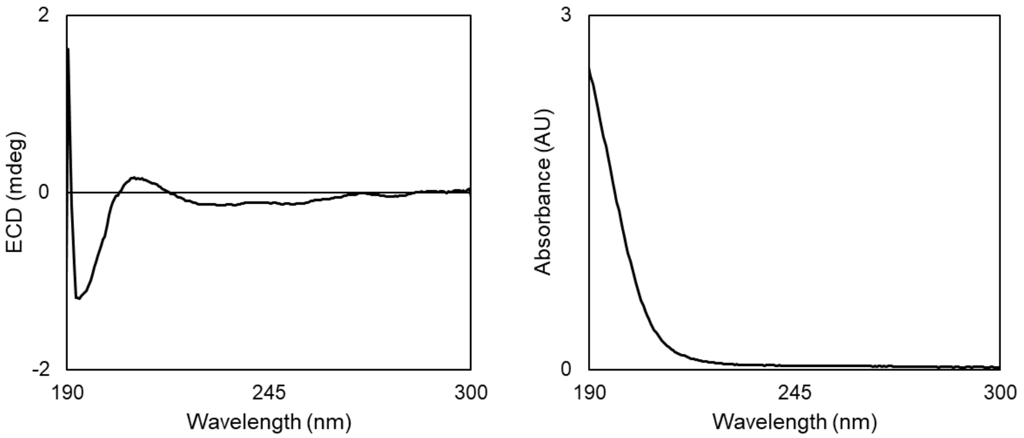

Figure S3-6. ECD and UV spectrum (c 0.13 mM) of **3** in H_2O.

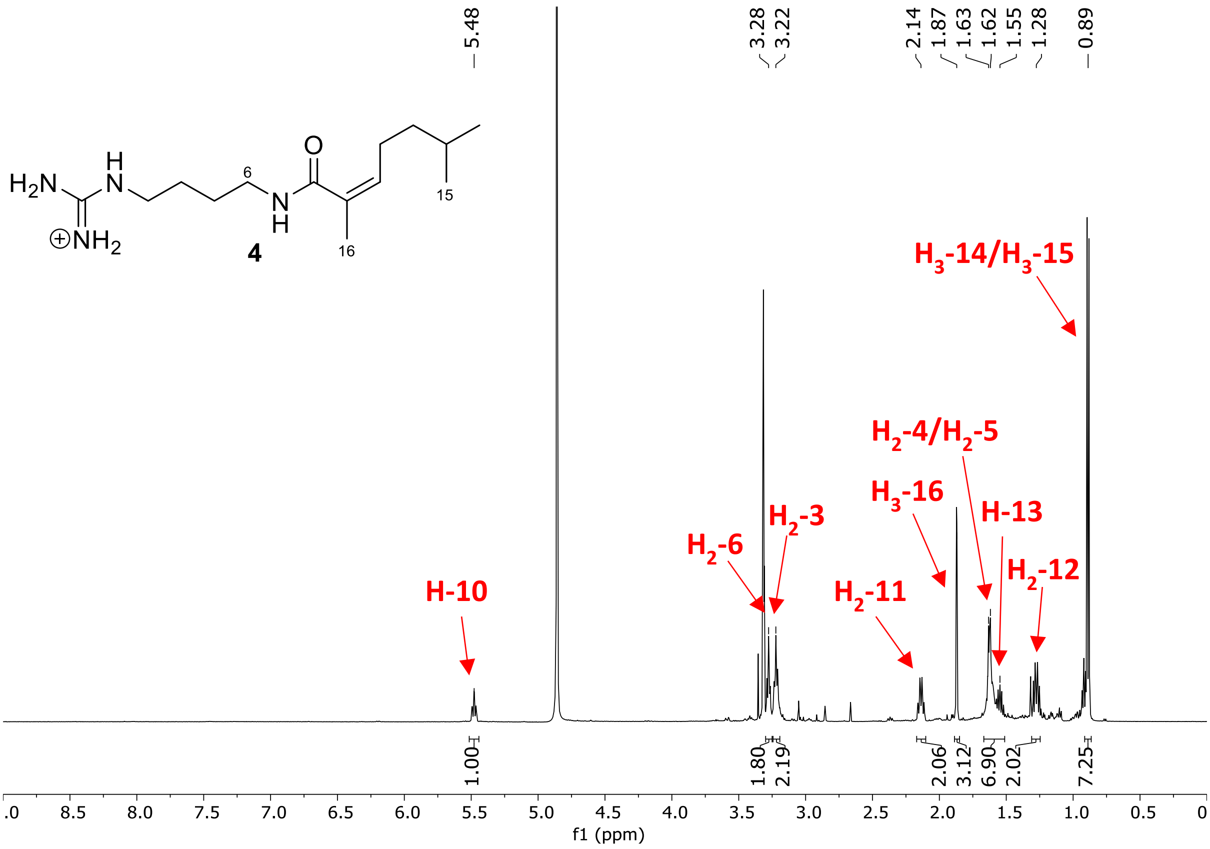

Figure S4-1. ^{1}H NMR spectrum (500 MHz) of **4** in MeOH-d_4.

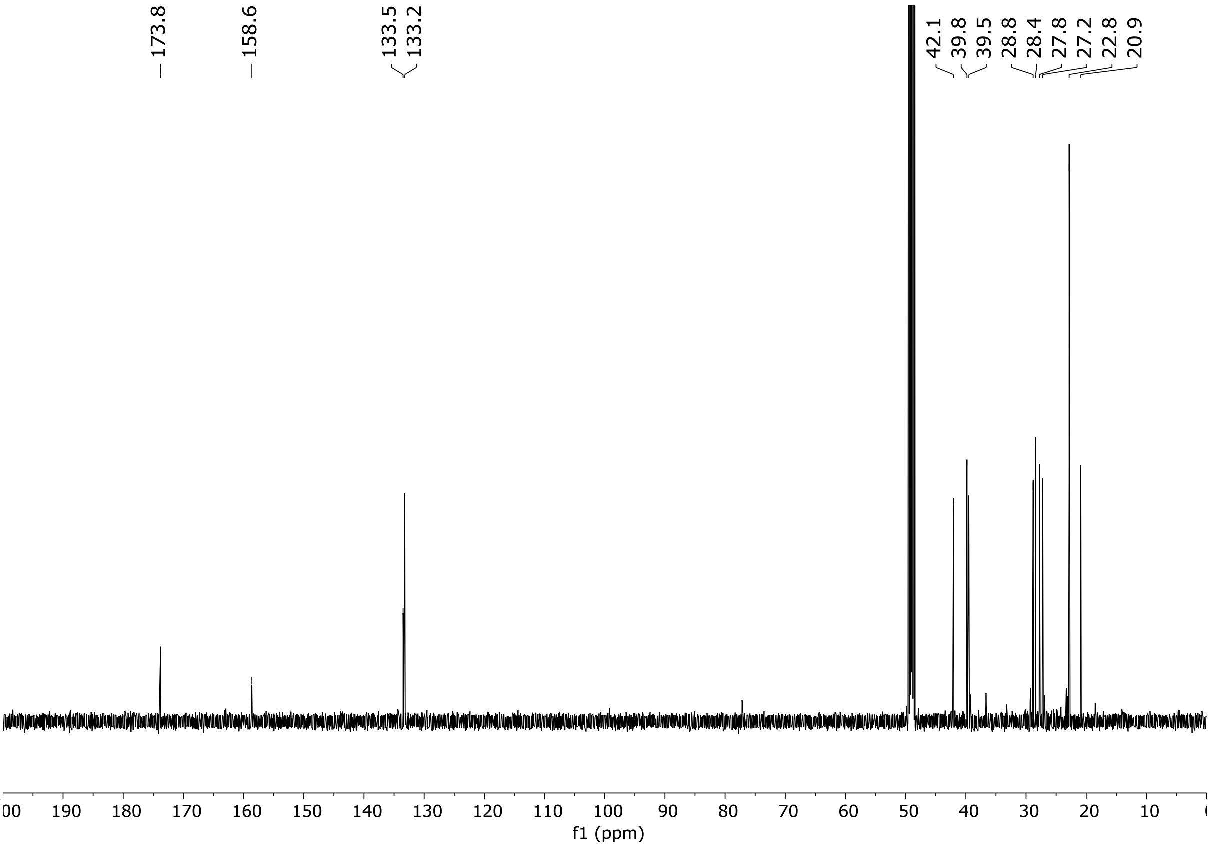

Figure S4-2. ^{13}C NMR spectrum (125 MHz) of **4** in MeOH-d_4.

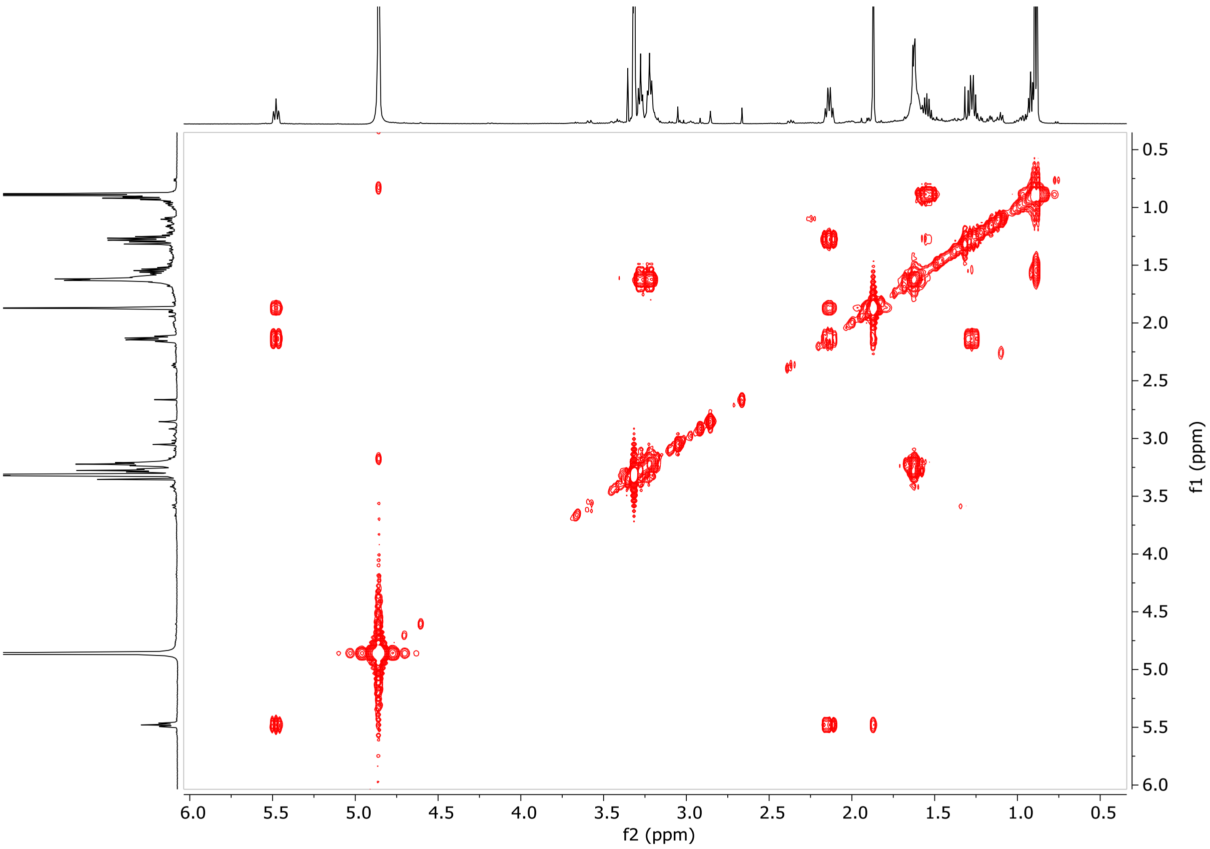

Figure S4-3. COSY spectrum (500 MHz) of **4** in MeOH-d_4.

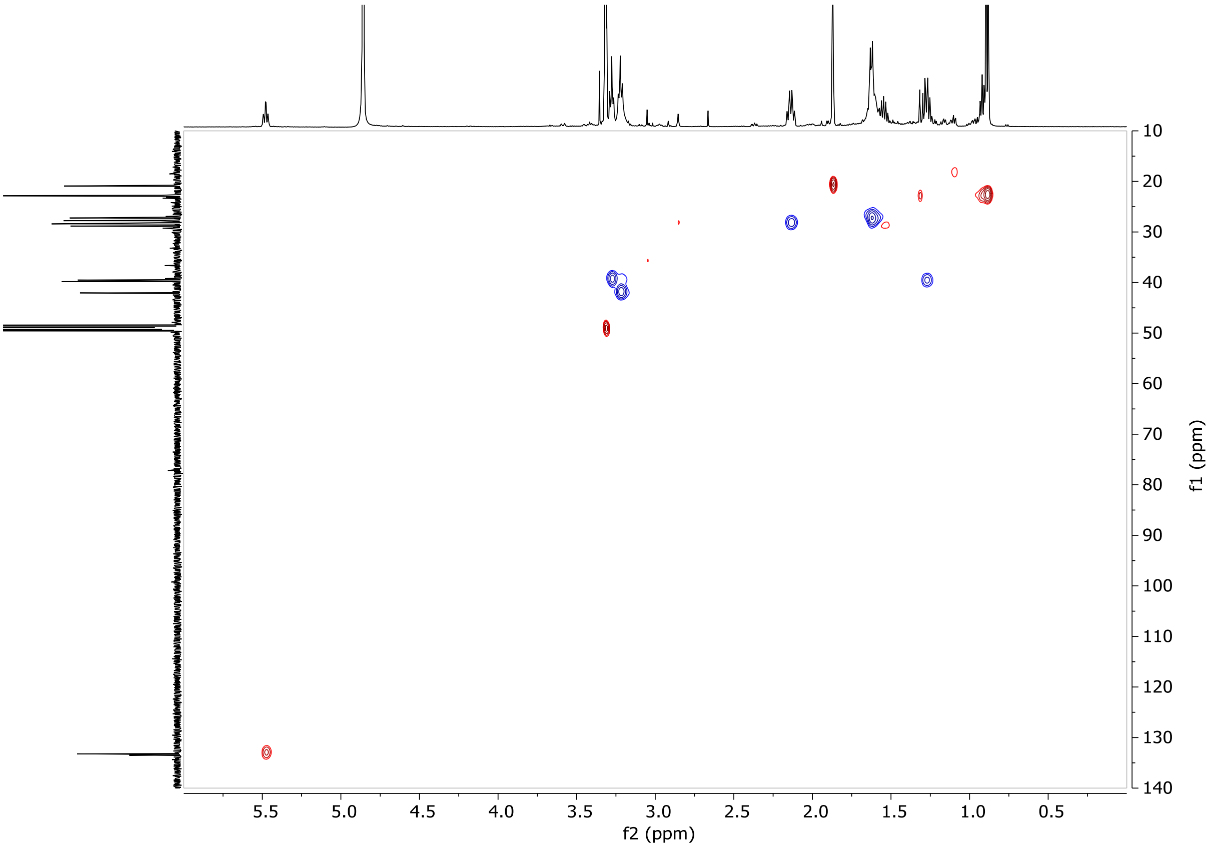

Figure S4-4. Edited HSQC spectrum (500 MHz) of **4** in MeOH-d_4.

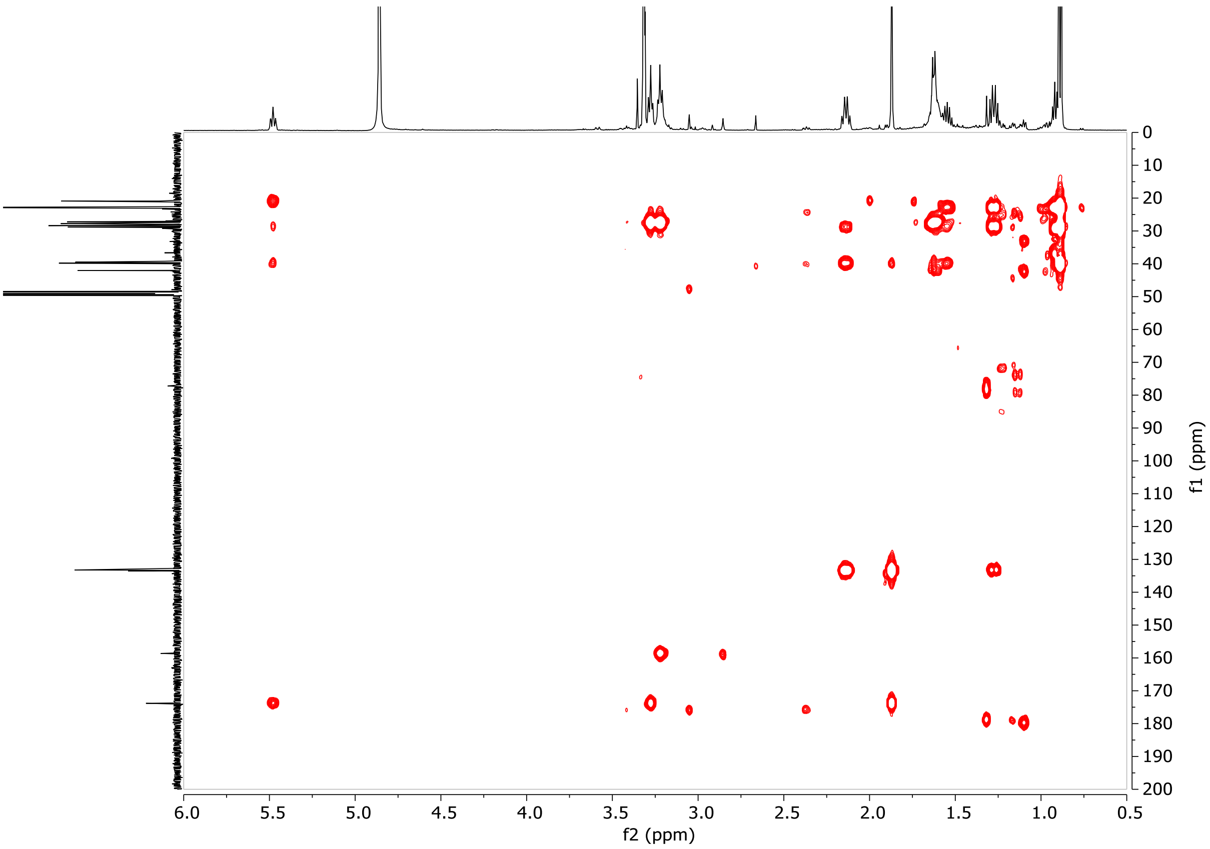

Figure S4-5. HMBC spectrum (500 MHz) of **4** in MeOH-d_4.

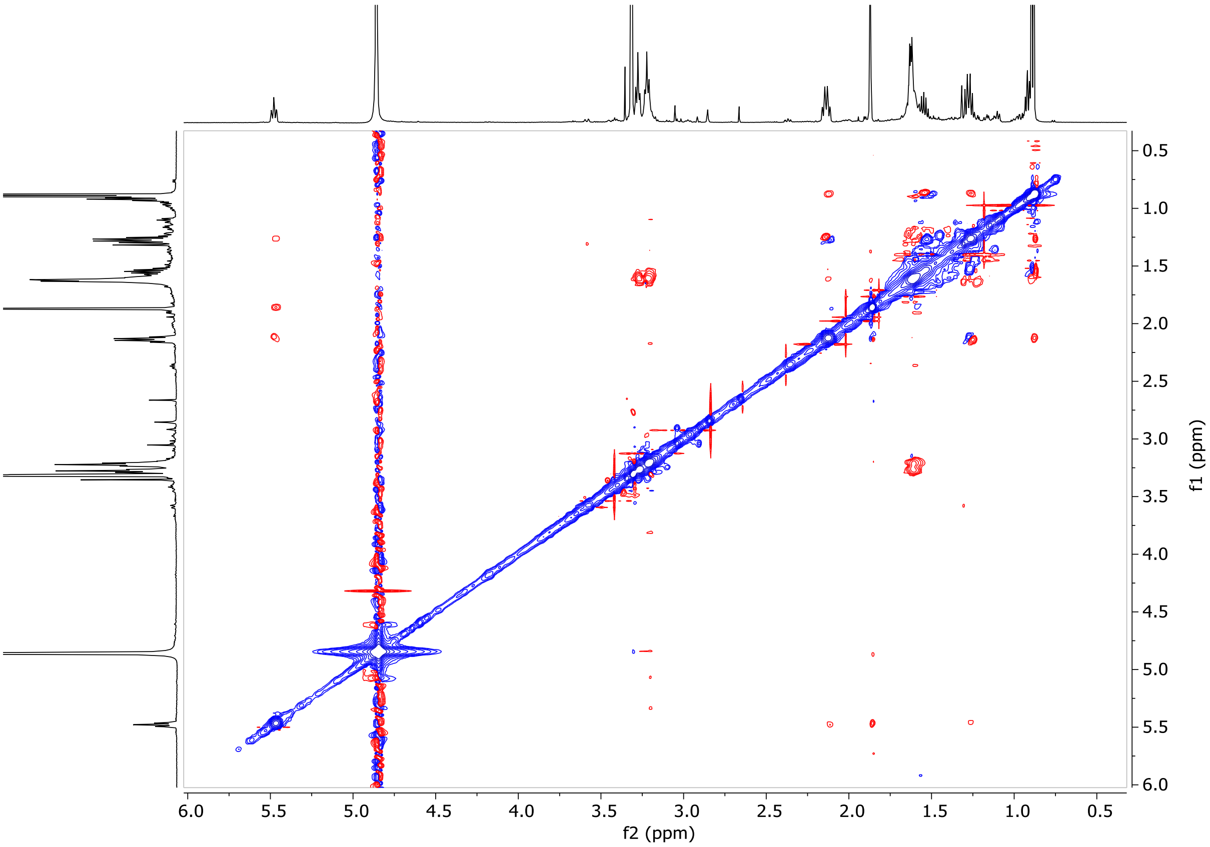

Figure S4-6. ROESY spectrum (500 MHz) of **4** in MeOH-d_4.

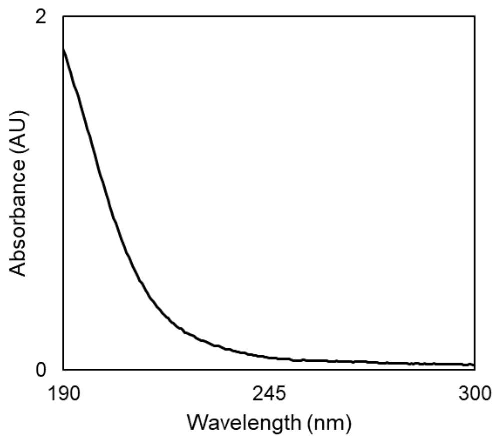

Figure S4-7. UV spectrum (c 1.90 mM) of **4** in H_2O.

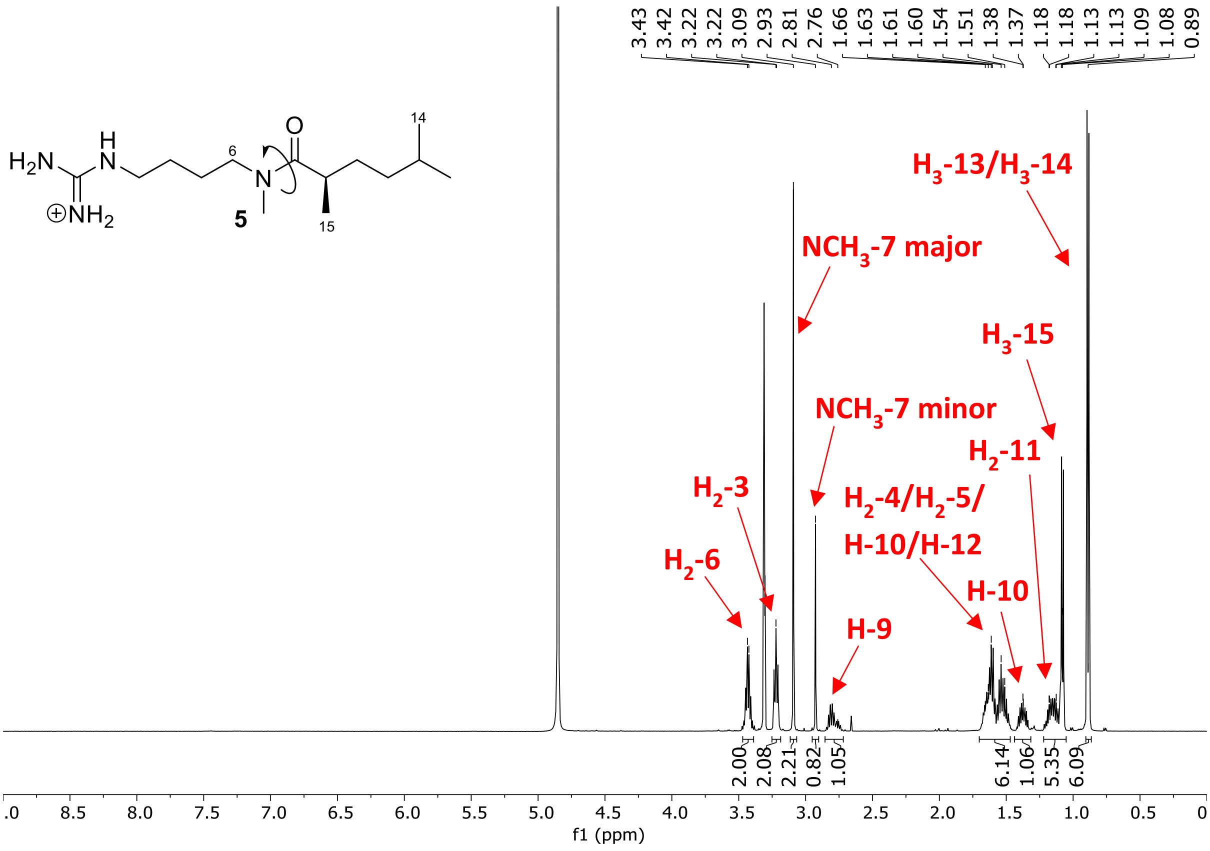

Figure S5-1. ^{1}H NMR spectrum (500 MHz) of **5** in MeOH-d_4.

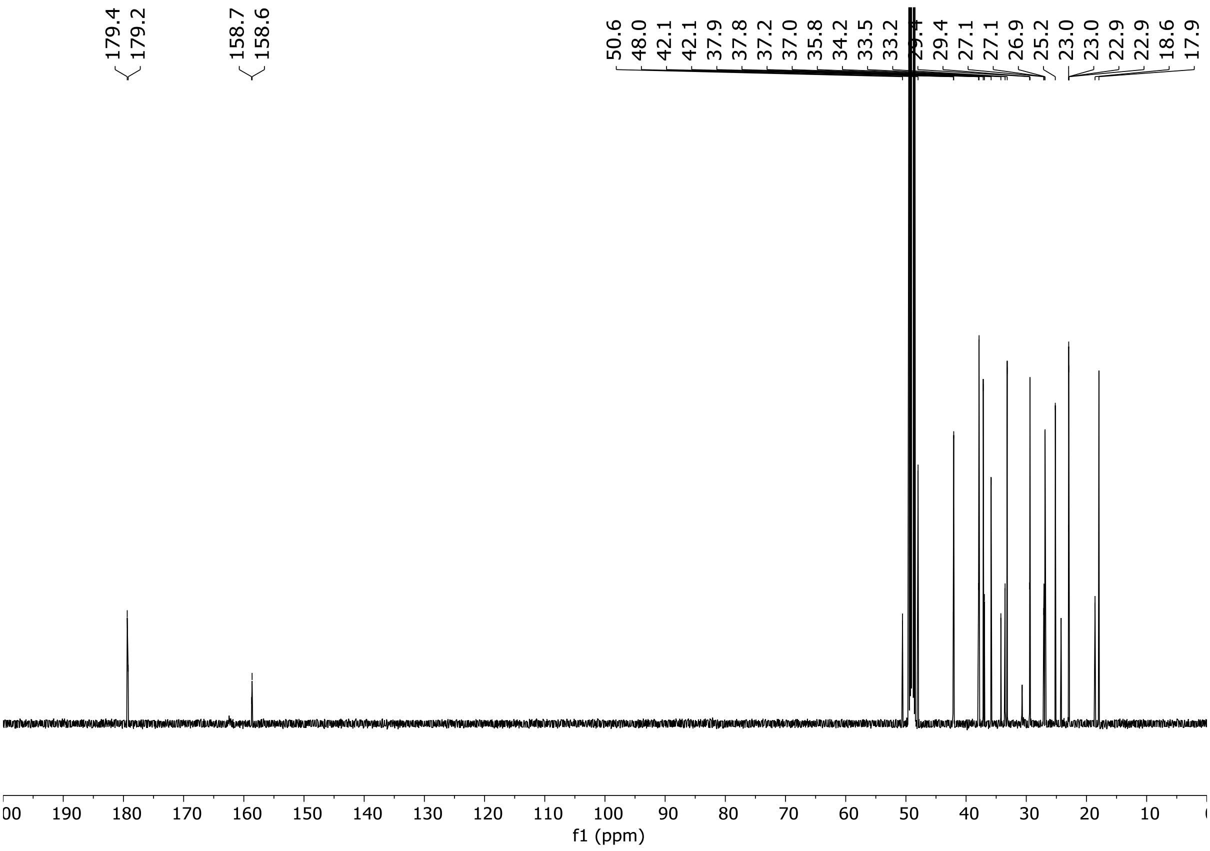

Figure S5-2. ^{13}C NMR spectrum (125 MHz) of **5** in MeOH-d_4.

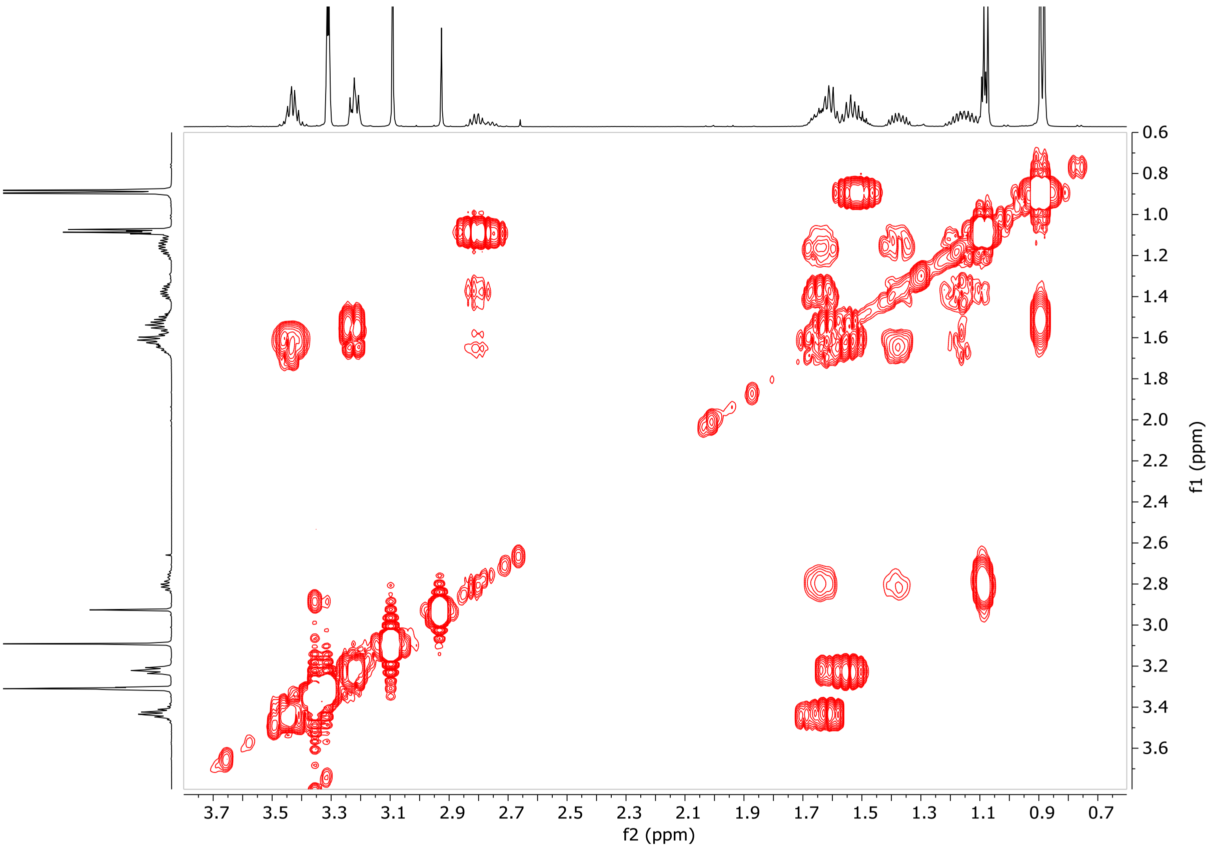

Figure S5-3. COSY spectrum (500 MHz) of **5** in MeOH-d_4.

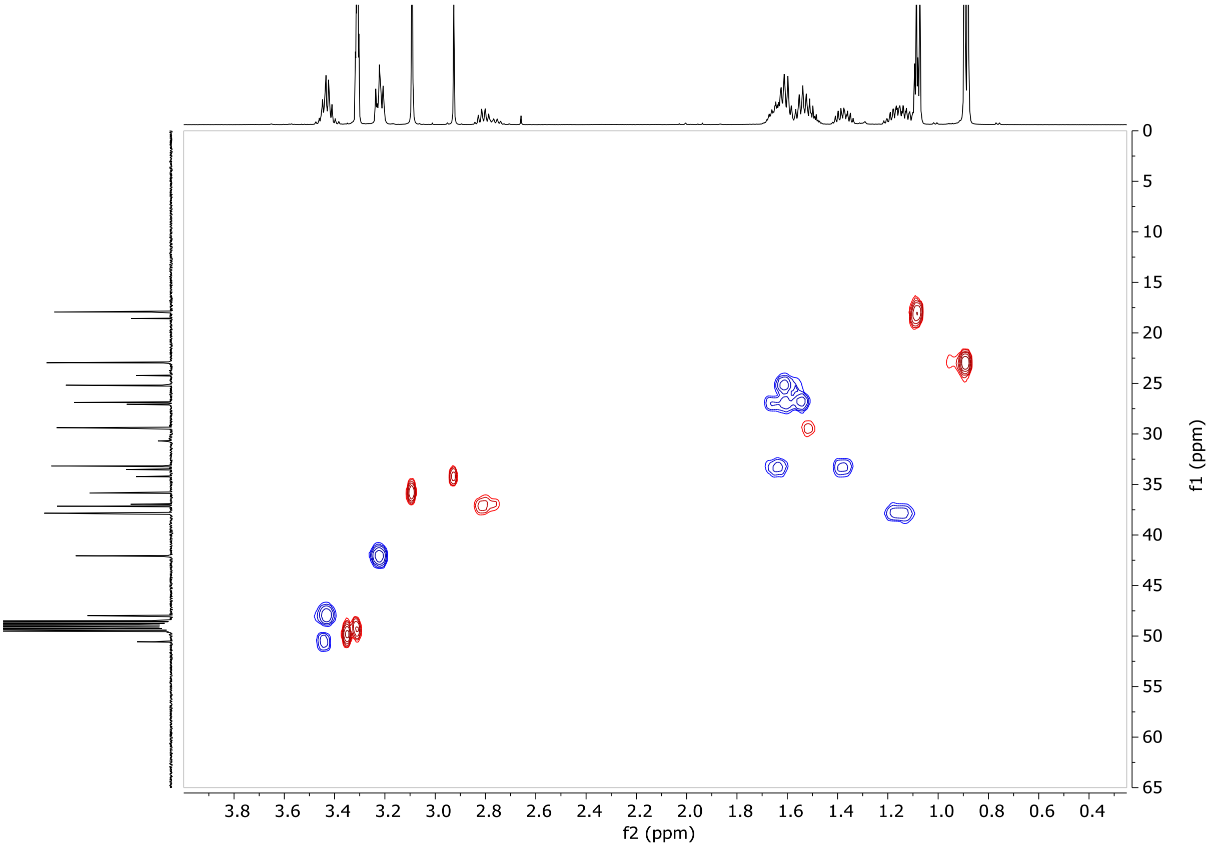

Figure S5-4. Edited HSQC spectrum (500 MHz) of **5** in MeOH-d_4.

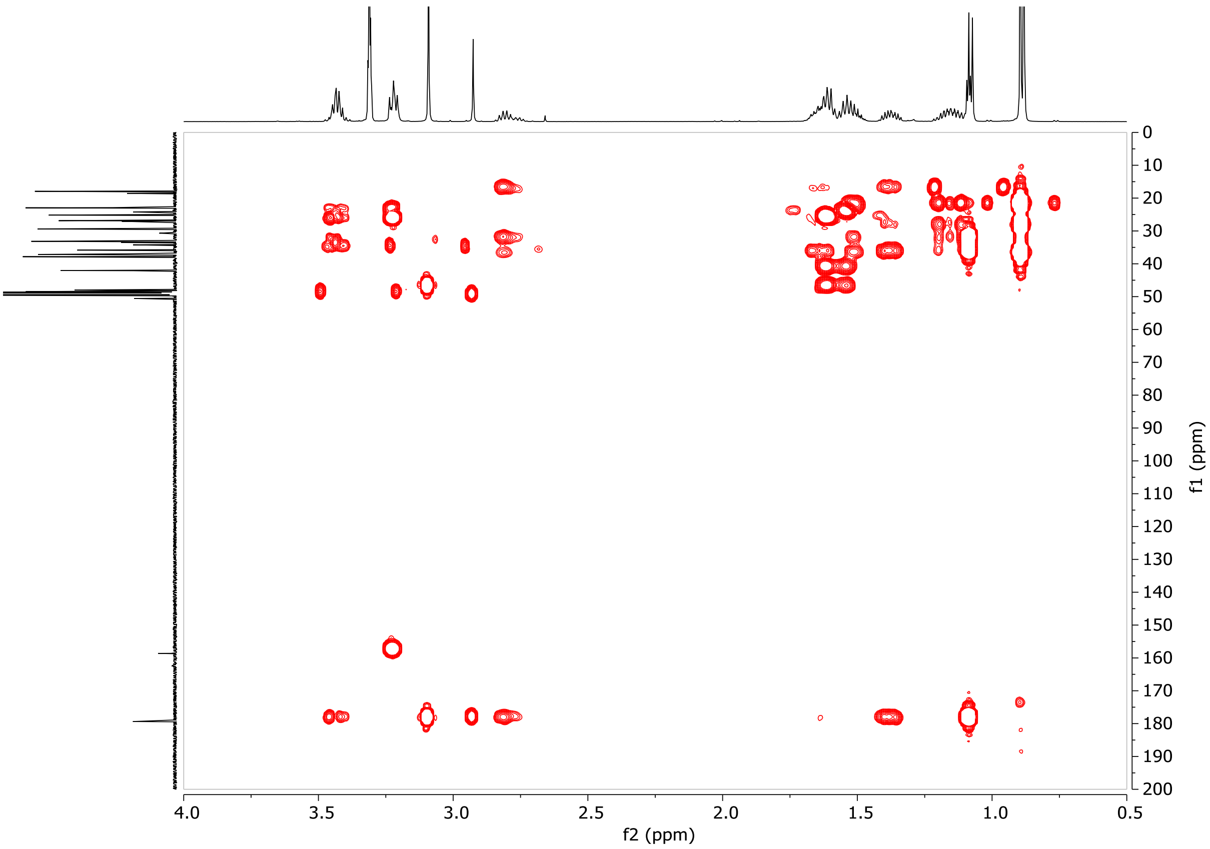

Figure S5-5. HMBC spectrum (500 MHz) of **5** in MeOH-d_4.

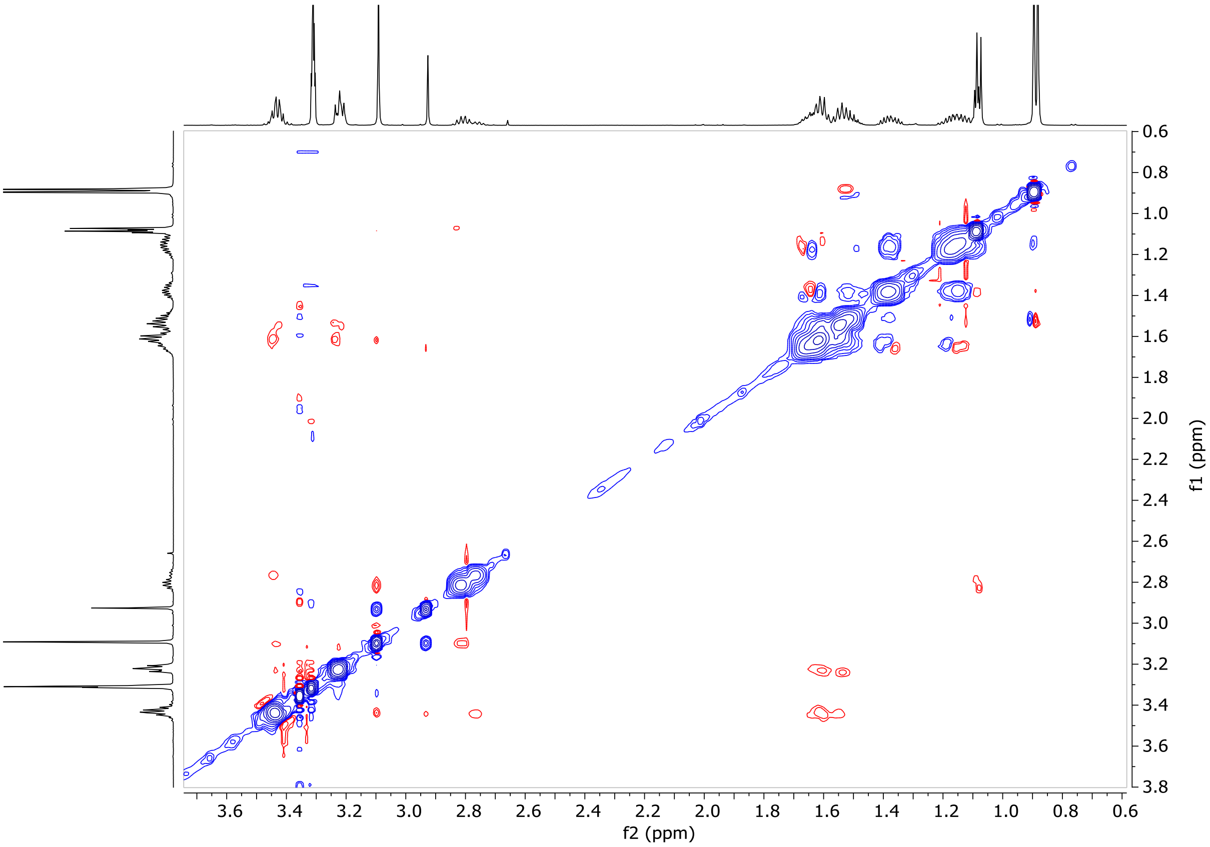

Figure S5-6. ROESY spectrum (500 MHz) of **5** in MeOH-d_4.

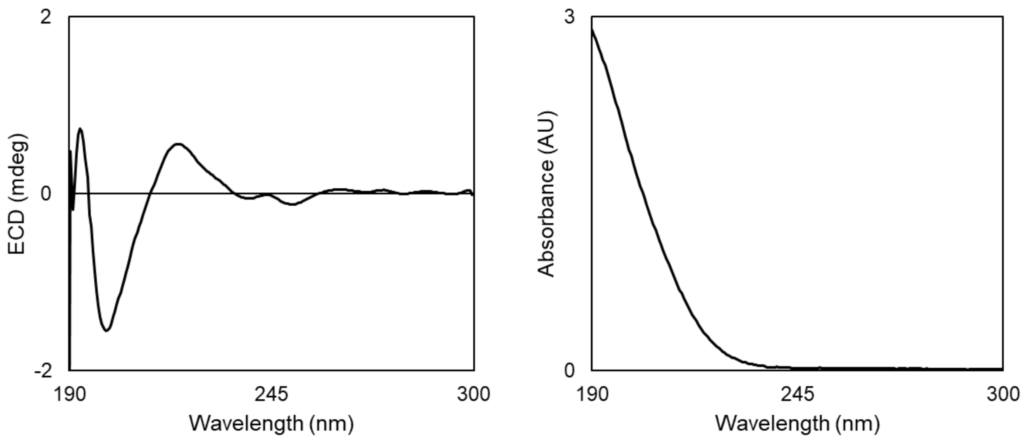

Figure S5-7. ECD and UV spectrum (c 1.08 mM) of **5** in H_2O.

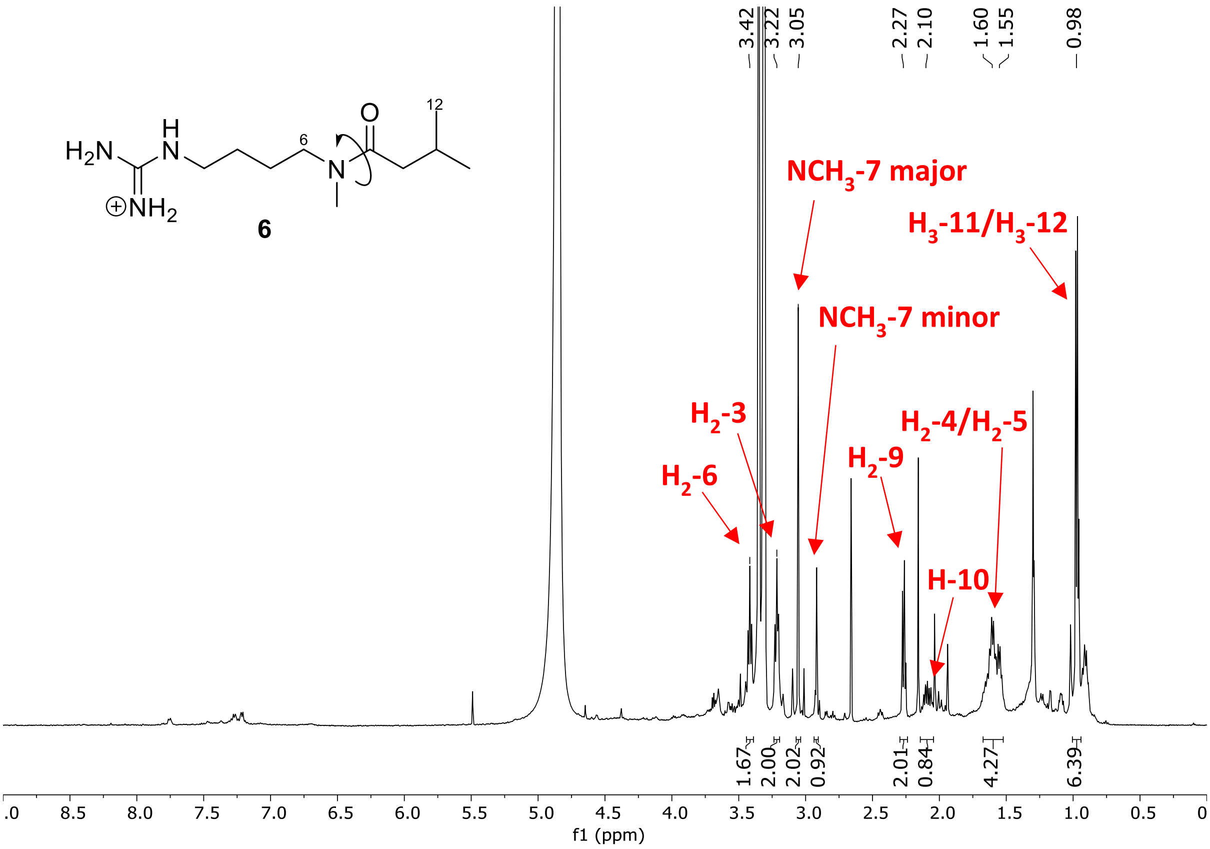

Figure S6-1. ^{1}H NMR spectrum (600 MHz) of **6** in MeOH-d_4.

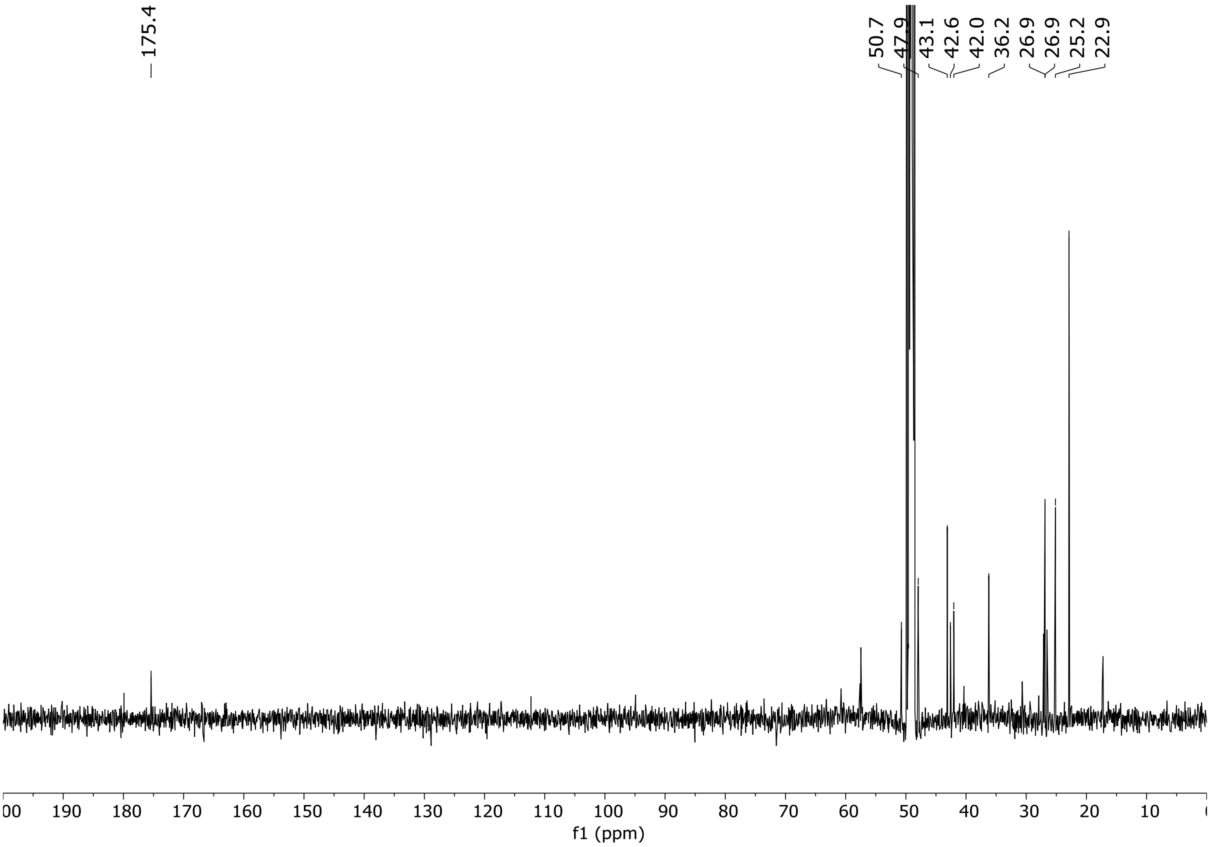

Figure S6-2. ^{13}C NMR spectrum (150 MHz) of **6** in MeOH-d_4.

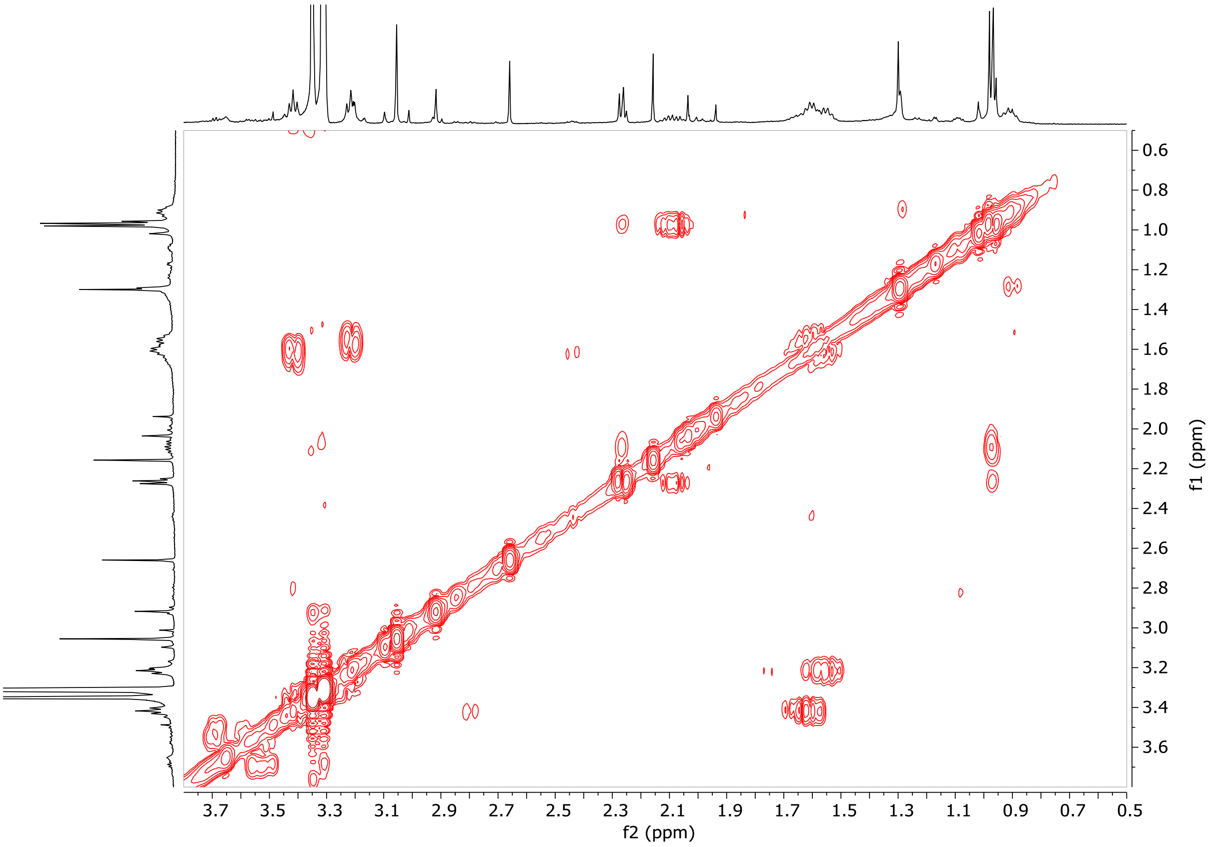

Figure S6-3. COSY spectrum (600 MHz) of **6** in MeOH-*d*4.

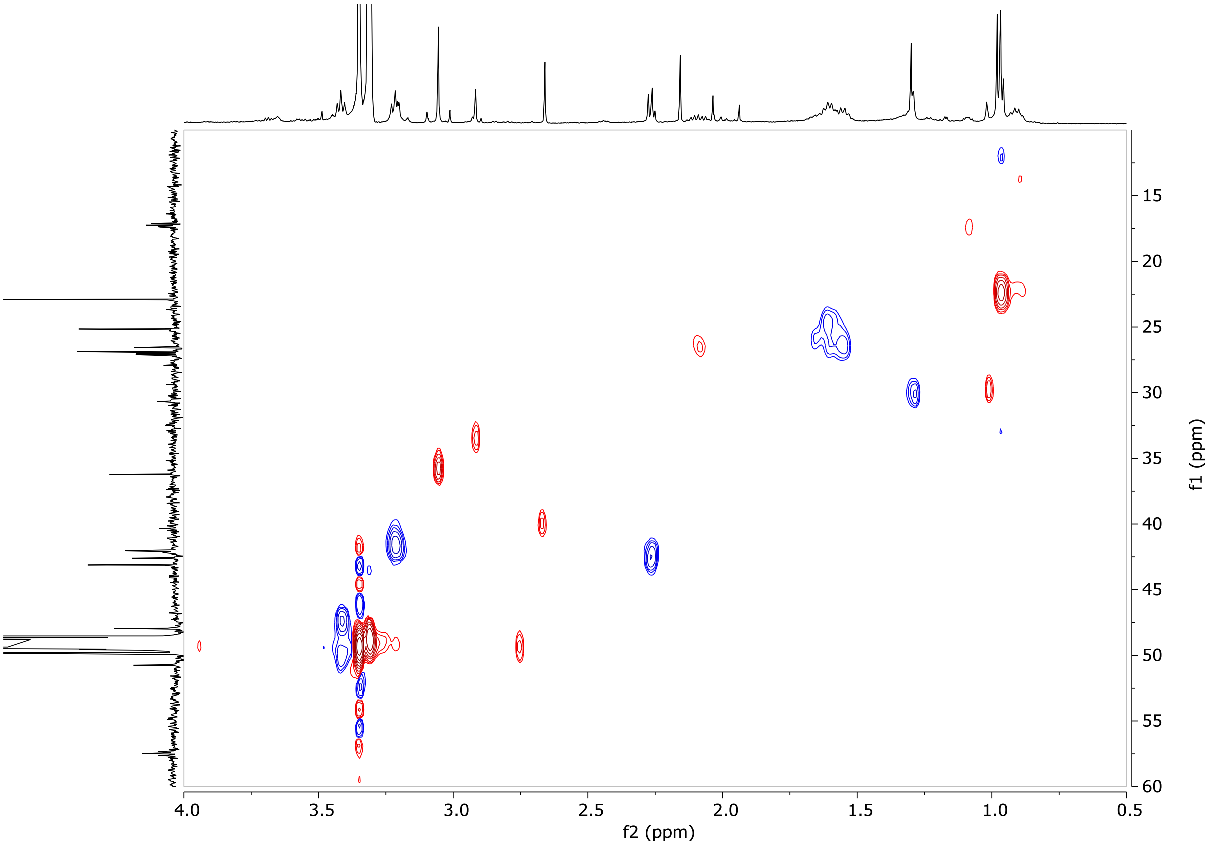

Figure S6-4. Edited HSQC spectrum (600 MHz) of **6** in MeOH-*d*4.

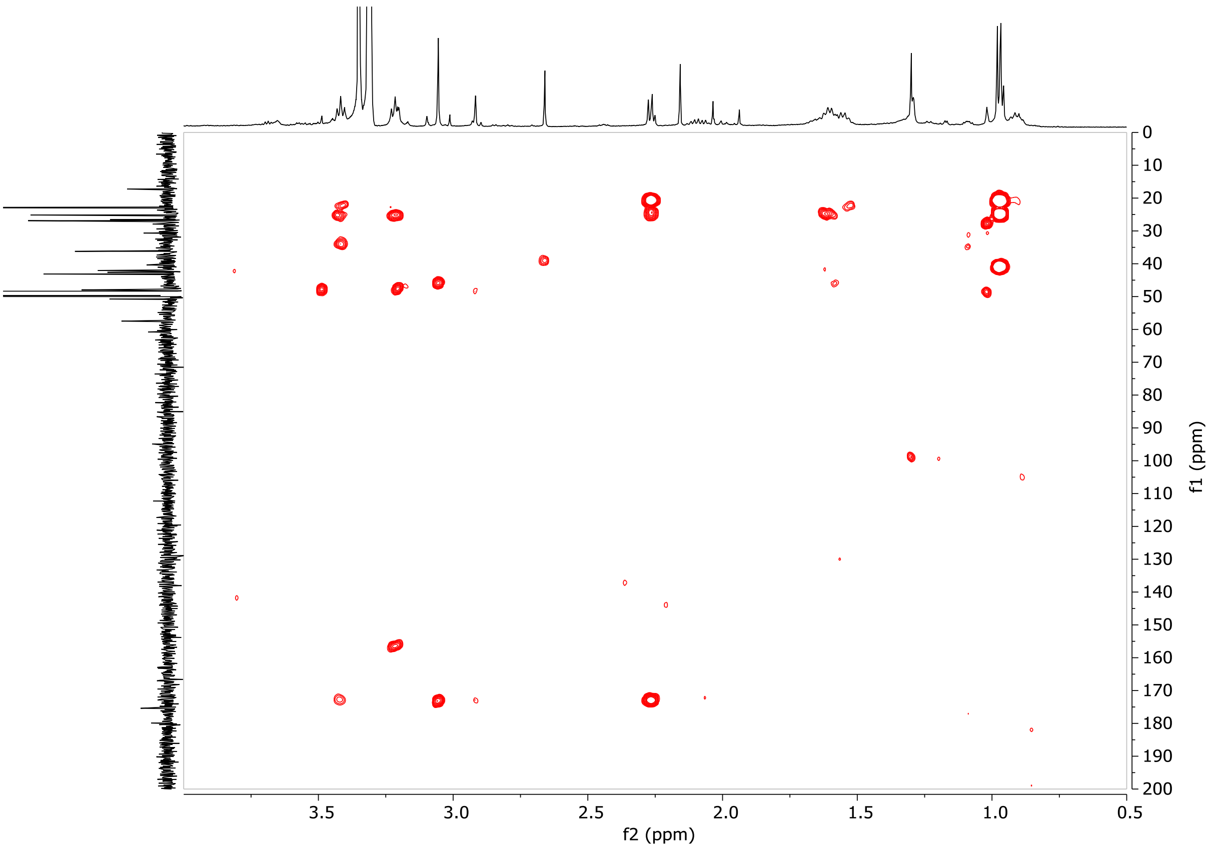

Figure S6-5. HMBC spectrum (600 MHz) of **6** in MeOH-*d*4.

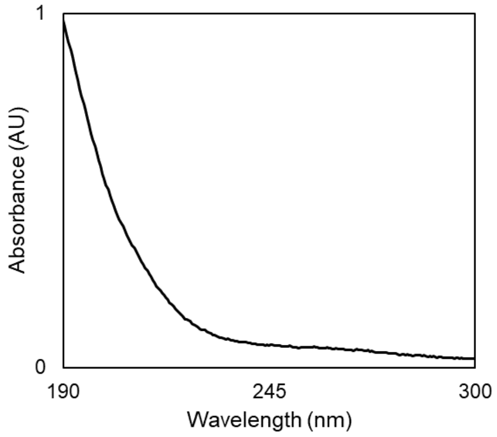

Figure S6-6. UV spectrum (c 0.15 mM) of **6** in H_2O.

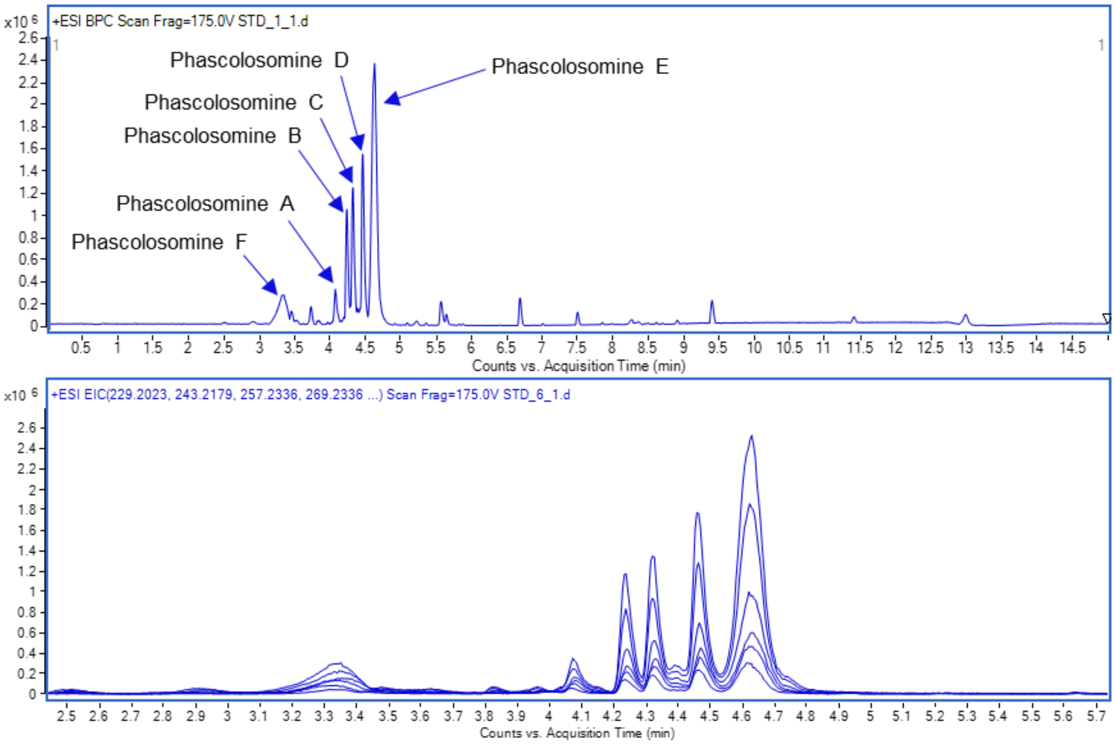

Figure S7-1. Overlaid LC-(+)HRESIMS base peak chromatograms of dilutions of a mixture of known concentrations of phascolosomines A-F (**1-6**). NOTE: concentrations of each compounds in the mixture differ to mimic sample concentrations, thus sample concentrations are within the calibration curves produced.

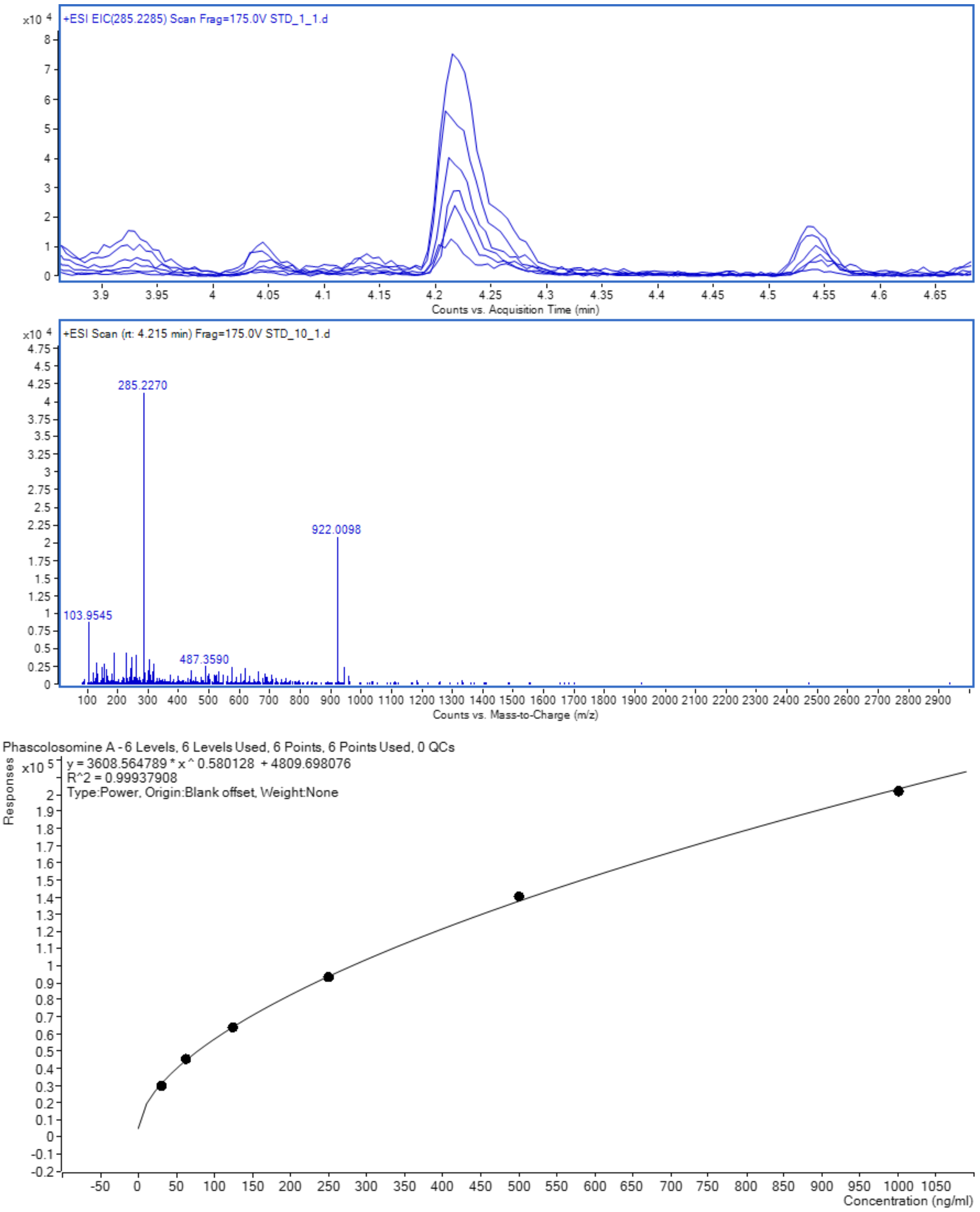

Figure S7-1. Overlaid extracted ion chromatogram of standards, MS spectrum and calibration curve for phascolosomine A (**1**).

Table S1. Quantification data for phascolosomine A (**1**).

Sample	Conc.	RT	Peak area	Curve Conc.	% Accuracy
STD_6_1	31.25	4.214	29363	37.1046	118.7
STD_5_1	62.5	4.217	45395	78.6266	125.8
STD_4_1	125	4.222	63664	140.8547	112.7
STD_3_1	250	4.212	93033	270.8614	108.3
STD_2_1	500	4.209	140100	548.571	109.7
STD_1_1	1000	4.215	202126	1031.875	103.2

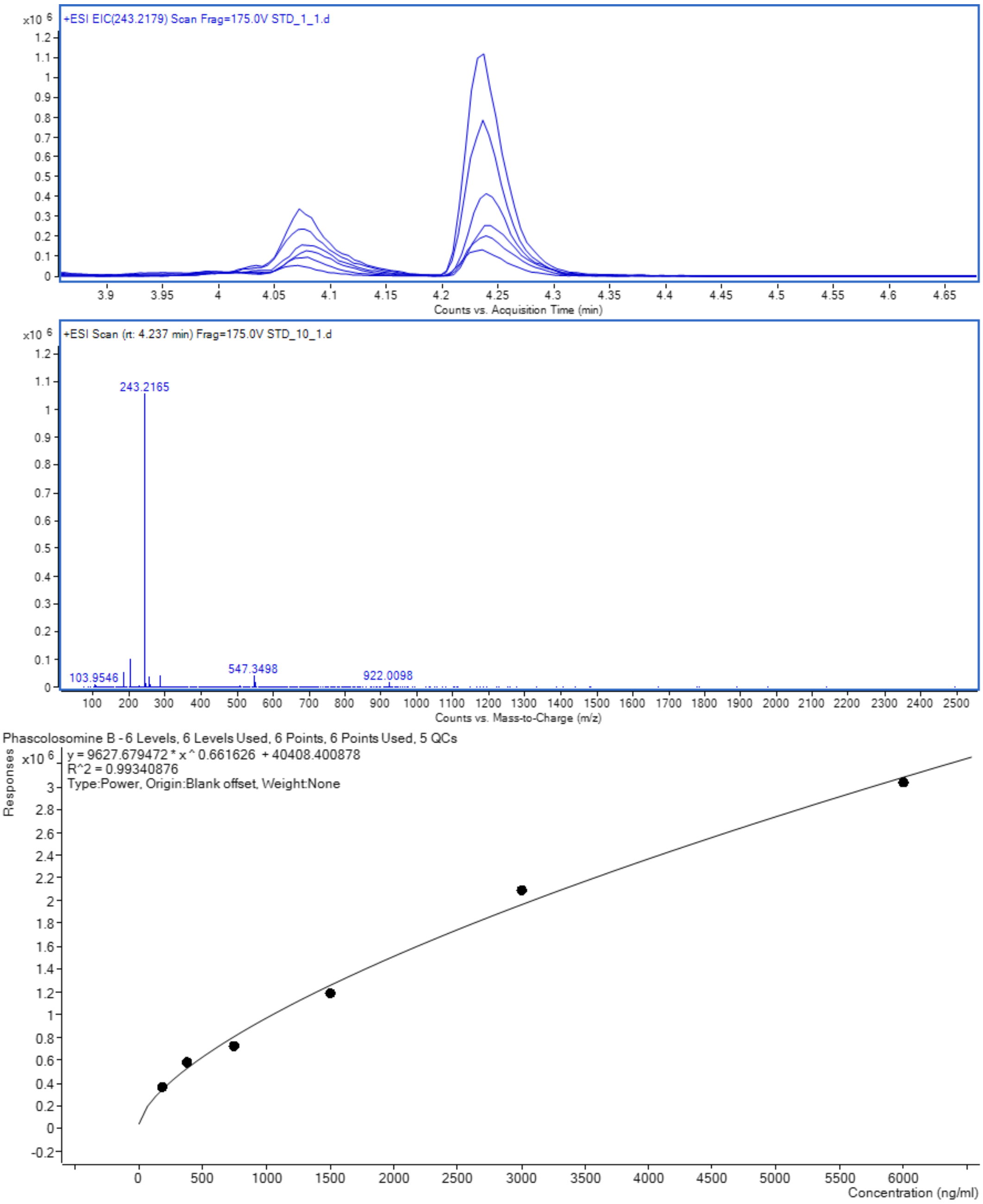

Figure S7-2. Overlaid extracted ion chromatogram of standards, MS spectrum and calibration curve for phascolosomine B (**2**).

Table S2. Quantification data for phascolosomine B (**2**).

Sample	Conc.	RT	Peak area	Curve Conc.	% Accuracy
STD_6_1	187.5	4.236	359279	237.5902	126.7
STD_5_1	375	4.239	584099	495.2459	132.1
STD_4_1	750	4.238	721694	681.8219	90.9
STD_3_1	1500	4.24	1181800	1436.829	95.8
STD_2_1	3000	4.237	2095131	3413.871	113.8
STD_1_1	6000	4.237	3041476	5996.625	99.9

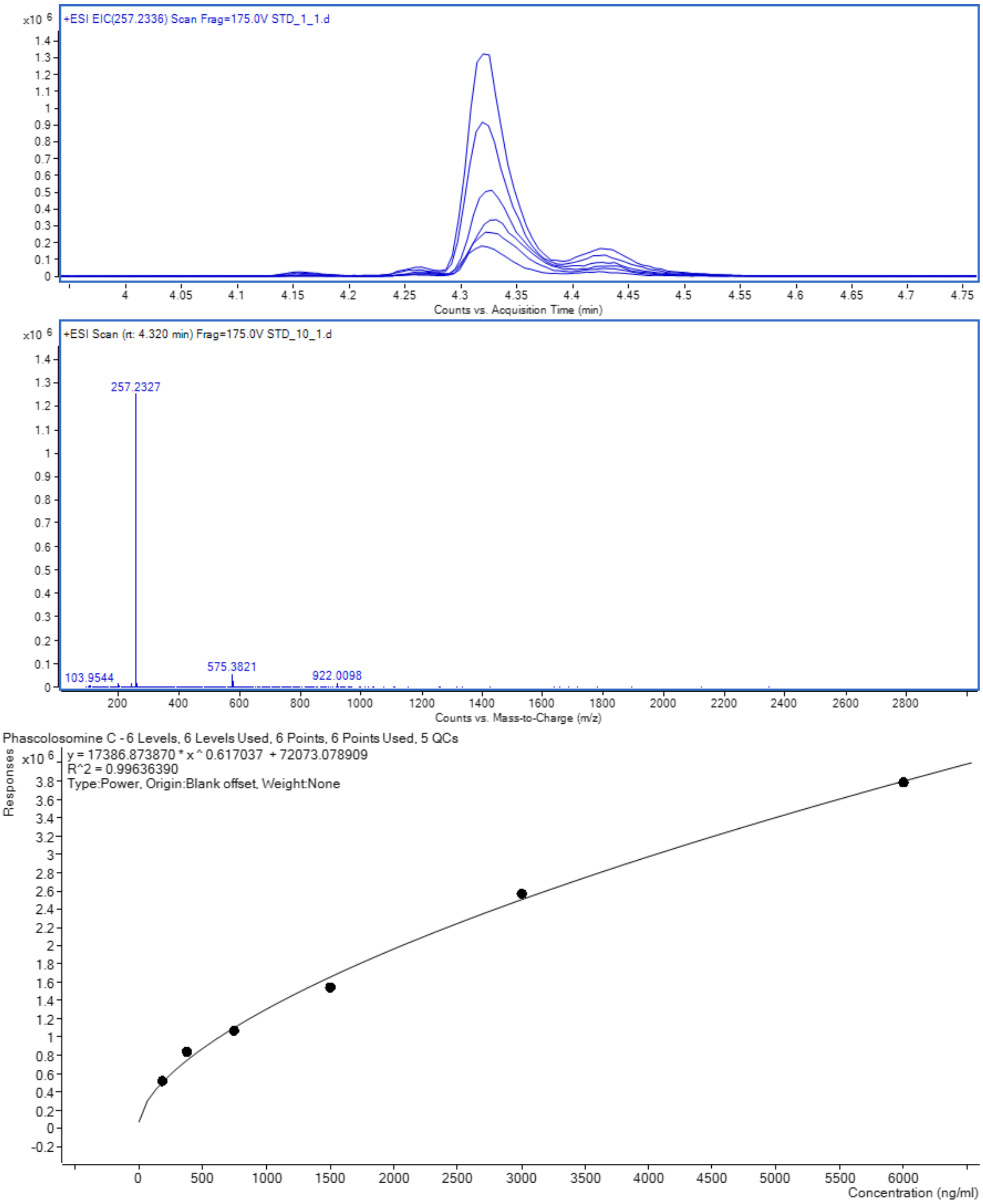

Figure S7-3. Overlaid extracted ion chromatogram of standards, MS spectrum and calibration curve for phascolosomine C (**3**).

Table S3. Quantification data for phascolosomine C (**3**).

Sample	Conc.	RT	Peak area	Curve Conc.	% Accuracy
STD_6_1	187.5	4.319	520458	246.7992	131.6
STD_5_1	375	4.322	844226	540.5035	144.1
STD_4_1	750	4.332	1068206	791.4499	105.5
STD_3_1	1500	4.328	1548933	1445.306	96.4
STD_2_1	3000	4.319	2573703	3291.203	109.7
STD_1_1	6000	4.32	3791221	6165.687	102.8

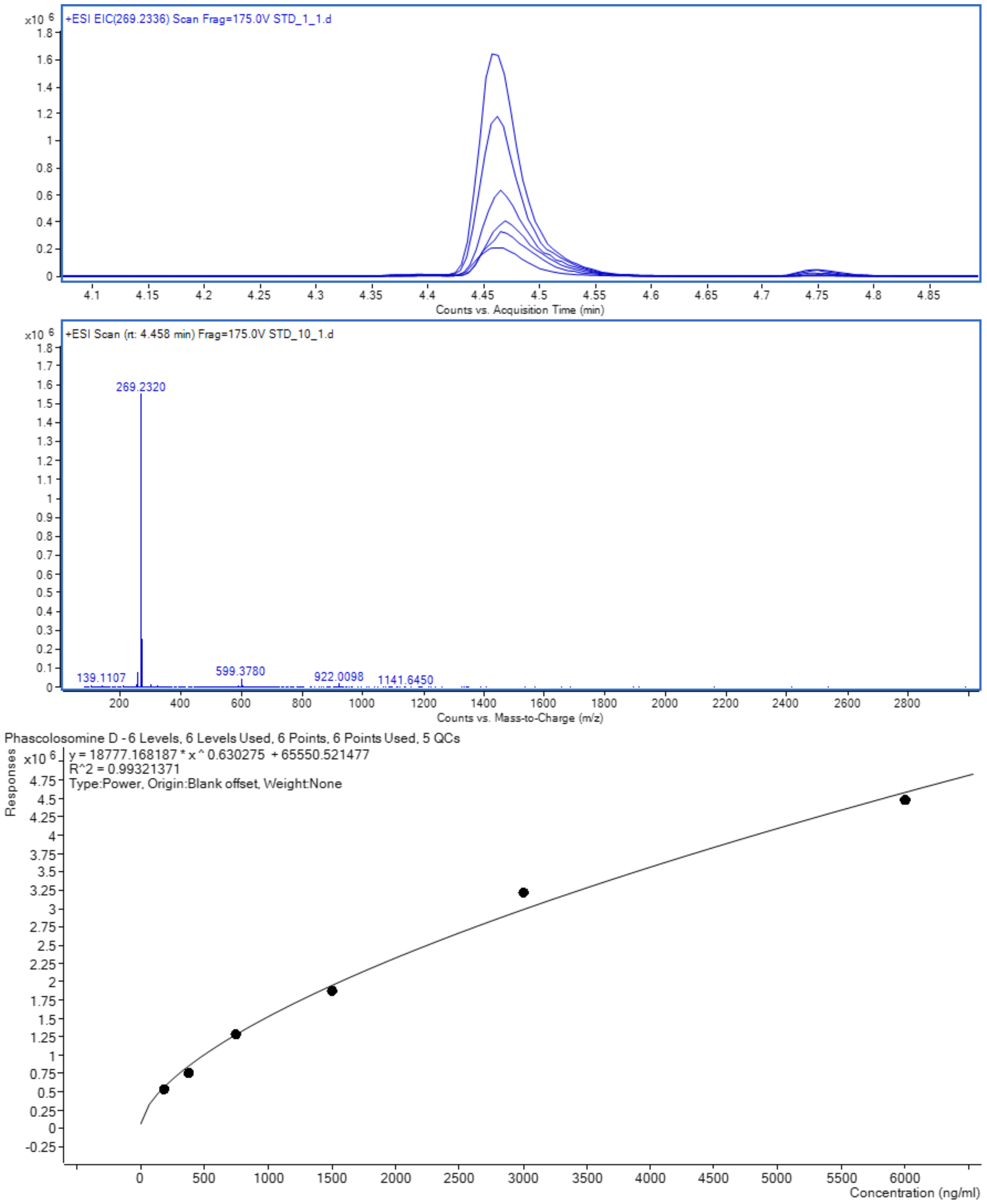

Figure S7-4. Overlaid extracted ion chromatogram of standards, MS spectrum and calibration curve for phascolosomine D (**4**).

Table S4. Quantification data for phascolosomine D (**4**).

Sample	Conc.	RT	Peak area	Curve Conc.	% Accuracy
STD_6_1	187.5	4.467	532326	201.6629	107.6
STD_5_1	375	4.465	762461	356.6155	95.1
STD_4_1	750	4.469	1279402	810.687	108.1
STD_3_1	1500	4.465	1876615	1488.724	99.2
STD_2_1	3000	4.462	3219277	3505.006	116.8
STD_1_1	6000	4.458	4483840	5929.1	98.8

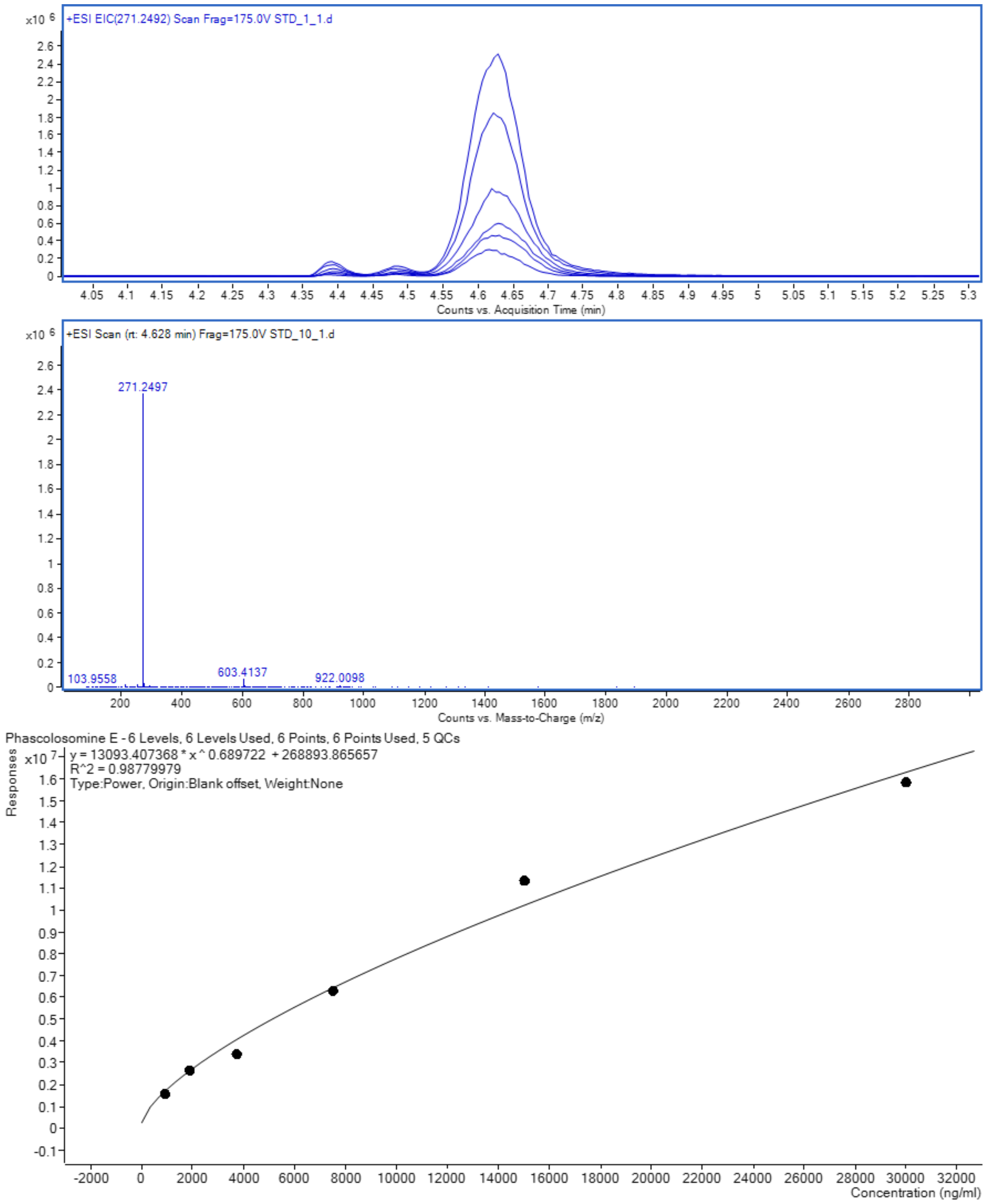

Figure S7-5. Overlaid extracted ion chromatogram of standards, MS spectrum and calibration curve for phascolosomine E (**5**).

Table S5. Quantification data for phascolosomine E (**5**).

Sample	Conc.	RT	Peak area	Curve Conc.	% Accuracy
STD_6_1	937.5	4.616	1560386	1023.688	109.2
STD_5_1	1875	4.63	2648349	2204.259	117.6
STD_4_1	3750	4.629	3409544	3179.383	84.8
STD_3_1	7500	4.619	6270722	7691.414	102.6
STD_2_1	15000	4.622	11313244	18095.1	120.6
STD_1_1	30000	4.628	15856154	29520.65	98.4

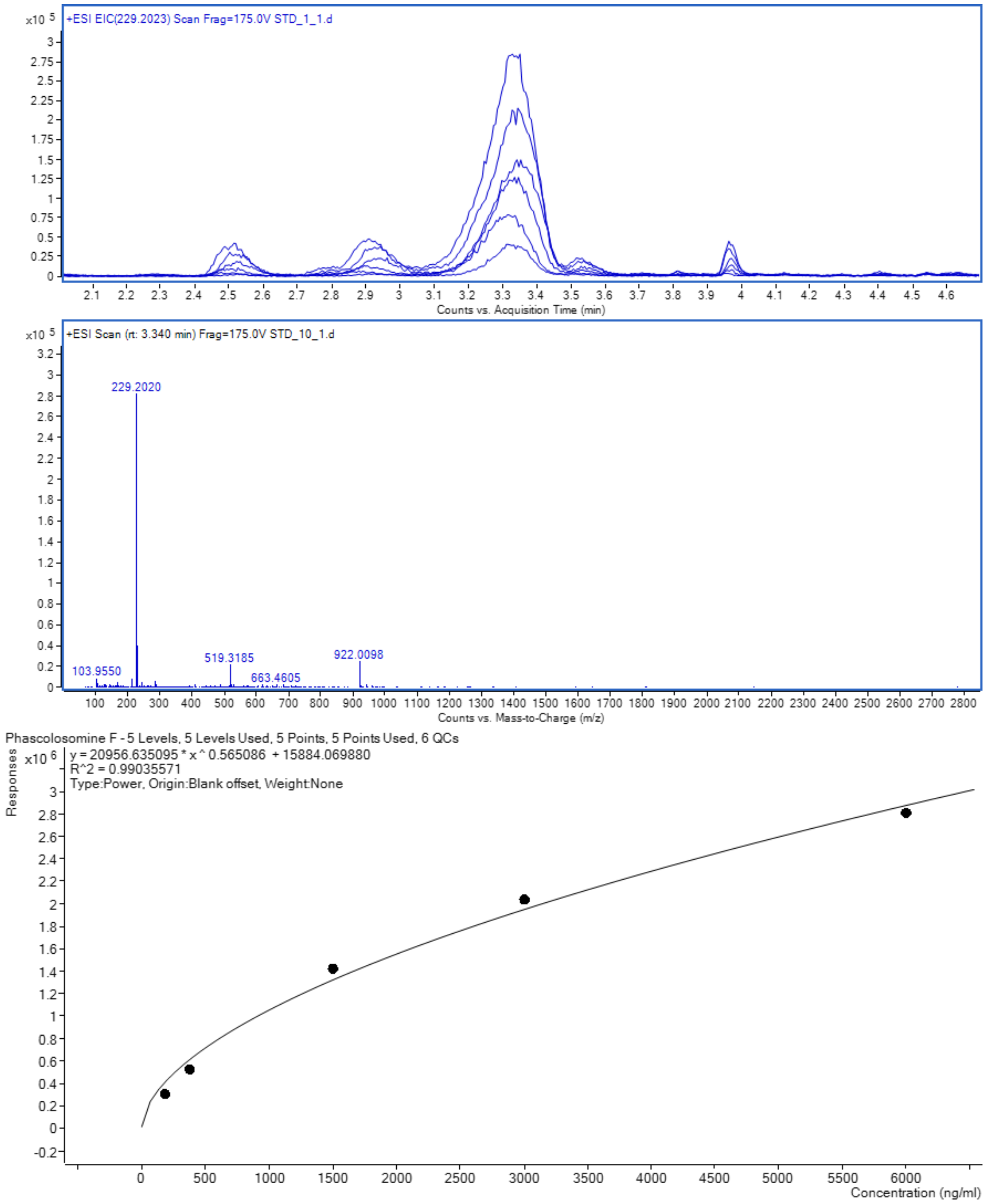

Figure S7-6. Overlaid extracted ion chromatogram of standards, MS spectrum and calibration curve for phascolosomine F (**6**).

Table S6. Quantification data for phascolosomine F (**6**).

Sample	Conc.	RT	Peak area	Curve Conc.	% Accuracy
STD_6_1	187.5	3.317	306103	115.0356	61.4
STD_5_1	375	3.315	529867	303.7637	81
STD_4_1	750	3.335	1229048	1158.629	154.5
STD_3_1	1500	3.342	1423719	1746.502	116.4
STD_2_1	3000	3.345	2032127	3278.128	109.3
STD_1_1	6000	3.351	2806005	5802.556	96.7

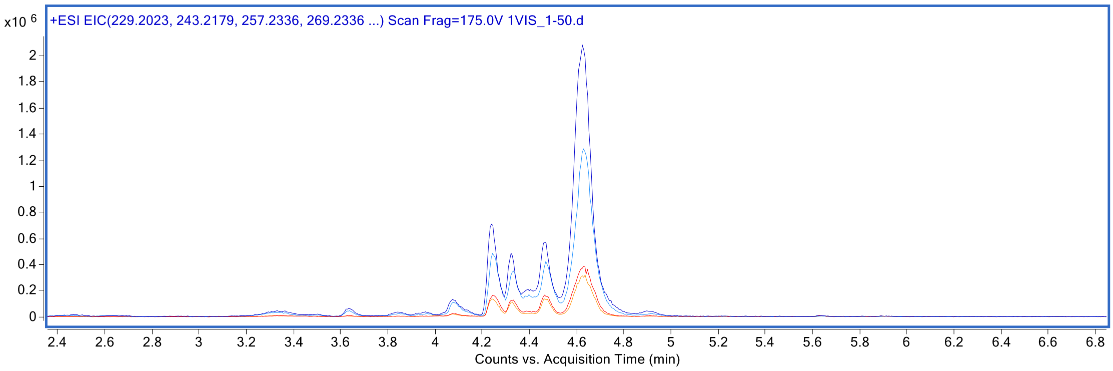

Figure S7-7. Overlaid extracted ion chromatograms for phascolosomines A-F (**1-6**) of the viscera extract (blue – 1:50, light blue – 1:100 dilution) and muscle extract (red – 1:50, orange – 1:100 dilution) of *P. granulatum* specimen 1.

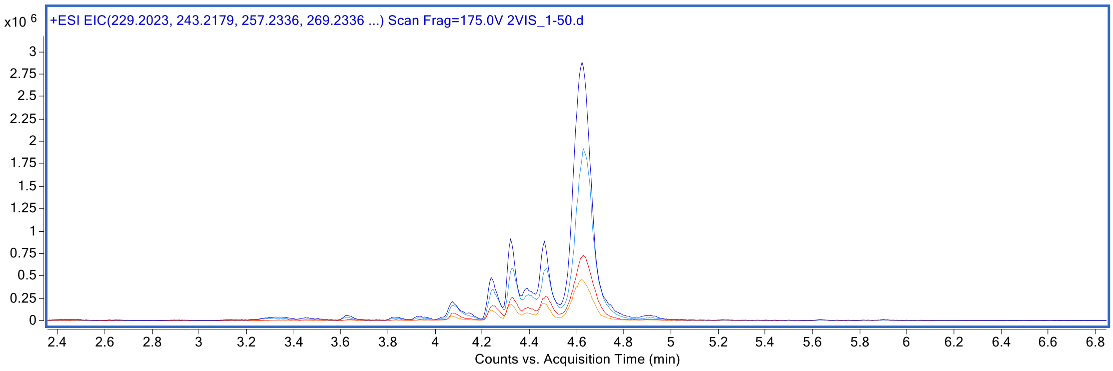

Figure S7-8. Overlaid extracted ion chromatograms for phascolosomines A-F (**1-6**) of the viscera extract (blue – 1:50, light blue – 1:100 dilution) and muscle extract (red – 1:50, orange – 1:100 dilution) of *P. granulatum* specimen 2.

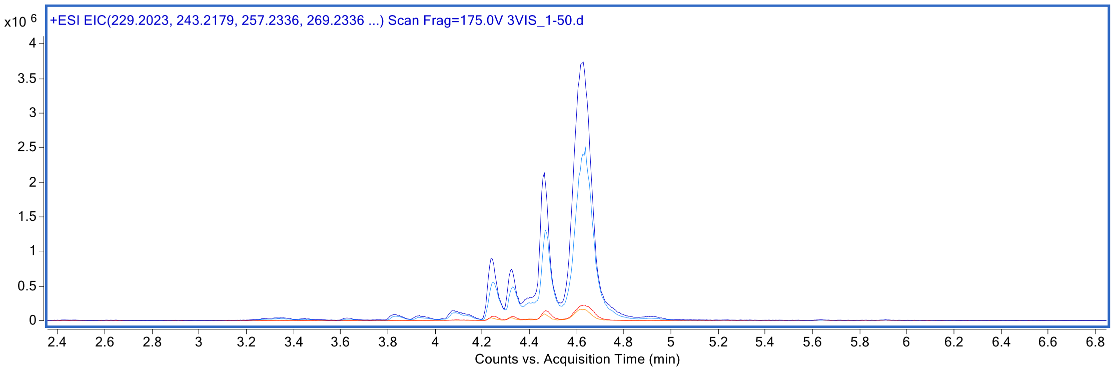

Figure S7-9. Overlaid extracted ion chromatograms for phascolosomines A-F (**1-6**) of the viscera extract (blue – 1:50, light blue – 1:100 dilution) and muscle extract (red – 1:50, orange – 1:100 dilution) of *P. granulatum* specimen 3.

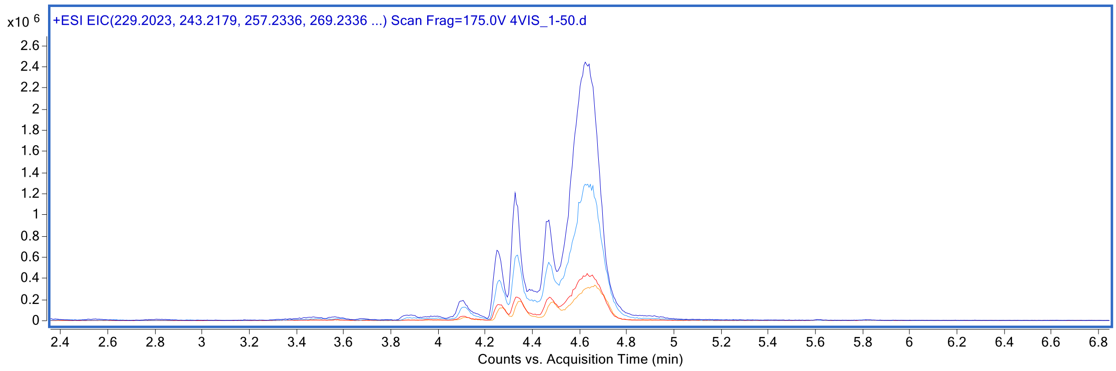

Figure S7-10. Overlaid extracted ion chromatograms for phascolosomines A-F (**1-6**) of the viscera extract (blue – 1:50, light blue – 1:100 dilution) and muscle extract (red – 1:50, orange – 1:100 dilution) of *P. granulatum* specimen 4.

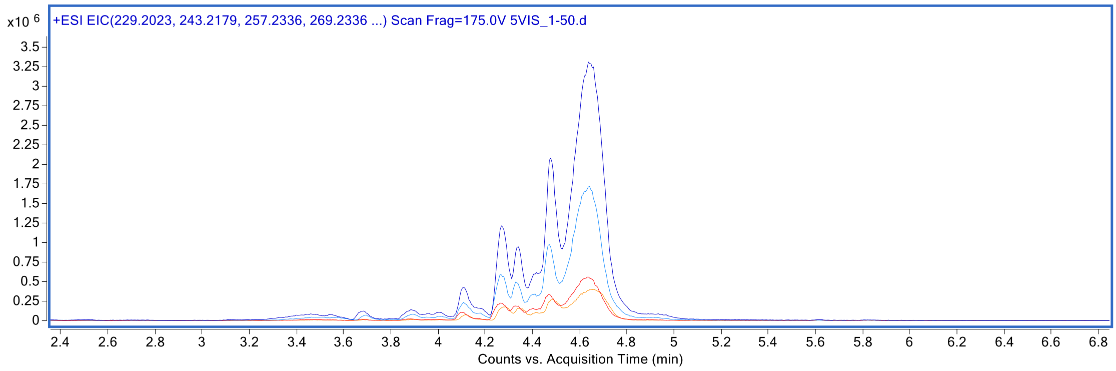

Figure S7-11. Overlaid extracted ion chromatograms for phascolosomines A-F (**1-6**) of the viscera extract (blue – 1:50, light blue – 1:100 dilution) and muscle extract (red – 1:50, orange – 1:100 dilution) of *P. granulatum* specimen 5.

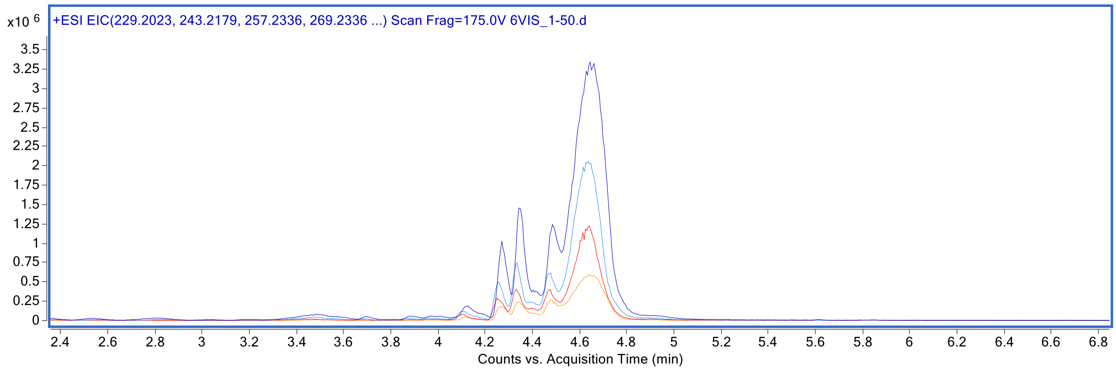

Figure S7-12. Overlaid extracted ion chromatograms for phascolosomines A-F (**1-6**) of the viscera extract (blue – 1:50, light blue – 1:100 dilution) and muscle extract (red – 1:50, orange – 1:100 dilution) of *P. granulatum* specimen 6.

Table S7. Quantification of compounds **1-6** for six dissected *Phascolosoma granulatum* specimens (A-F) using (+)HRESIMS. (NOTE: The peak area is from the integration of the peak associated with a particular compound in the extracted ion chromatogram. The concentration is then calculated from the calibration curve. This is then presented in text as g/100g dry weight by taking into consideration the dilution factors and biomass weight.)

Specimen	Tissue type	dry mass (mg)	Dilution	EIC peak area						Concentration (ng/mL)					
				**1**	**2**	**3**	**4**	**5**	**6**	**1**	**2**	**3**	**4**	**5**	**6**
A	Muscle	34.1	1:100	8855	360808	278638	347623	1682964	52941	4.6989	239.1197	89.6581	102.5635	1142.313	5.155
			1:50	11835	454021	357091	425713	2185527	58012	7.7475	338.4191	134.0278	141.4582	1668.464	6.0608
	viscera	24.9	1:100	27045	1299759	1054153	1115631	7013701	248678	32.1996	1659.034	774.6443	652.3356	9047.164	79.6445
			1:50	36643	1791879	1342397	1506409	11025872	347329	54.3535	2695.365	1146.129	1050.511	17432.51	143.859
B	Muscle	49.7	1:100	14957	276875	541309	527186	2549758	33277	11.5994	160.2557	263.0215	198.5819	2086.289	2.2667
			1:50	24149	428949	796901	790402	4177120	39487	26.4893	310.5763	492.2605	377.5719	4267.684	3.0682
	viscera	33.4	1:100	28922	888077	1747096	1612871	10241946	186678	36.1499	932.9256	1756.685	1170.724	15664.64	47.9466
			1:50	44718	1121344	2428017	2205424	15446500	314749	76.6154	1327.201	2994.618	1923.368	28421.32	120.848
C	Muscle	51.4	1:100	247	80912	95206	220001	802153	19946	0	24.9634	15.7309	49.6318	390.1167	0.9162
			1:50	4004	141351	147996	383740	1224263	17780	0	58.0094	32.1551	119.9788	720.1351	0.7476
	viscera	77.1	1:100	16180	1490499	1444688	3489188	13240407	185174	13.2824	2040.508	1290.985	3982.596	22730.39	47.265
			1:50	25962	2213488	2003552	5491156	20280457	290545	30.0109	3709.532	2193.293	8177.719	42178.06	104.8919
D	Muscle	32.8	1:100	9911	341168	608845	436265	2966046	32199	5.7064	219.7227	318.2322	147.0615	2597.763	2.1383
			1:50	16609	453436	777514	583091	3923781	37499	13.8948	337.76	472.9987	233.0181	3897.591	2.8002
	viscera	56.3	1:100	20542	1092233	1997636	1329901	11432295	143929	20.0439	1275.471	2182.806	862.0404	18371.83	30.2612
			1:50	34257	1721458	3336552	2311026	20137841	232920	48.3986	2536.882	5012.632	2071.528	41748.71	70.9322
E	Muscle	48.1	1:100	5180	361620	515972	650188	3824426	62463	1.8647	239.9332	243.3613	276.9749	3755.321	6.908
			1:50	9168	430248	655000	770596	4844681	100794	4.989	311.9983	358.2408	362.6712	5291.101	16.1101
	viscera	66.7	1:100	43106	1092680	1561386	2004783	14846740	437896	71.919	1276.26	1464.184	1653.247	26835.4	216.7785
			1:50	79962	2257551	2878564	4280634	29095506	803307	208.6419	3821.707	3945.905	5508.473	71178.59	634.3615
F	Muscle	140.0	1:100	8842	536768	824328	618785	5564937	79584	4.6869	435.8646	520.0092	256.0528	6468.751	10.6051
			1:50	13943	813039	1284676	957267	9389452	151152	10.278	816.3947	1067.333	511.6602	13810.18	33.0004
	viscera	100.7	1:100	41737	1332320	2177053	1418577	16878269	381955	68.0262	1722.252	2509.289	955.0051	32319.2	170.2056
			1:50	71164	2631347	4320979	2668436	30966168	770418	170.6629	4817.575	7621.479	2602.421	77908.5	589.1267

Table S8: LD_{50} activity for toxicity in brine shrimp lethality assay.

Compound	LD50 (μM)	
	24 h	48 h
Phascolosomine A (**1**)	>100	>100
Phascolosomine B (**2**)	>100	>100
Phascolosomine C (**3**)	>100	>100
Phascolosomine D (**4**)	>500	>500
Phascolosomine E (**5**)	>500	473.
Phascolosomine F (**6**)	>100	>100

Table S9: AC_{50} activity for cytotoxicity against multiple human cancer cell lines.

Compound	AC50 (μg/mL)				
	A549	A2058	HepG2	MCF-7	MiaPaca-2
Phascolosomine A (**1**)	>20	>20	>20	>20	>20
Phascolosomine B (**2**)	>20	>20	>20	>20	>20
Phascolosomine C (**3**)	>20	>20	>20	>20	>20
Phascolosomine D (**4**)	>20	>20	>20	>20	>20
Phascolosomine E (**5**)	>20	>20	>20	>20	>20
Phascolosomine F (**6**)	>20	>20	>20	>20	>20

Table S10: MIC antifungal activity against multiple fungal strains. NOTE: compounds **1** and **6** were not tested due to a lack of material.

Compound	MIC (μg/mL)					
	C. albicans ATCC64124	*C. glabrata* ATCC2001	*C. krusei* ATCC6258	*C. parapsilosis* ATCC22019	*C. tropicalis* ATCC750	*A. fumigatus* ATCC46645
Phascolosomine B (**2**)	>64	>64	>64	>64	>64	>64
Phascolosomine C (**3**)	>64	>64	>64	>64	>64	>64
Phascolosomine D (**4**)	>64	>64	>64	>64	>64	>32
Phascolosomine E (**5**)	>64	>64	>64	>64	>64	>64

Table S11: MIC antibacterial activity against bacterial strains. NOTE: compounds **1** and **6** were not tested due to a lack of material.

Compound	MIC (μg/mL)		
	A. baumannii ATCC19606	*E. coli* ATCC25922	MSSA ATCC29213
Phascolosomine B (**2**)	>64	>64	>64
Phascolosomine C (**3**)	>64	>64	>64
Phascolosomine D (**4**)	>32	>64	>64
Phascolosomine E (**5**)	>64	>64	>64

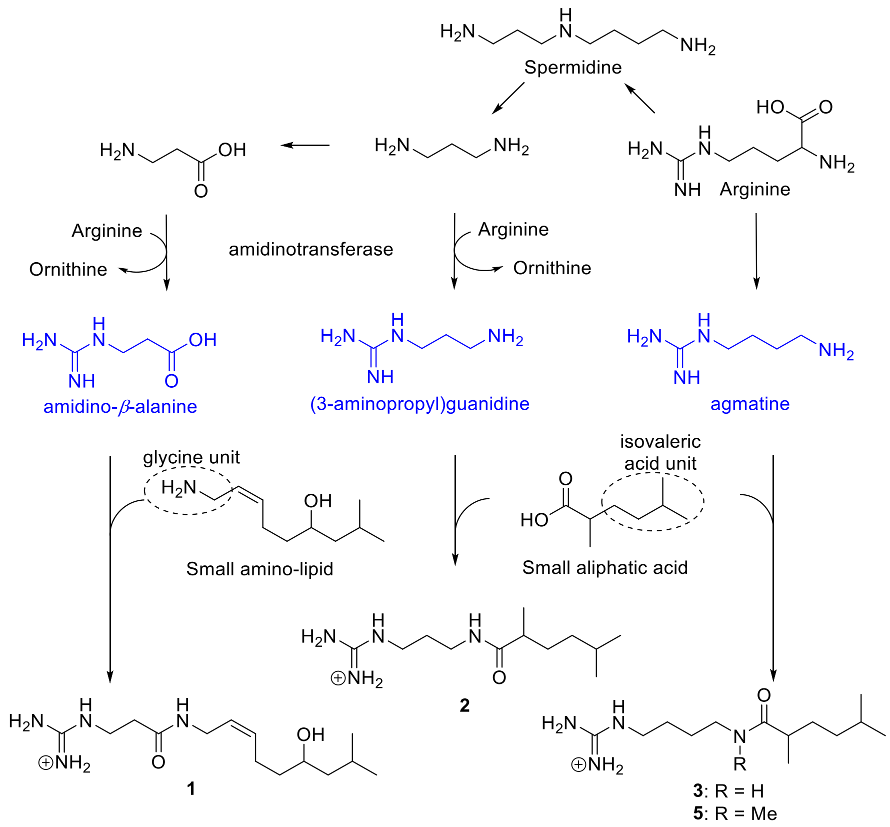

Figure S8. A plausible biosynthetic pathway of the three guanidine moieties (blue) required for the biosynthesis of phascolosomines A-F, (**1**– **6**) from polyamine precursors.

Data 1. Gaussian script used for the prediction of ECD in Gaussian 16.

```
%mem=100gb
%nproc=40
%chk=GolfB-S-conf-1.chk

# M062X/6-311+G(2d,p) Opt freq=noraman integral(ultrafinegrid)
scrf=(iefpcm,read,solvent=water)

+1 1
List of coordinates

radii=UFF

--link1--
%mem=100gb
%nproc=40
%chk=GolfB-S-conf-1.chk

# b3lyp/6-311G(2d,p) td(nstate=50) guess=read geom=check
integral(ultrafinegrid) scrf=(iefpcm,read,solvent=water)

+1 1

radii=bondi
```

Data 2. Geometry optimised Cartesian coordinates and energy calculations for each conformer predicted for phascolosomine B (**2**).

**Conformer 1**				**Conformer 2**			
**Atom**	**X**	**Y**	**Z**	**Atom**	**X**	**Y**	**Z**
C	-4.336723	4.159306	-3.60637	C	-2.71299	4.50669	-2.72663
N	-4.906568	3.129205	-2.99616	N	-3.24256	3.396197	-2.23243
N	-3.048767	4.422539	-3.405874	N	-3.18095	5.020402	-3.85987
N	-5.056026	4.930529	-4.422395	N	-1.7019	5.104813	-2.0951
C	-4.204571	2.206236	-2.108273	C	-4.33705	2.662165	-2.86173
C	-5.127085	1.055883	-1.742347	C	-4.6868	1.445835	-2.02172
C	-4.435816	0.067621	-0.80715	C	-5.84945	0.673473	-2.63867
N	-5.319569	-1.020691	-0.434053	N	-6.22415	-0.47456	-1.8357
C	-6.299768	-0.857105	0.479737	C	-6.9583	-0.3461	-0.7105
C	-7.244792	-2.029536	0.676959	C	-7.19099	-1.61536	0.091573
O	-6.427402	0.187182	1.110997	O	-7.40091	0.739143	-0.34791
C	-8.657973	-1.611671	0.251479	C	-8.68446	-1.79778	0.36632
C	-7.213546	-2.47275	2.138894	C	-6.38948	-1.52103	1.392417
C	-8.744116	-1.165405	-1.205218	C	-9.51096	-1.96569	-0.90509
C	-10.125399	-0.66642	-1.637512	C	-11.0235	-2.02059	-0.67474
C	-11.193658	-1.752092	-1.516508	C	-11.4249	-3.18926	0.224124
C	-10.066536	-0.139836	-3.070064	C	-11.7566	-2.10563	-2.01177
H	-5.901624	3.006212	-3.116728	H	-2.86609	3.039698	-1.36576
H	-2.53263	3.955648	-2.678	H	-3.89397	4.547607	-4.39047
H	-2.605702	5.203205	-3.863559	H	-2.755	5.835844	-4.27046
H	-4.62399	5.688876	-4.925062	H	-1.37391	6.008041	-2.3975
H	-5.974086	4.640149	-4.719944	H	-1.41842	4.792433	-1.1801
H	-3.884762	2.732913	-1.204475	H	-4.03345	2.347689	-3.86423
H	-3.318186	1.825246	-2.622249	H	-5.20742	3.31821	-2.9503
H	-5.443166	0.536889	-2.650662	H	-4.96892	1.761448	-1.01467
H	-6.019207	1.442996	-1.243407	H	-3.8156	0.790977	-1.94247
H	-4.115837	0.57573	0.103377	H	-5.58761	0.317465	-3.63498
H	-3.55411	-0.360696	-1.28363	H	-6.72088	1.323666	-2.72936
H	-5.303475	-1.86779	-0.979885	H	-5.84378	-1.37846	-2.06741
H	-6.91944	-2.858577	0.04163	H	-6.83187	-2.47094	-0.4884
H	-8.990196	-0.799502	0.906615	H	-9.0424	-0.92984	0.929383
H	-9.322208	-2.460664	0.430084	H	-8.80048	-2.67084	1.01309
H	-6.213526	-2.790339	2.438293	H	-5.32385	-1.39053	1.195844
H	-7.521826	-1.649534	2.784614	H	-6.73791	-0.67346	1.984726
H	-7.898767	-3.307691	2.290778	H	-6.5209	-2.43055	1.979801
H	-8.446068	-1.99478	-1.859697	H	-9.19853	-2.88101	-1.42376
H	-8.020652	-0.360324	-1.377842	H	-9.29555	-1.13407	-1.58584
H	-10.402053	0.164041	-0.977207	H	-11.3194	-1.08831	-0.17921
H	-10.912213	-2.626821	-2.110863	H	-11.0758	-4.13341	-0.20542
H	-11.3358	-2.075853	-0.484767	H	-11.0056	-3.0988	1.226973
H	-12.154903	-1.390292	-1.887018	H	-12.5109	-3.24643	0.321135
H	-9.798424	-0.946311	-3.758889	H	-11.4914	-3.03153	-2.53052
H	-11.032592	0.260835	-3.383295	H	-12.839	-2.09985	-1.86905
H	-9.320086	0.651061	-3.1713	H	-11.4939	-1.26859	-2.66197

Conformer 1		Conformer 2
0	**NImag**	0
-767.231368	**Σ Electronic and thermal Free Energy**	-767.231333
0.1873	**mol Fraction**	0.1805

**Conformer 3**				**Conformer 4**			
**Atom**	**X**	**Y**	**Z**	**Atom**	**X**	**Y**	**Z**
C	-3.05921	4.373323	-3.70231	C	-3.68853	4.535904	-3.40214
N	-3.6152	3.208827	-3.39402	N	-4.37681	3.509887	-2.92081
N	-3.16196	5.401232	-2.86588	N	-2.39454	4.658653	-3.12107
N	-2.38979	4.514036	-4.84598	N	-4.29106	5.445776	-4.16839
C	-4.34276	2.95669	-2.15369	C	-3.79875	2.453878	-2.09386
C	-4.81386	1.512634	-2.11946	C	-4.82608	1.357756	-1.86834
C	-5.5686	1.229377	-0.83105	C	-4.24722	0.236899	-1.00955
N	-6.01345	-0.15216	-0.80435	N	-5.2245	-0.80067	-0.74334
C	-6.73994	-0.63752	0.220791	C	-6.19263	-0.64299	0.185603
C	-7.15074	-2.09731	0.133802	C	-7.24068	-1.7393	0.249435
O	-7.05119	0.067505	1.176047	O	-6.23755	0.346274	0.909542
C	-8.67794	-2.20504	0.176342	C	-8.47807	-1.24801	-0.52066
C	-6.50555	-2.8677	1.286663	C	-7.55777	-2.08478	1.701843
C	-9.36431	-1.48703	-0.9818	C	-9.59611	-2.28569	-0.59109
C	-10.8936	-1.48321	-0.90922	C	-10.7004	-1.93968	-1.59366
C	-11.473	-2.897	-0.92008	C	-11.3664	-0.60368	-1.26816
C	-11.4717	-0.66598	-2.06269	C	-11.7415	-3.05595	-1.63624
H	-3.52395	2.452644	-4.057	H	-5.36987	3.482298	-3.10312
H	-2.71138	6.278716	-3.06982	H	-1.95864	4.075762	-2.42533
H	-3.61516	5.304692	-1.97229	H	-1.86575	5.44225	-3.4695
H	-2.03756	5.414285	-5.12924	H	-3.76141	6.194436	-4.58505
H	-2.37178	3.770521	-5.52553	H	-5.21577	5.275162	-4.53048
H	-5.20131	3.630798	-2.09283	H	-3.47855	2.870026	-1.13418
H	-3.68497	3.150259	-1.30231	H	-2.92371	2.043722	-2.60506
H	-3.94946	0.847261	-2.19399	H	-5.14683	0.952236	-2.83088
H	-5.46321	1.320352	-2.97792	H	-5.70298	1.769503	-1.36287
H	-6.43713	1.887223	-0.74418	H	-3.90894	0.637702	-0.05304
H	-4.92944	1.421683	0.03467	H	-3.3919	-0.22271	-1.50478
H	-5.78293	-0.75963	-1.57541	H	-5.261	-1.60316	-1.3521
H	-6.79621	-2.50888	-0.81598	H	-6.85071	-2.62817	-0.25586
H	-9.02807	-1.79191	1.128016	H	-8.17797	-0.97572	-1.53913
H	-8.93643	-3.2667	0.172592	H	-8.83721	-0.33399	-0.03951
H	-5.41692	-2.80164	1.251007	H	-6.65096	-2.34605	2.249201
H	-6.84464	-2.46335	2.241462	H	-8.02441	-1.23329	2.199251
H	-6.78722	-3.92023	1.236975	H	-8.23987	-2.93309	1.752664
H	-9.05589	-1.94736	-1.92924	H	-10.0521	-2.41033	0.39727
H	-9.01894	-0.44725	-1.0157	H	-9.16607	-3.25679	-0.86191
H	-11.1817	-1.00057	0.032194	H	-10.2393	-1.86125	-2.58533
H	-11.1397	-3.43503	-1.8129	H	-11.7745	-0.62263	-0.25283
H	-11.1684	-3.4726	-0.04518	H	-10.6656	0.229745	-1.33476
H	-12.5644	-2.86655	-0.93499	H	-12.1897	-0.40411	-1.95704
H	-11.2119	-1.12665	-3.02032	H	-12.2335	-3.15212	-0.66399
H	-12.5604	-0.61224	-2.00133	H	-12.5113	-2.84846	-2.3821
H	-11.0794	0.35313	-2.06228	H	-11.2827	-4.01705	-1.8777
	0			**NImag**		0	
	-767.231317			**Σ Electronic and thermal Free Energy**		-767.229310	
	0.1775			**mol Fraction**		0.0211	

**Conformer 5**				**Conformer 6**			
**Atom**	**X**	**Y**	**Z**	**Atom**	**X**	**Y**	**Z**
C	-2.26294	4.51255	-2.73728	C	-4.66051	4.312538	-3.34737
N	-2.91358	3.452108	-2.27894	N	-5.16002	3.183302	-2.86417
N	-2.76152	5.212305	-3.75122	N	-3.3772	4.606764	-3.16266
N	-1.09743	4.870552	-2.198	N	-5.43894	5.153166	-4.02902
C	-4.1567	2.946679	-2.8551	C	-4.3769	2.194935	-2.1276
C	-4.59588	1.695746	-2.1134	C	-5.26454	1.031203	-1.72092
C	-5.89001	1.141082	-2.70209	C	-4.47234	-0.00199	-0.92427
N	-6.35136	-0.03384	-1.98848	N	-5.3006	-1.11504	-0.50394
C	-7.02556	0.059496	-0.82237	C	-6.17	-1.00839	0.526301
C	-7.4712	-1.25117	-0.19916	C	-7.07444	-2.21359	0.743529
O	-7.29643	1.14487	-0.31854	O	-6.23537	0.007672	1.208163
C	-8.96714	-1.43797	-0.4997	C	-8.10608	-2.29092	-0.40251
C	-7.17461	-1.24216	1.298743	C	-7.72538	-2.16588	2.119819
C	-9.52836	-2.75449	0.033278	C	-8.88119	-0.99552	-0.63103
C	-10.9146	-3.10451	-0.51425	C	-10.0095	-1.1149	-1.65941
C	-11.9458	-2.02669	-0.18343	C	-9.49047	-1.53459	-3.03416
C	-11.3682	-4.45836	0.027103	C	-10.7652	0.208281	-1.76137
H	-2.51467	2.956955	-1.49438	H	-6.14102	2.997875	-3.01619
H	-3.64212	4.967166	-4.17276	H	-2.75288	3.961437	-2.7072
H	-2.26359	6.004203	-4.12493	H	-2.97937	5.441275	-3.56288
H	-0.66242	5.74191	-2.45462	H	-5.09024	6.053985	-4.31448
H	-0.73933	4.391479	-1.38749	H	-6.43289	4.99953	-4.08558
H	-3.99568	2.716745	-3.9122	H	-3.94924	2.664789	-1.23752
H	-4.93078	3.715538	-2.77934	H	-3.55974	1.836161	-2.76003
H	-4.7575	1.928424	-1.05842	H	-5.68691	0.558488	-2.61148
H	-3.81295	0.936285	-2.17882	H	-6.08936	1.395126	-1.10381
H	-5.74625	0.866882	-3.74723	H	-4.04615	0.463725	-0.03434
H	-6.67503	1.897298	-2.6549	H	-3.65205	-0.39905	-1.52222
H	-6.11039	-0.94623	-2.34177	H	-5.30668	-1.95449	-1.06209
H	-6.92034	-2.07094	-0.67026	H	-6.44815	-3.10859	0.682514
H	-9.11868	-1.39706	-1.58421	H	-8.80404	-3.10227	-0.17237
H	-9.50905	-0.59113	-0.06984	H	-7.5928	-2.5754	-1.32408
H	-6.12171	-1.02764	1.488258	H	-6.9714	-2.14965	2.90724
H	-7.77439	-0.47818	1.795642	H	-8.34354	-1.27652	2.236118
H	-7.40736	-2.21025	1.741517	H	-8.3527	-3.04703	2.261226
H	-9.58663	-2.72004	1.126796	H	-8.19027	-0.21033	-0.96474
H	-8.83569	-3.56608	-0.21757	H	-9.30813	-0.64946	0.314994
H	-10.8341	-3.17958	-1.60515	H	-10.7069	-1.88318	-1.30514
H	-11.9985	-1.87382	0.898982	H	-8.7216	-0.83476	-3.37705
H	-11.7007	-1.07047	-0.64792	H	-9.05576	-2.53483	-3.0224
H	-12.9383	-2.3219	-0.52957	H	-10.2981	-1.53195	-3.76892
H	-11.4729	-4.41544	1.11513	H	-10.0993	0.997087	-2.1236
H	-12.3346	-4.74634	-0.39126	H	-11.6031	0.131459	-2.45706
H	-10.6463	-5.24244	-0.21071	H	-11.1556	0.517832	-0.78971

Conformer 5		Conformer 6
0	**NImag**	0
-767.230428	**Σ Electronic and thermal Free Energy**	-767.230126
0.0692	**mol Fraction**	0.0502

Conformer 7				Conformer 8			
**Atom**	**X**	**Y**	**Z**	**Atom**	**X**	**Y**	**Z**
C	-4.59253	4.100647	-3.58103	C	-4.59253	4.100647	-3.58103
N	-5.14684	3.013634	-3.06114	N	-5.14684	3.013634	-3.06114
N	-3.32577	4.397266	-3.30561	N	-3.32577	4.397266	-3.30561
N	-5.30122	4.897241	-4.38082	N	-5.30122	4.897241	-4.38082
C	-4.45352	2.095207	-2.16164	C	-4.45352	2.095207	-2.16164
C	-5.35832	0.919818	-1.83585	C	-5.35832	0.919818	-1.83585
C	-4.67732	-0.04438	-0.86755	C	-4.67732	-0.04438	-0.86755
N	-5.55519	-1.13693	-0.49287	N	-5.55519	-1.13693	-0.49287
C	-6.58376	-0.94692	0.363102	C	-6.58376	-0.94692	0.363102
C	-7.48874	-2.14166	0.615309	C	-7.48874	-2.14166	0.615309
O	-6.74668	0.120312	0.944473	O	-6.74668	0.120312	0.944473
C	-8.96909	-1.76716	0.469603	C	-8.96909	-1.76716	0.469603
C	-7.19293	-2.65065	2.031069	C	-7.19293	-2.65065	2.031069
C	-9.4767	-1.68254	-0.97104	C	-9.4767	-1.68254	-0.97104
C	-8.882	-0.57755	-1.85082	C	-8.882	-0.57755	-1.85082
C	-9.18241	0.816716	-1.30313	C	-9.18241	0.816716	-1.30313
C	-9.41601	-0.71047	-3.27634	C	-9.41601	-0.71047	-3.27634
H	-6.12558	2.846477	-3.2478	H	-6.12558	2.846477	-3.2478
H	-2.90273	5.233856	-3.67416	H	-2.90273	5.233856	-3.67416
H	-2.81087	3.88159	-2.61091	H	-2.81087	3.88159	-2.61091
H	-4.87238	5.691746	-4.82745	H	-4.87238	5.691746	-4.82745
H	-6.21453	4.618859	-4.70189	H	-6.21453	4.618859	-4.70189
H	-4.1783	2.620353	-1.2423	H	-4.1783	2.620353	-1.2423
H	-3.54083	1.740843	-2.64797	H	-3.54083	1.740843	-2.64797
H	-5.62034	0.391758	-2.75658	H	-5.62034	0.391758	-2.75658
H	-6.28203	1.281478	-1.37639	H	-6.28203	1.281478	-1.37639
H	-4.38548	0.485465	0.040389	H	-4.38548	0.485465	0.040389
H	-3.77903	-0.46973	-1.3145	H	-3.77903	-0.46973	-1.3145
H	-5.50949	-1.99881	-1.01341	H	-5.50949	-1.99881	-1.01341
H	-7.24747	-2.93098	-0.10338	H	-7.24747	-2.93098	-0.10338
H	-9.14184	-0.82861	1.003111	H	-9.14184	-0.82861	1.003111
H	-9.55268	-2.53149	0.989663	H	-9.55268	-2.53149	0.989663
H	-6.13452	-2.88436	2.158133	H	-6.13452	-2.88436	2.158133
H	-7.46912	-1.89107	2.764281	H	-7.46912	-1.89107	2.764281
H	-7.77188	-3.55237	2.232472	H	-7.77188	-3.55237	2.232472
H	-10.5641	-1.54505	-0.9485	H	-10.5641	-1.54505	-0.9485
H	-9.3001	-2.64878	-1.45687	H	-9.3001	-2.64878	-1.45687
H	-7.79541	-0.70847	-1.89231	H	-7.79541	-0.70847	-1.89231
H	-10.2641	0.962016	-1.22024	H	-10.2641	0.962016	-1.22024
H	-8.73591	0.971697	-0.32029	H	-8.73591	0.971697	-0.32029
H	-8.79437	1.586221	-1.97554	H	-8.79437	1.586221	-1.97554
H	-10.5019	-0.57771	-3.28744	H	-10.5019	-0.57771	-3.28744
H	-8.97907	0.045655	-3.93209	H	-8.97907	0.045655	-3.93209
H	-9.19308	-1.69442	-3.69412	H	-9.19308	-1.69442	-3.69412

Conformer 7		Conformer 8
0	**NImag**	0
-767.228437	**Σ Electronic and thermal Free Energy**	-767.229145
0.0084	**mol Fraction**	0.0177

Conformer 9				Conformer 10			
**Atom**	**X**	**Y**	**Z**	**Atom**	**X**	**Y**	**Z**
C	-2.73705	4.733552	-3.44251	C	-5.78774	2.91755	-3.70635
N	-3.50107	3.657487	-3.31039	N	-5.39067	2.763819	-2.45009
N	-2.3405	5.404411	-2.36519	N	-5.53922	1.967006	-4.60282
N	-2.36782	5.147579	-4.65505	N	-6.46382	4.006494	-4.06892
C	-3.95289	3.133912	-2.02474	C	-4.72457	1.565735	-1.94644
C	-4.73799	1.85124	-2.24028	C	-4.57441	1.656706	-0.43637
C	-5.20518	1.282041	-0.91061	C	-4.02258	0.361392	0.155951
N	-5.96287	0.062791	-1.12631	N	-4.93863	-0.75315	-0.01921
C	-6.5559	-0.58836	-0.10605	C	-6.06712	-0.86645	0.714209
C	-7.35782	-1.82482	-0.46987	C	-7.03372	-1.95907	0.294515
O	-6.47386	-0.17965	1.048257	O	-6.31568	-0.10244	1.640922
C	-8.85056	-1.4712	-0.37857	C	-8.29648	-1.29846	-0.2798
C	-6.97489	-2.98084	0.452423	C	-7.36838	-2.85539	1.483997
C	-9.76883	-2.62226	-0.78311	C	-8.00749	-0.29372	-1.39207
C	-11.2288	-2.20844	-0.98763	C	-9.25102	0.336537	-2.02449
C	-11.8306	-1.60026	0.278235	C	-10.0646	-0.67967	-2.82404
C	-12.0563	-3.40687	-1.44648	C	-8.84079	1.507869	-2.91516
H	-3.81372	3.195036	-4.15207	H	-5.53582	3.532193	-1.81084
H	-1.78925	6.242705	-2.45787	H	-5.85845	2.061795	-5.55374
H	-2.70844	5.188388	-1.45305	H	-4.93653	1.190256	-4.38536
H	-1.71721	5.909872	-4.75748	H	-6.66059	4.19209	-5.03912
H	-2.52868	4.566271	-5.46223	H	-6.68079	4.726783	-3.39889
H	-4.58298	3.876351	-1.52695	H	-3.7411	1.463161	-2.41426
H	-3.08204	2.934588	-1.39512	H	-5.33132	0.694903	-2.20914
H	-4.10591	1.121703	-2.75384	H	-5.54673	1.863734	0.018308
H	-5.60333	2.055672	-2.87645	H	-3.9033	2.480466	-0.18298
H	-5.83519	2.004666	-0.38648	H	-3.84667	0.499623	1.221847
H	-4.3482	1.071603	-0.2649	H	-3.07471	0.094707	-0.31145
H	-6.02791	-0.3186	-2.05747	H	-4.7953	-1.39345	-0.78397
H	-7.13094	-2.10005	-1.50437	H	-6.56741	-2.55906	-0.49235
H	-9.05093	-0.60965	-1.02566	H	-8.82343	-0.79313	0.53589
H	-9.06346	-1.15619	0.646449	H	-8.95138	-2.09517	-0.64054
H	-5.89901	-3.16099	0.428876	H	-6.47871	-3.35663	1.868198
H	-7.26207	-2.75251	1.47997	H	-7.80412	-2.26127	2.288126
H	-7.47753	-3.89793	0.146326	H	-8.0901	-3.6173	1.186397
H	-9.73617	-3.41093	-0.0233	H	-7.41539	-0.77468	-2.1827
H	-9.39583	-3.06778	-1.71252	H	-7.38778	0.513651	-0.98359
H	-11.2539	-1.44967	-1.77875	H	-9.87999	0.724576	-1.21475
H	-11.754	-2.30717	1.110223	H	-9.45414	-1.10114	-3.62832
H	-11.3241	-0.67926	0.570251	H	-10.4202	-1.50275	-2.20298
H	-12.8873	-1.36828	0.131021	H	-10.9365	-0.2053	-3.27859
H	-12.0646	-4.17989	-0.67257	H	-8.17711	1.160921	-3.71397
H	-13.0909	-3.11907	-1.64355	H	-9.70918	1.97754	-3.38134
H	-11.6452	-3.84717	-2.35734	H	-8.30941	2.270217	-2.3401

Conformer 9		Conformer 10
0	**NImag**	0
-767.229467	**Σ Electronic and thermal Free Energy**	-767.231482
0.0250	**mol Fraction**	0.2114

**Conformer 11**				**Conformer 12**			
**Atom**	**X**	**Y**	**Z**	**Atom**	**X**	**Y**	**Z**
C	-6.11868	3.126631	-3.11886	C	-3.47087	4.672268	-3.13058
N	-5.12536	2.273665	-2.90736	N	-4.2815	3.655049	-2.87324
N	-6.94292	3.445434	-2.12411	N	-2.15334	4.512766	-3.03993
N	-6.3015	3.654256	-4.3296	N	-3.97275	5.857147	-3.47761
C	-4.90549	1.578404	-1.64034	C	-3.82651	2.327916	-2.46782
C	-3.88262	0.469179	-1.83527	C	-5.02623	1.454818	-2.14203
C	-3.72548	-0.39629	-0.58655	C	-4.58658	0.058336	-1.71135
N	-4.9053	-1.19965	-0.31346	N	-5.71623	-0.77406	-1.34558
C	-5.91267	-0.78406	0.482854	C	-6.33641	-0.64543	-0.15209
C	-7.13048	-1.69151	0.56646	C	-7.60387	-1.46004	0.031265
O	-5.86799	0.284796	1.084424	O	-5.91587	0.112428	0.715712
C	-8.31383	-0.96614	-0.0898	C	-8.79481	-0.51848	-0.22501
C	-7.42413	-2.02498	2.02783	C	-7.64104	-2.07831	1.425863
C	-8.13588	-0.74564	-1.59366	C	-10.1651	-1.18159	-0.07592
C	-8.9905	0.389185	-2.1603	C	-10.3879	-2.43341	-0.93016
C	-10.4828	0.099421	-2.01432	C	-10.163	-2.15638	-2.41564
C	-8.62927	0.653818	-3.61955	C	-11.7964	-2.97586	-0.69645
H	-4.46532	2.125054	-3.65782	H	-5.27313	3.799787	-2.99807
H	-6.70423	3.2283	-1.16942	H	-1.52982	5.273047	-3.26065
H	-7.69658	4.097293	-2.27537	H	-1.74971	3.596761	-2.92944
H	-7.07806	4.27167	-4.50511	H	-3.36494	6.646169	-3.62617
H	-5.83026	3.263743	-5.13012	H	-4.96092	6.037639	-3.40085
H	-5.8577	1.162901	-1.30468	H	-3.18616	2.424814	-1.58718
H	-4.55588	2.280921	-0.87867	H	-3.24267	1.876688	-3.27543
H	-2.91393	0.908026	-2.08657	H	-5.67358	1.376009	-3.01921
H	-4.18795	-0.16387	-2.67316	H	-5.60313	1.909603	-1.3335
H	-3.53325	0.223264	0.289062	H	-3.92205	0.12622	-0.84894
H	-2.87967	-1.06938	-0.71733	H	-4.04589	-0.439	-2.51651
H	-5.01822	-2.06712	-0.81528	H	-6.12898	-1.36981	-2.04615
H	-6.929	-2.61502	0.015476	H	-7.61217	-2.25767	-0.71716
H	-8.4348	-0.00182	0.415433	H	-8.69819	-0.09012	-1.22724
H	-9.22178	-1.54193	0.104995	H	-8.72349	0.315285	0.479461
H	-6.57831	-2.52644	2.500709	H	-6.74397	-2.66909	1.61623
H	-7.63707	-1.11084	2.583635	H	-7.70333	-1.29629	2.183863
H	-8.29294	-2.68088	2.09332	H	-8.50606	-2.73339	1.532044
H	-8.36141	-1.67363	-2.13251	H	-10.9315	-0.44415	-0.3401
H	-7.08578	-0.52449	-1.81311	H	-10.3364	-1.4353	0.974497
H	-8.7581	1.292776	-1.58375	H	-9.67584	-3.20384	-0.61217
H	-10.7414	-0.82155	-2.54545	H	-10.8161	-1.34532	-2.752
H	-10.7703	-0.02177	-0.96852	H	-9.13237	-1.86688	-2.62963
H	-11.0807	0.910146	-2.43532	H	-10.3908	-3.04133	-3.01302
H	-8.88139	-0.21186	-4.23893	H	-12.5427	-2.24488	-1.02067
H	-9.17025	1.517935	-4.01148	H	-11.9625	-3.89721	-1.25801
H	-7.55763	0.841004	-3.73379	H	-11.9684	-3.18543	0.361436

Conformer 11		Conformer 12
0	**NImag**	0
-767.229621	**Σ Electronic and thermal Free Energy**	-767.228310
0.0294	**mol Fraction**	0.0073

Conformer 13

**Atom**	**X**	**Y**	**Z**
C	-2.28085	4.146933	-3.09088
N	-3.0896	3.131129	-2.82226
N	-2.73472	5.394613	-3.02687
N	-1.01462	3.92218	-3.44346
C	-4.50269	3.277845	-2.48351
C	-5.12629	1.907078	-2.2841
C	-6.60085	2.029516	-1.9104
N	-7.21391	0.734463	-1.68792
C	-7.05222	0.058637	-0.5301
C	-7.76418	-1.27837	-0.4319
O	-6.39846	0.519343	0.400205
C	-9.00343	-1.08895	0.459395
C	-6.81188	-2.33925	0.115475
C	-9.83808	-2.35657	0.650311
C	-10.2982	-3.04161	-0.64039
C	-11.0761	-2.0875	-1.54521
C	-11.1453	-4.26698	-0.30328
H	-2.70105	2.199354	-2.84584
H	-2.14639	6.17115	-3.28283
H	-3.68827	5.5915	-2.77151
H	-0.36078	4.685257	-3.51324
H	-0.63758	2.988141	-3.43019
H	-5.01385	3.806908	-3.29283
H	-4.59759	3.866895	-1.56706
H	-4.60348	1.375386	-1.48568
H	-5.03024	1.322753	-3.20234
H	-7.15518	2.533795	-2.70189
H	-6.70507	2.616729	-0.99675
H	-7.71449	0.294695	-2.44385
H	-8.08815	-1.57068	-1.43472
H	-9.62707	-0.29691	0.034503
H	-8.66578	-0.73176	1.436246
H	-5.90261	-2.39737	-0.48482
H	-6.53098	-2.09961	1.142029
H	-7.28532	-3.32131	0.106492
H	-10.7263	-2.09525	1.236613
H	-9.27762	-3.07789	1.252627
H	-9.41266	-3.38778	-1.18596
H	-11.9341	-1.67206	-1.00781
H	-10.4612	-1.25372	-1.88991
H	-11.4523	-2.61064	-2.42652
H	-12.0552	-3.96546	0.223618
H	-11.4423	-4.80215	-1.20731
H	-10.5988	-4.96044	0.339453
**NImag**			0
**Σ Electronic and thermal Free Energy**			-767.228986
**mol Fraction**			0.0150
